# Providers’ perceptions of communication with patients in primary healthcare in Rwanda

**DOI:** 10.1371/journal.pone.0195269

**Published:** 2018-04-04

**Authors:** Vincent Kalumire Cubaka, Michael Schriver, Philip Cotton, Laetitia Nyirazinyoye, Per Kallestrup

**Affiliations:** 1 School of Medicine and Pharmacy, University of Rwanda, Kigali, Rwanda; 2 Centre for Global Health, Department of Public Health, Aarhus University, Aarhus, Denmark; 3 School of Public Health, University of Rwanda, Kigali, Rwanda; The Chinese University of Hong Kong, HONG KONG

## Abstract

**Background:**

Delivery of effective healthcare is contingent on the quality of communication between the patient and the healthcare provider. Little is known about primary healthcare providers’ perceptions of communication with patients in Rwanda.

**Aim:**

To explore providers’ perceptions of patient-provider communication (PPC) and analyse the ways in which providers present and reflect on communication practice and problems.

**Methods:**

Qualitative, in-depth, semi structured interviews with nine primary health care providers. An abductive analysis supplemented by the framework method was applied. A narrative approach allowed the emergence of archetypical narratives on PPC.

**Results:**

Providers shared rich reflections on the importance of proper communication with patients and appeared committed to making their interaction work optimally. Still, providers had difficulty critically analysing limitations of their communication in practice. Reported communication issues included lack of communication training as well as time and workload issues. Two archetypes of narratives on PPC issues and practice emerged and are discussed.

**Conclusion:**

While providers’ narratives put patients at the centre of care, there were indications that patient-provider communication training and practice need further development. In-depth exploration of highlighted issues and adapted strategies to tackle communication drawbacks are prerequisites to improvement. This study contributes to the advancement of knowledge related to communication between the patient and the provider in a resource-limited setting.

## Introduction

A vast array of literature shows that proper communication between the patient and the healthcare provider, often referred to as patient-provider communication (PPC), is essential for a successful and sustainable response to patients’ health problems [1–6].

Throughout their education, future healthcare providers (referred to as providers) are required to learn and develop skills necessary for effective interaction with patients in order to deliver safe and appropriate care [7,8]. Much attention is traditionally put on the output of patient interaction, such as how to attain a proper medical history and conduct a physical exam [9]. Increasingly, especially in high-income countries, the process, quality and dynamics of the interpersonal interaction are the focus points of training and reflection. There are anecdotal indications this is not the case in low-income countries. A recent review on non-technical skills among providers in low- and middle-income countries failed to identify a single study from Africa that focused on communication skills or training in the interaction with patients [10].

The current understanding of PPC relies largely on evidence from high-income countries, where there have been numerous efforts to investigate determinants and outcomes of PPC, including the influence of contextual factors like culture, language, health literacy and working conditions [2,8,11,12]. The concept of patient-centred care is generally a leading model for healthcare, and essential to the practice of PPC. While the concept is ill-defined, it is often described by its components that include 1) attention to patient needs, perspectives and experiences, 2) opportunity for patient participation and involvement, and 3) enhanced partnership and understanding in the patient-provider relationship [13,14].

Reports on abusive forms of patient care in Africa have been published [15]. However, research focusing on PPC in primary care in Africa is scarce and from our knowledge no study has investigated providers’ perceptions of PPC in primary healthcare in Rwanda. This is important in order to identify how the training of providers in patient communication and interaction may best respond to existing challenges.

Primary healthcare in resource-constrained settings is often overburdened and under-resourced [10]. This may imply that precarious and stressful working conditions affect health outcomes such as through insufficient quality in the PPC. Poor working conditions could also influence providers’ motivation to engage in constructive communication, thus exacerbating poor outcomes [16].

In Rwanda, there are 495 primary health care facilities called health centres, which care for more than 90% of all ambulatory patients in the country [17]. Health centres are managed by nurses, the majority of whom have a secondary school level-based nursing degree with no or limited communication skills training [18]. Rwanda has one local language, Kinyarwanda.

## Methods

### Study design and question

As we found no studies on PPC from Rwanda we applied an exploratory qualitative approach articulated around the PPC literature and focusing on the following broad research questions:

A) What are the main PPC components perceived and valued by providers?
B) How do providers present and reflect on communication problems and practice?

### Sampling

Healthcare providers were purposively selected to participate in individual, in-depth, semi-structured interviews. These were nurses working in outpatient departments (OPD) of health centres in Rwanda and who have experienced PPC. We selected half from rural and half from urban health centres as their patients may differ in terms of socioeconomic status. The health centre manager put us in contact with providers who had the required profile. We only included those who were available and who agreed to participate after being informed about the study and agreed a mutually appropriate time to meet.

### Data collection

The interviews were carried out in May 2016 at health centres. A trained interviewer conducted the interviews, attended by one researcher (VKC). The interview guide was developed using PPC literature, discussed in the research team and translated to Kinyarwanda. Interviews were conducted in Kinyarwanda, audio recorded and lasted on average 60 min (shortest 44, longest 87).

The material was transcribed in Kinyarwanda and translated to English verbatim by a professional translator, and anonymized S1 Dataset.zip. A random page of each translated interview transcript was double-checked by the interviewer against the Kinyarwanda transcript and audio recording, and there were no substantial corrections.

Immediately after interviews, the overall impression of the data and salient points were discussed between the interviewer and two researchers (VKC and MS). When they began to agree that several insights were recurring and that no new important information appeared to be emerging, another three informants were included to verify this impression. Thus a total of 9 interviews (6 plus 3) were conducted. Further, a comprehensive understanding of the data was obtained during the analysis, with recurrence of similar insights for emerging themes and narratives, making us confident to claim data saturation.

### Data analysis

**Fig 1:**
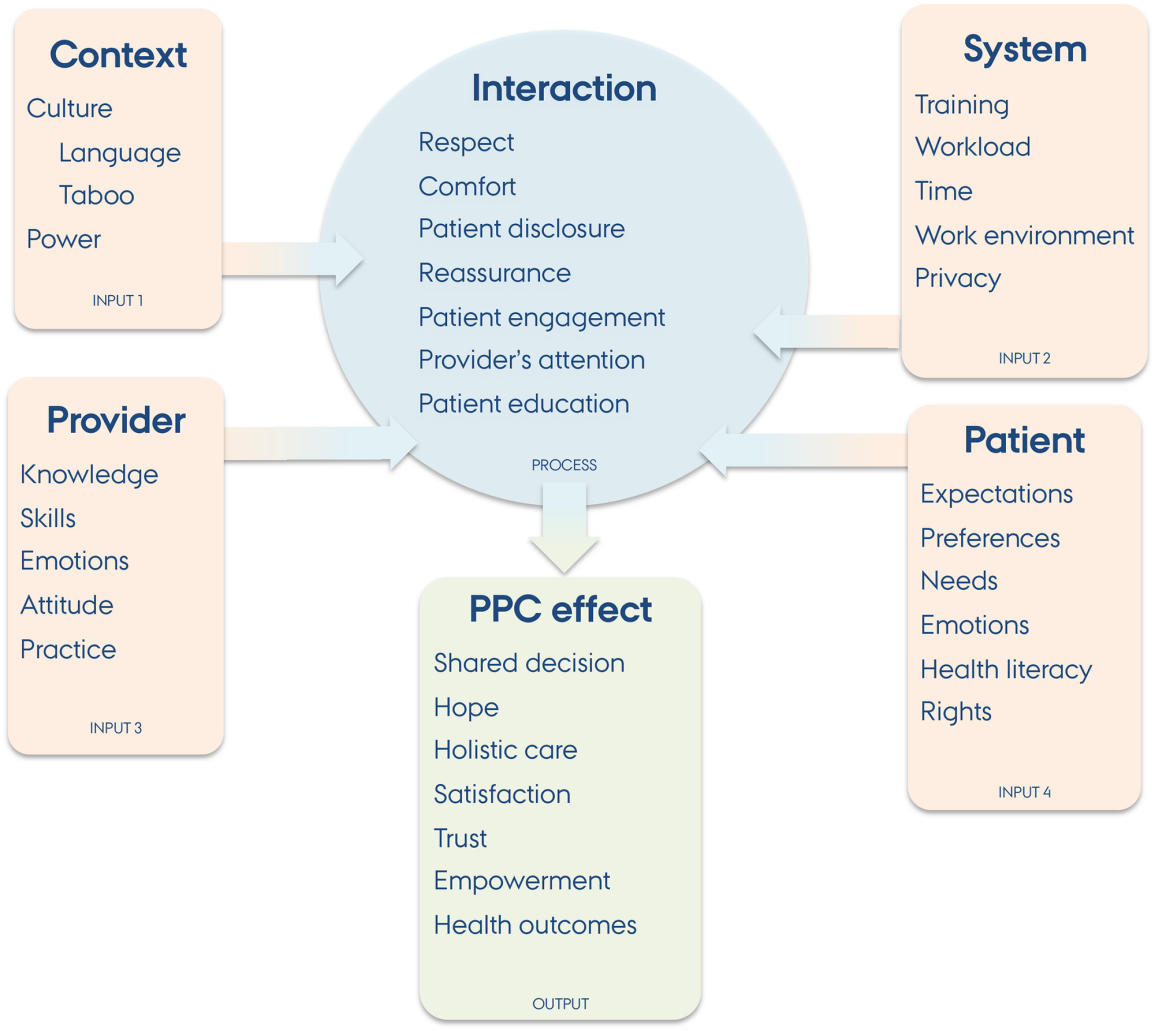
Emerging themes applied to the input-process-output model.

To explore research question A) we applied an abductive analysis (a hybrid of deductive and inductive analytical approaches) which is constructed from a grounded theory foundation [19]. This was supplemented by the framework method, which is based on thematic analysis [20]. In practice, two researchers (VKC and MS) separately familiarized themselves with the written material and open coded it. This inductive process helped to identify potentially relevant themes. Through discussions, the two researchers selected, defined and refined emerging key themes and categorised them into a thematic framework. One researcher (VKC) systematically indexed the text from all the transcripts using the thematic framework. This process helped to reorganise data to facilitate the interpretation. Emerging themes where re-arranged under a previously elaborated conceptual model in order to put results into a larger perspective (Fig 1). This conceptual model was deductively developed from PPC theories [21–24] and empirical knowledge and labelled as *input*, *process* and *output* of PPC.

Concerning research question B), the analysis was inspired by a narrative enquiry approach [25]. We focussed on how practice and problems were presented, i.e. the way providers would tend to talk about these. These narratives were discussed among researchers and refined into two archetypical narratives used to deductively code the material to deepen their interpretation.

Preliminary findings and conclusions were reviewed by co-authors, and disagreements settled. The qualitative analysis software MaxQDA11 facilitated the analysis process.

### Ethical consideration

The Institutional Review Board of the College of Medicine and Health Sciences of the University of Rwanda approved this study (CHMS/IRB/216/2015). Participants were informed about the study details and signed an informed consent form prior to their participation. Interviews were conducted before or after consultation hours.

## Results

In an aim for greater clarity, results are presented as exemplars of providers’ views and narratives, backed by illustrative quotes. Some themes overlap. The use of brackets […] represents an omitted text part, which is only applied if it does not alter the meaning. There are no gender specific pronouns in Kinyarwanda and so results are presented as ‘her/him’ and ‘she/he’. Characteristics of participants may be found in Table 1.

**Table 1:** Participant characteristics.

Provider-ID	Age	Gender	Highest nursing education level	Years of experience in OPD	HC location
Pr 1	33	Male	A2	5–10	Rural
Pr 2	32	Female	A2	3–5	Rural
Pr 3	57	Male	A1	3–5	Rural
Pr 4	30	Male	A2	5–10	Rural
Pr 5	36	Female	A2	5–10	Rural
Pr 6	43	Female	A2	0–2	Urban
Pr 7	39	Female	A2	> 10	Urban
Pr 8	33	Female	A2	5–10	Urban
Pr 9	29	Female	A1	0–2	Urban

Pr: Provider, A2: Basic nursing certificate attained during secondary school, A1: Full nursing degree.

Of the nine participants, six were female, seven had three or more years of experience and seven had the basic A2 nursing level.

For research question A, three main themes emerged and each is further subdivided in sub-themes. Themes with exemplary quotes are presented in Table 2. Quotes are referred to in the text using the corresponding number in the table.

**Table 2:** Emerging themes with illustrative quotes.

Themes	Subthemes	Quotes
**What makes the interaction work**		
	**Patient expectations**	
		*1*. *It is only a few health care providers who can do what patients wish [*…*] Implementing patient preferences is not the culture Pr 4*
		*2*. *We must respect the patient’s preferences as long as they make a choice that lead to the solution of the problem that they have*. *Pr3*
		*3*. *During our conversation*, *I ask her/him*: *what is helpful for you*, *what is going well*, *what do you think it is not helpful for you*? *This is a guide to have a mutual understanding so that you provide a good care for her/him*. *Pr 2*
	**Patient comfort**	
		*4*. *Health care providers should remain humble and kind towards patients so that they can feel comfortable enough to tell them about their problems*. *Pr 5*
		*5*. *There are health care providers whose behaviors are so strange that patients do not feel satisfied or they do not feel comfortable with them*. *Pr8*
		*6*. *We should feel comfortable each other*, *familiar one another so that the patient feels talking everything*. *Therefore*, *such a conversation will be of a great value*. *Pr1*
	**Hope**	
		*7*. *The friendly conversation is already half the cure of their illness [*…*]*. *They go home saying*, *“There is hope of recovery because what the health care provider has done for me is good*. *Pr7*
		*8*. *An opportunity of welcoming a patient and having a conversation with her/him*, *it is said that it creates a hope for the patient*. *That hope is like a half for the patient to recover from her/his illness*. *Pr2*
		*9*. *When you make him/her understand it enables him/her to have acceptance and life continue*. *Pr9*
	**Provider attention**	
		*10*. *The best thing is to let the patients express themselves […] If the patients talk when I am listening to my mobile phone*, *the patients will realize that I am not listening to them*. *Pr 3*
		*11*. *When someone comes suffering you have to show that you are listening to him/her*. *Pr9*
		*12*. *When you give attention to someone it shows that you care for him/her which shows that you don’t value other things than him/her*. *Pr6*
	**Emotions**	
		*13*. *When a patient cries*, *you normally let them cry for a while until you see that they have cooled down to some extent and then you try to resume the conversation*. *Pr 1*
		*14*. *The health care provider should keep a balanced feeling*. *Pr 8*
		*15*. *I can look at emotions in two ways*: *an emotion that shows that you feel the patient’s pains and the emotions that make you blame the patient*, *which is bad and unfavourable for the patient*. *Pr 3*
		*16*. *That is why there is a need of skills in managing the emotions*, *skills in how to talk to patients Pr4*
**What comes out of effective interaction**		
	**Disclosure, trust and confidentiality**	
		*17*. *They cannot tell you anything unless they are comfortable with you*. *Pr 7*
		*18*. *Health care providers should keep patients’ stories with high confidentiality*. *Pr 8*
		*19*. *The patient can evade to explain well about their illness because they see that there is no secrecy there*. *Pr 4*
		*20*. *There is a good relationship between the health care provider and the patient when they have had a good conversation*. *Additionally*, *the conversation allows mutual understanding and trust*. *Pr3*
		*21*. *We play two roles at the health centre [*…*]*. *The conversation that we have is about the patient’s illness or about the advice they are seeking*. *Pr 4*
	**Patient engagement**	
		*22*. *It is necessary that people also get involved in the solution of their problems so that we have a long term solution for the illnesses*. *Pr 3*
		*23*. *You help someone else to help themselves [*…*]*. *We make a common decision so that we avoid any conflicts between us [*…*]*. *Both the patient and health care providers reach an agreement before doing anything*. *Pr 8*
		*24*. *Explaining to them helps them to know how to care for their health or if they have a particular problem*, *they get to know how they have to behave according to their situation*. *Pr 4*
	**Patient education**	
		*25*. *When you do not explain to them*, *they can relapse anytime and they can return to see you and yet*, *the more patients return to the health facility*, *the more our work increases*. *Pr 1*
		*26*. *It would be necessary if you provide the patient with information about his/her health [omission] It would help her/him recover from the illness*, *and help for the prevention for other illnesses either for her/himself or for his/her family members or his/her environment*. *Pr2*
		*27*. *What I see is that they [patients] don’t even want to ask information […]*. *Even though they say that they are given little information*, *you find out that they are not interested in knowing more information than what was given*. *Pr 6*
	**Holistic care**	
		*28*. *If you have a long conversation*, *they can tell you many things concerning their life in the community*, *and from that story you can for example find out that it is an illness that is ravaging the whole community*. *Pr 1*
		*29*. *Someone may come complaining of headache and the headache being caused by a great sorrow that they have been experiencing*. *So I can give them pills for headache but in reality I have not treated the root cause of the headache*. *Pr8*
		*30*. *You can advocate for them thanks to the conversation that you have had [omission] You can give them advice*, *and as the health care provider you have the opportunity to help them definitively*. *Pr7*
**What surrounds the interaction**		
	**PPC knowledge and practice**	
		*31*. *The course was taught a few hours and people did not attach any big importance to it [*…*] they do not teach you how you will talk to patients*. *Pr 4*
		*32*. *So many people don’t grasp the importance of it to the extent that conversations between patients and health care providers are too short*. *Pr 4*
		*33*. *You can tell them something and ask them to repeat it after a while*. *In that case*, *you can evaluate and see if they have understood what you agreed on*. *Pr 7*
		*34*. *If I know how to do it*, *I will be able to have a quality conversation with a patient*, *and s/he will have a hope*. *Pr 2*
		*35*. *They tell you what goes well and what does not go well in your services and therefore you know whether or not there are things that you have to amend*. *Pr5*
		*36*. *If an angry patient comes in*, *I will remain so kind that they will talk to me in a friendly way once they see that I am also friendly to them*. *Pr8*
	**Time and workload**	
		*37*. *I think the only obstacle to the conversation is time*. *Pr 6*
		*38*. *There is also a time you don’t explain much because you don’t have enough time and make you not do it the way it should be done*. *Pr 9*
		*39*. *We really have very short time*, *but if we use it properly we can have good outcomes Pr 8*
		*40*. *A heath care provider may be tired having a lot of work and feeling tired can cause that they do not talk to the patient effectively Pr 1*
		*41*. *It would be good if the healthcare provider would receive only as few patients as he will be able to have enough conversation with*. *Pr 7*
		*42*. *They should also increase the number of staff at health centers*. *Pr 2*
	**Patients’ rights**	
		*43*. *It is the patient’s right to participate in the health care which they are given*. *Pr 7*
		*44*. *Sometimes they go home knowing that you have treated them but without knowing what you have treated them for*. *Pr 5*
		*45*. *If you do not explain to them their illness*, *they cannot know how to change their behaviour in order to be able to live with their illness*. *Pr 8*
		*46*. *Those who get those explanations are those who are knowledgeable and who have the courage to ask the health care provider*. *Pr 4*
		*47*. *It is difficult to inform sad information but we have to do it*. *Pr 6*
		*48*. *You can tell a patient that you are going to do a test for HIV/AIDS and they have the right to refuse*. *If they refuse*, *you cannot do the test … when they insist that they need a transfer*, *you let them go because it is their right*. *Pr 7*
	**Culture**	
		*49*. *It requires that you try to be like them*, *you speak like them and you show them that you are equal to them*, *it is only then that they can feel free to talk with you*. *Pr 3*
		*50*. *It is very difficult to explain terms which do not have equivalents in Kinyarwanda*. *Pr 4*
		*51*. *In case you don’t know the [*…*] metaphoric expressions of the language*, *you can treat the patients in a wrong way*. *Pr 8*
		*52*. *The Rwandan culture includes having the same language*. *[omission] sharing the same language helps to understand one another in a whatever way*. *Pr 2*
	**Power**	
		*53*. *It happens that health care providers make themselves very important people to the extent that patients feel afraid when they want to see them*. *In that case*, *you understand that the patient cannot be comfortable with the health care provider Pr 1*
		*54*. *You should be the patient’s parent Pr 3*
		*55*. *They feel that you are very highly important and therefore they cannot tell you anything*. *Pr7*
	**Categorising patients**	
		*56*. *It is very important that health care providers learn how to better engage different types of patients in conversation Pr 3*
		*57*. *Patients do not resemble*, *and each patient is unique*. *Pr 8*
		*58*. *The first thing that I do is to respect what they say and do not blame them or shout at them; I just listen to them and talk to them Pr 4*
		*59*. *You try to ask them questions that they are able to answer [omission] which are related to their level of knowledge*. *Pr 5*

For research question B, 4 archetypes emerged from the narrative analysis. Quotes that exemplify the four archetypes are presented at the end of the result section (Table 3).

**Table 3:** Narrative archetypes in presentations of problems and practice of PPC.

Approach to PPC Problems	Approach to PPC Practice
**Introspection** *“I feel that I don’t have comprehensive skills*, *I feel that I am lacking in this subject*.*”* Pr 8 *“If I know how to do it*, *I will be able to have a quality conversation with a patient”* Pr 2	**Seeking** *“I let them talk and I understand what they are telling me*.*”* Pr 4 *“During our conversation*, *I ask her/him*: *what is helpful for you*, *what is going well*?*”* Pr 2
**Extrospection** *“The problem is mostly due to lack of time*. *But conversing with patients is not a problem for me*.*”* Pr 1 *“I think the only obstacle to the conversation is time*.*”* Pr 6	**Knowing** *“If […] their suggestion is similar to something that I was planning to do for them […] I do as they wish”* Pr 1 *“You should be the patient’s parent”* Pr 3

### What makes the interaction work

Providers shared their insights into determinants of effective interactions. They gave several accounts, which demonstrated the value they give to communication. A provider said: *“One should recognize the importance of the conversation that one has with patients because it is basically the foundation of the successful treatment*.*”* Pr 4.

#### Patient expectations, comfort and hope

Providers believed they should be aware of patients’ expectations and wishes. Some thought that they should respect these expectations as long as they were realistic and legitimate, as it promotes collaboration and mutual understanding. Some even criticized health care services for not meeting patients’ wishes (Quotes 1–3). Patient comfort and satisfaction were seen to depend on providers’ ability to detect and effectively respond to patients’ expectations and wishes.

Providers too felt more comfortable and satisfied when their patients were comfortable and satisfied.

Providers believed they have a key role to play in making patients feel comfortable during a consultation. This is necessary for creating an atmosphere where patients are willing to share important information. Providers also mentioned that a proper welcoming of the patient is a prerequisite to effective interaction, helping the patient feel comfortable and able to express her/himself openly. This requires providers to act with humility (Quotes 4–6). Several providers claimed this attitude was not well enough expressed in the current healthcare system.

Hope was seen as important for patients and for their recovery. A friendly and kind conversation can give hope and reassurance, and some providers would regard this as a prerequisite for effective treatment (Quotes 7–9).

#### Provider attention and emotions

Providers found it important that patients get their undivided attention, but revealed that this is not always the case. Attention means listening actively with no distractions, and avoiding interruptions when the patient is talking. This is not always practiced, and providers pointed in particular to a high number of patients as the cause, pushing providers to rush. Other causes were distractions during the patient consultation. Some directly stated that it is a problem to patients when providers tap their mobile telephone while a patient is talking (Quotes 10–12).

Providers described the importance of being attentive to patients’ feelings, ideas, worries and moods, and to deal with these appropriately. Providers believed patients would be more open to share their concerns if they found the provider attentive towards and caring about their feelings. Some providers said that when patients express their feelings, it is an opportunity to comfort and show compassion (Quote 13).

Providers recognized that their own emotions may positively and negatively affect communication with patients. Some providers said being aware of their own emotions and knowing how to control and use them is part of being a health professional, and is necessary for optimal communication with patients.

Several saw it best if they could express a positive emotional response to patients’ emotions, to help them feel more comfortable and open, as well as to generate hope. For instance, if a patient cries one should show compassion and perhaps optimism. Some providers found it difficult to deal with their own emotions and recommended simply not expressing them. In the interaction with a patient they saw their role as neutral and stabilising, to avoid the risk of influencing patients inappropriately (Quote 14).

Providers also described expression of emotions as a double-edged sword that may have both beneficial and harmful consequences (Quote 15). Positive emotions can convey compassion, empathy and hope, whereas negative emotional reactions to stressful working conditions might be interpreted as indifference, negativity and doubt. Providers themselves requested more training in how best to manage both their own and patients’ emotions in the patient-provider interaction (Quote 16).

### What comes out of effective interaction

This section presents providers’ perceptions on outcomes of effective interactions.

#### Disclosure, trust and confidentiality

Providers thought that the abilities of patients to disclose information depends upon the attitude and behaviour of the providers they consult (Quote 17). For instance, rudeness was said to prevent disclosure of information, while interest, active listening and humility was said to promote it. Effective communication would therefore help disclose sensitive issues, such as problems relating to genital organs or sexual activity.

Providers linked patient comfort to trust and mutual understanding, resulting in a constructive patient-provider relationship. Some providers saw themselves as *“keepers of secrets”*, and emphasized this role as crucial in gaining patients’ trust (Quote 18).

Privacy was often described as important for trust and confidentiality. Privacy in the consultation is a challenge, as it may not always be guaranteed because of structural or organizational problems, which means other patients may sometimes be able to overhear a consultation. This was found unacceptable for several reasons, such that it inhibits the quality of the communication (Quote 19).

Related to trust, providers described that many patients would come with problems of social or psychological nature. Providers described this as seeking advice, and saw their role as generalist advisors (Quotes 20, 21).

#### Patient engagement and education

In general, providers acknowledged the value of actively involving patients in their care, and including them in making decisions. This was seen to help patients to better take control of their health and safeguard sustainable solutions to their health problems (Quote 22). However, some providers had reservations for patient engagement. It appeared that the concept of shared decision-making was not well understood by some providers, who needed examples to see what it meant. Some mentioned that they should be careful with involving patients, as patients might make harmful decisions. Several providers assumed they knew what was the “right decision” for patients, and therefore saw patient engagement to require a high level of information and guidance toward that “right decision”.

Other providers called for more two-way communication, always involving patients by actively seeking their reactions and concerns. A few providers explained that the providers’ main task is to help patients help themselves, as patients usually have the answer to their own problems. Some found shared decision-making important as it minimizes the risk of conflict between the patient and the provider through agreement (Quote 23).

Providers generally felt they had a responsibility to help educate patients about health related topics, and wished they could practice this effectively, as both patients and the health system would benefit (Quote 24). Patient education was linked to patients’ right to information, and was seen to help patients deal with the current problem as well as prevent future health problems, thus potentially reducing providers’ workloads (Quotes 25, 26). Some providers mentioned patient education could fail without full involvement of the patient. Others seemed to put the responsibility for initiating educative conversations on the patients’ shoulders, thereby initiated by patients’ interests and needs (Quote 27).

#### Holistic care

Providers made several direct and indirect references to the importance of holistic care. One notion was that conversations with patients should extend beyond their biological and physical concerns to reveal mental, social and community-related issues that patients might not otherwise share, but may be at the root of their health problems (Quotes 28, 29). This holistic care concept was linked with the providers’ role as advisers and advocates for patients in their community, and with awareness that ideal care would seek sustainable solutions to health problems (Quote 30). At the same time, these concepts were discussed at a theoretical or ideological level, whereas it was difficult to find examples of how it was practiced as part of daily work. For instance, holistic care was often related to having long conversations with patients, something almost all providers said was not possible, as they claimed to see too many patients each day.

### What surrounds the interaction

This section reports other perceived factors that surround the interaction, influence it, and would require particular attention.

#### PPC knowledge and practice

In general, providers felt they had insufficient knowledge and skills to effectively communicate with patients. Some appeared challenged to reflect and talk about communication, and understood the topic initially to be about informing patients in a medically correct and updated way about health and care, rather than about the way a conversation and interaction was conducted. Others said it was often wrongly assumed that they know how to communicate well.

All informants denied receiving formal training on how to communicate with patients. Those who received some kind of communication training described it as superficial and without practical value (Quote 31). This was felt to be a lack, which could create inconsistencies in how providers interact with patients. Providers felt they had to rely on common sense in their communication, and felt they should have had more training during their education, backed-up by formal and informal trainings during their practice. Some expressed this in detail and at length (Quotes 32).

Providers often mentioned that patients are different and they would like to be able to talk to everyone effectively. They would like to learn different styles of communication to approach different patients. Providers shared some communication techniques they used without having learned them through formal training. For example, to make sure patients understood messages well they would ask them to repeat (Quotes 33, 34). Also, in approaching dissatisfied or angry patients, it was found effective to maintain a very kind and humble attitude. Providers requested feedback from patients about the quality of the care they provide. They believed it could help them to know what is perhaps going wrong in their interactions, and to improve their care (Quotes 35, 36).

#### Time and workload

By far, the most commonly perceived constraint for optimal patient communication and interaction was about time, having a high number of patients to see each day (Quote 37). Several providers said this entailed having to see more than 50 patients a day. Thus, effective use of time was highly important to providers (Quotes 38, 39). The consequence of time constraints was not only that of having a few minutes for each patient but was described as causing stress and exhaustion among providers, which may negatively influence the interaction (Quote 40). Some providers went as far as to describe the interaction as superficial. To some the problem would not be overcome until increasing the number of providers (Quote 41, 42).

#### Patients’ rights

Directly and indirectly, providers expressed that basic respect for patients and their rights is at the core of their work. These include the right to information, the right to make choices, and respect for patient autonomy. These rights are often challenged by systemic and organizational factors, as well as by cultures of practice (Quote 43).

While recognizing that sharing information does not always happen, providers believed patients should be informed, as needed, about their health problem, the potential solution and prevention strategies (Quote 44). Knowing what is wrong and how to deal with it can give reassurance and hope, and help the patient cope with the health problem. Providers acknowledged they do not inform all patients as systematically as they would like, and mentioned that only educated people tend to ask for information (Quotes 45, 46). Providers also thought that breaking bad news, as part of sharing information requires particular skills and should not be considered an exception to this right although it is found difficult (Quote 47). As one provider said, it is a matter of *“finding the right way to do it”* Pr 9.

It is also worth noting that no provider described it as among their tasks to seek informed consent from patients.

It was an uncontested belief among providers that patients have the right to make choices, which must be taken into consideration during their interaction. Some providers added that this is fine as long as it does not constitute a risk to the patient. Others insisted patients’ preferences should always be followed, and used the example of care restrictions concerning blood transfusion among Jehovah’s Witnesses, which they felt they had to respect. They saw it as their responsibility to guide patients in their choice (Quote 48).

#### Culture and power

Providers believed culture and traditions influence PPC. They found it important to respect the culture of the patient, including trying to understand and adapt to it for open and effective communication. This was especially expressed in relation to language as well as topics traditionally seen as taboo (Quote 49).

The national local language (Kinyarwanda) was pointed out as an advantage for communication. However, several medical terms were taught to providers only in English or French, and they found it challenging to convey their meaning in Kinyarwanda (Quote 50). Others had no problems with that. In general, providers found it important to avoid using medical jargon from their education, and instead try to paraphrase it in Kinyarwanda even if it might distort or simplify the message. However, some providers would just use the French term even if patients did not understand.

Culturally there are issues that people would avoid talking about explicitly, such as sexual organs. Because using traditional Rwandan words for sexual organs is inappropriate, providers and patients would use metaphors in speaking about sexual organs or other sensitive matters. For instance, “passing by the under part” for diarrhoea, or “southern part” for sexual organs. Providers seemed to assume patients understood these metaphors well, although one pointed out that misunderstanding could happen (Quotes 51, 52).

Several providers recognized that patients could feel uncomfortable and reluctant to disclose information due to traditional power imbalances in the patient-provider interaction (Quotes 53–55). This was seen as a real problem, although this problem was regarded as smaller than in the past. Providers urged to equalize the power simply by having a friendly and respectful attitude. Some providers would indirectly and with good intentions demonstrate power imbalances, for instance “*You should be the patient’s parent”*. Pr 3.

#### Categorising patients

Although providers recognized that each patient is unique, they tended to talk about patients as belonging to different types, mostly referring to categories of complaints and diseases. Many providers believed they should learn to adapt their communication style to such perceived patient types (Quotes 56,57). Some of the categories mentioned were age groups and patients with social problems, but also particular health problems like diabetes, infertility, epilepsy, unwanted pregnancy, mental disorders, speech or hearing impairment, and blindness. Providers also thought communicating with people with chronic diseases required a different approach than people with acute problems.

Providers called for extra attention to people without formalized education, such as people unable to read or write. They believed the existing power imbalance between providers and patients is higher in patients with a low level of formal education, probably exacerbated by an important knowledge gap between them. Providers felt it may require extra skills and well-thought strategies to communicate effectively with patients with a low level of education. A non-judgement and attentive approach was found important (Quotes 58,59).

## Discussion

The findings highlight different components of PPC perceived as important by providers. They often presented these as theoretical ideals or contextual factors that may affect or be affected by PPC. The narrative analysis revealed two archetypes (Table 3).

Fig 1 represents the emerging key themes re-arranged into the conceptual model of *input*, *process and output*. This gives an overview of salient themes linked to current knowledge on PPC. Most of the themes are closely related to the concept of patient-centred care including paying attention to patient rights, values, expectations, needs and experience; increased opportunities for patient participation and involvement; and enhanced patient-provider relationship. The context where care is delivered may also influence PPC and patient-centred care as highlighted by the informants. This includes systemic and cultural issues [13,14]. Selected ‘surprising’ issues are discussed in the following.

### Communication training

Providers spend a major part of their time in interpersonal interactions with patients, and proper training is necessary for quality of this core clinical task [26]. However, interviewed providers reported that they had not received adequate formal communication training. This gap may reflect the lack of defined and robust communication courses within nurse curricula. Nevertheless, providers in general articulated key components of effective communication that align well with commonly described best practices, while they expressed concerns about putting them into practice [8,27]. The lack of communication skills training may maintain a practice of non-holistic, biomedically oriented care models, which are often authoritarian or paternalistic towards the patient [24,28]. There is a need to examine if communication skills are effectively taught as part of the curriculum, and if students actually learn these, to avoid a null curriculum on this important subject [29].

### Time and workload constraints

Almost all providers cited structural constraints, particularly patient overload, as one of the main challenges. Providers gave descriptions of how to communicate and interact with patients as theoretical ideals, but impossible to implement in a 5–10 minute consultation. Other studies have also described time issues as a challenge to PPC [30–32].

### Patients’ rights

Providers reported that they should respect patients’ rights. However, they were also concerned that patients’ rights should match with providers’ preferences and expectations. This raises the question of how best to uphold these rights in stressful environments with severe resource constraints and lack of robust communication training. In addition, providers recognized that it might be difficult to respect patient autonomy in a context of limited health literacy. Thus, there are indications that care quality can be improved through stronger focus on creating partnerships based on agreement, mutual respect for expectations and shared decision-making [33–35]. Our findings suggest a situation, described in a study in Uganda, in which shared decision-making may be a theoretical ideal rather than implemented practice [36].

### Culture

Culture influences communication, and providers acknowledged this by pointing out that ignoring patients’ cultural and social values and beliefs may be detrimental to PPC and trust [3,37]. All Rwandans speak the same language, Kinyarwanda, which is positive for PPC and unique when compared to surrounding countries. The challenge is that providers are usually trained and assessed in other languages, French or English. Providers recognized the challenge of sharing and explaining health related concepts and terms. Further exploration of the influence of Rwandan culture on PPC is warranted to inform optimal ways to convey medical and scientific information while respecting traditional beliefs that shape health seeking behaviour, expectations and wishes [24].

### Approach to PPC problems

From the analysis of *how* providers discussed communication problems, two divergent narratives appeared, i.e. introspective and extrospective narratives (Table 3). Providers were sometimes critical of themselves, trying to find internal explanations to perceived PPC problems. They would use their privileged position as healthcare providers to deliver a comprehensive and nuanced picture of perceived problems of communication, and identify their own limitations. We consider this an introspective narrative. Sometimes providers presented a more narrow view of PPC problems without alluding to, or reflecting on, their own responsibility and attitude. They would often focus on external factors, particularly the overload and provider shortages to justify PPC weaknesses. They repeatedly used the third person (he, she or they) when describing communication drawbacks, and first person (I, we) when sharing PPC success stories. While they shared pertinent insights on communication, they avoided introspection, thereby limiting the exploration of provider weaknesses. We consider this an extrospective narrative. While these archetypes are not represented in any pure form, they do point to the importance of empowering providers in reflective and self-critical practice, as suggested in a recent review[10].

### Approach to PPC practice

There were differences and inconsistencies in providers’ narratives when describing their PPC practices (Table 3). Sometimes providers presented a narrative where the patient was seen as the person holding the answer to a health problem, and consequently the provider’s role was that of helping patients in seeking those answers. These narratives appeared investigative, based on curiosity and were always patient-centred, trying to engage and involve the patient in care. We consider this a narrative of the provider as someone *seeking* knowledge in the patient interaction. Other times providers expressed worries about involving patients too much in their care for diverse reasons, including patient’s lack of health knowledge and the possibility of patients making decisions to please the provider. They would directly or indirectly express themselves as ‘knowers’: experts with a task to convey their knowledge to patients so patients could improve their health. We consider this a narrative of the provider as someone who has the ability of *knowing* what is best for the patient. Such a *knowing* narrative may be influenced by providers’ own assumptions and stereotypes about patients, with the harmful potential to mislead their decisions and compromise patients’ health [38]. Motivation studies show providers are more likely to assist behavioural changes with a “seeking” than a “knowing” attitude, such as by exploring patients’ own thoughts, feelings and expectations around a health issue before deciding if it makes sense to embark on a motivational approach [39]. The *knowing* narrative may also reflect an aspect of the power dynamic in a paternalistic and authoritative healthcare setting. While this narrative has obvious advantages for both the provider and the patient, it may create patient frustration, discomfort and retraction, making the interaction counterproductive. Some patients may prefer a paternalistic provider and may feel challenged or uncomfortable with the “seeking” approach. This suggests the importance of appropriately balancing these two approaches depending on patient needs, as well as finding strategies to empower patients in self-care [40].

### Strengths and limitations of the study

From our knowledge this is the first study exploring providers’ perceptions of PPC in Rwanda. We used a novel and hybrid strategy to analyse data, the abductive analysis coupled to the framework approach that allowed an informed and methodological exploration of providers’ perceptions based on the current empirical and *a priori* PPC knowledge.

The majority of the respondents were female (67%). As the nursing profession in Rwanda and elsewhere is dominated by women we saw no reasons to sample more men.

The analysis focused only on emerging PPC issues from providers’ perceptions, and as such important views on other aspects of PPC may be missed or minimised. However, we believe salient and emergent themes are current and pertinent in guiding the PPC discourse in Rwanda. Many themes overlapped though they are presented and discussed in separate sections to ease analysis. This artificial division does not necessarily reflect the actual complexity of the communicative experience observed between the provider and the patient.

We have not discussed all the points presented in the results and focused on most salient and pressing issues with the expectation that future studies will deepen the exploration of other PPC aspects.

This study is based on interview data and there may be discrepancies between the information retrieved and the actual practice of PPC in primary health care in Rwanda. However, the study is an important first step in exploring PPC in Rwanda by generating the baseline knowledge to guide future investigations as well as strategies for improving PPC in Rwanda and beyond. Future studies may include participant observation.

## Conclusion

Providers’ perceptions on what works well in their interaction with patients were for the most part in accordance with best practice in the current PPC literature. At the same time, providers were reluctant to criticize their own role and responsibility concerning problems around PPC.

There were several indications that communication quality should be improved to enable effective patient-centred, partnership-based and culture sensitive care. This requires exploration of highlighted communication drawbacks and other factors identified to guide adapted responses to structural and organizational issues, as well as the development of strategies to empower patients with particular attention on patients with low health literacy, who may pose challenges around patient education.

There is also a need to ensure communication skills and other non-technical skills training in the health professions curricula using practical, problem-based, context-oriented and team-based learning approaches, preparing providers to interact as effectively as possible with patients, especially in stressed and resource-constrained environments, toward improving health and life outcomes.

This study contributes to the advancement of knowledge related to the communication between the patients and health care providers in resource-constrained settings.

## Supporting information

S1 Dataset Transcripts of the interviews (English version). (ZIP)

## References

[1] Street RL, Makoul G, Arora NK, Epstein RM. How does communication heal? Pathways linking clinician-patient communication to health outcomes. Patient Educ Couns. 2009;74:295–6. 10.1016/j.pec.2008.11.015 19150199

[2] Stewart MA. Effective physician-patient communication and health outcomes: a review. CMAJ. 1995;152(9):1423–10. 7728691 PMC1337906

[3] Matusitz J, Spear J. Effective doctor-patient communication: an updated examination. Soc Work Public Health [Internet]. 2014;29(February 2015):252–14. Available from: http://www.ncbi.nlm.nih.gov/pubmed/24802220 10.1080/19371918.2013.776416 24802220

[4] Zolnierek KBH, Dimatteo MR. Physician communication and patient adherence to treatment: a meta-analysis. Med Care [Internet]. 2009;47(8):826–8. Available from: http://www.ncbi.nlm.nih.gov/pubmed/19584762%5Cnhttp://www.pubmedcentral.nih.gov/articlerender.fcgi?artid=PMC2728700 10.1097/MLR.0b013e31819a5acc PMC2728700 19584762

[5] Rodin G, MacKay J a., Zimmermann C, Mayer C, Howell D, Katz M, et al Clinician-patient communication: A systematic review. Support Care Cancer. 2009;17:627–44. 10.1007/s00520-009-0601-y 19259706

[6] Ong LM, de Haes JC, Hoos a M, Lammes FB, Hospital AM, Hospital AM. Doctor-patient communication: a review of the literature. Soc Sci Med [Internet]. 1995;40(7):903–18. Available from: http://www.ncbi.nlm.nih.gov/pubmed/18666041 10.1016/0277-9536(94)00155-m 7792630

[7] Van Dalen J. Communication skills in context: Trends and perspectives. Patient Educ Couns [Internet]. Elsevier Ireland Ltd; 2013;92(3):292–3. Available from: 10.1016/j.pec.2013.05.020 23810181

[8] Ha JF, Anat DS, Longnecker N. Doctor-Patient Communication: A Review. Ochsner J. 2010;38–43. 21603354 PMC3096184

[9] Stott NC, Davis RH. The exceptional potential in each primary care consultation. J R Coll Gen Pract [Internet]. 1979;29(201):201–5. Available from: http://www.pubmedcentral.nih.gov/articlerender.fcgi?artid=2159027&tool=pmcentrez&rendertype=abstract PMC2159027 448665

[10] Scott J, Revera Morales D, Mcritchie A, Riviello R, Smink D, Yule S. Non-technical skills and health care provision in low- and middle-income countries: A systematic review. Med Educ. 2016;50(4):441–55. 10.1111/medu.12939 26995483

[11] Schouten BC, Meeuwesen L. Cultural differences in medical communication: A review of the literature. Patient Educ Couns. 2006;64(1–3):21–34. 10.1016/j.pec.2005.11.014 16427760

[12] Williams M V., Davis T, Parker RM, Weiss BD. The role of health literacy in patient-physician communication. Fam Med. 2002;34(5):383–9. 12038721

[13] McWhinney IR. Why we need a new clinical method. Scand J Prim Health Care. 1993;11(1):3–4. 8484077 10.3109/02813439308994894

[14] Kitson A, Marshall A, Bassett K, Zeitz K. What are the core elements of patient-centred care? A narrative review and synthesis of the literature from health policy, medicine and nursing. Journal of Advanced Nursing. 2013 p. 4–15. 10.1111/j.1365-2648.2012.06064.x 22709336

[15] Jewkes R, Abrahams N, Mvo Z. Why do nurses abuse patients? Soc Sci Med [Internet]. 1998;47(11):1781–95. Available from: papers2://publication/uuid/CFC6FF8A-AE20-41E4-A3FE-4244A7A5FF2F 10.1016/s0277-9536(98)00240-8 9877348

[16] Manojlovich M, DeCicco B. Healthy Work Environments, Nurse-Physician Communication, and Patients’ Outcomes. Am J Crit Care. 2007;16(6):536–43. 17962497

[17] National Institute of Statistics of Rwanda (NISR). Statistical Yearbook, 2016 edition (SYB2016) [Internet]. 2016. http://www.statistics.gov.rw/publication/statistical-yearbook-2016

[18] Binagwaho A, Kyamanywa P, Farmer PE, Nuthulaganti T, Umubyeyi B, Nyemazi JP, et al The Human Resources for Health Program in Rwanda—A New Partnership. N Eng J Med. 2013;369:2054–9. 10.1056/NEJMsr1302176 24256385

[19] Timmermans S, Tavory I. Theory Construction in Qualitative Research: From Grounded Theory to Abductive Analysis. Sociol Theory [Internet]. 2012;30(3):167–86. Available from: http://stx.sagepub.com/lookup/doi/10.1177/0735275112457914

[20] Ritchie J, Lewis J. Qualitative Research Practice: A Guide for Social Science Students and Researchers [Internet]. Qualitative Research. SAGE Publications; 2003 356 p. http://books.google.co.uk/books?id=e6EO83ZKGYoC

[21] Cheng BSS, Bridges SM, Yiu CKY, McGrath CP. A review of communication models and frameworks in a healthcare context. Dent Update. 2015;42(2):185–186,189–190,193 10.12968/denu.2015.42.2.185 26058232

[22] Bylund CL, Peterson EB, Cameron KA. A practitioner’s guide to interpersonal communication theory: An overview and exploration of selected theories [Internet]. Patient Education and Counseling. Elsevier Ireland Ltd; 2012 p. 261–7. 10.1016/j.pec.2011.10.006 PMC3297682 22112396

[23] De Haes H, Bensing J. Patient Education and Counseling Endpoints in medical communication research, proposing a framework of functions and outcomes. 2009;74:287–94. 10.1016/j.pec.2008.12.006 19150197

[24] Schiavo R. Health communication: From theory to practice. Jossey-Bass; 2007 436-Chapter xxiv, 436 Pages p.

[25] Franzosi R. Narrative Analysis, or Why (and How) Sociologists Should Be Interested in Narrative. Annu Rev Sociol [Internet]. 1998;24(1):517–54. Available from: http://search.ebscohost.com/login.aspx?direct=true&db=aph&AN=1056952&site=ehost-live

[26] Courtney R, Rice C. Investigation of nurse practitioner-patient interactions: using the Nurse Practitioner Rating Form. Nurse Pr. 1997;22(2):46–8, 54–7, 60 passim. 9055316

[27] King A, Hoppe RB. “Best practice” for patient-centered communication: a narrative review. J Grad Med Educ [Internet]. 2013;5(3):385–93. Available from: http://www.ncbi.nlm.nih.gov/pubmed/24404300%5Cnhttp://www.ncbi.nlm.nih.gov/pubmed/24404300 10.4300/JGME-D-13-00072.1 PMC3771166 24404300

[28] Borrell-Carrió F, Suchman AL, Epstein RM. The Biopsychosocial Model 25 Years Later: Principles, Practice, and Scientific Inquiry. Ann Fam Med. 2004;2(6):576–82. 10.1370/afm.245 15576544 PMC1466742

[29] Flinders DJ, Noddings N, Thornton SJ. The null curriculum: Its theoretical basis and practical implications. Curric Inq [Internet]. 1986;16(1):33–42. Available from: http://www.jstor.org/stable/1179551

[30] Ogden J, Bavalia K, Bull M, Frankum S, Goldie C, Gosslau M, et al “I want more time with my doctor”: A quantitative study of time and the consultation. Fam Pract. 2004;21(5):479–83. 10.1093/fampra/cmh502 15367468

[31] Dugdale DC, Epstein R, Pantilat SZ. Time and the patient-physician relationship. J Gen Intern Med [Internet]. 1999;14(S1):S34–40. Available from: http://link.springer.com/10.1046/j.1525-1497.1999.00263.x 10.1046/j.1525-1497.1999.00263.x PMC1496869 9933493

[32] Marvel M, E RM, Flowers K, B HB. Soliciting the patient’s agenda: Have we improved? JAMA [Internet]. 1999 1 20;281(3):283–7. Available from: 10.1001/jama.281.3.283 9918487

[33] Snowden A, Marland G. No decision about me without me: Concordance operationalised. J Clin Nurs. 2013;22(9–10):1353–60. 10.1111/j.1365-2702.2012.04337.x 23121664

[34] Hain D, Dunn DJ, Tappen RM. Patient-provider partnership in a memory disorder center. J Am Acad Nurse Pract. 2011;23(7):351–6. 10.1111/j.1745-7599.2011.00619.x 21696483

[35] Makoul G, Clayman ML. An integrative model of shared decision making in medical encounters. Patient Educ Couns. 2006;60(3):301–12. 10.1016/j.pec.2005.06.010 16051459

[36] Kapiriri L, Bondy SJ. Health practitioners’ and health planners’ information needs and seeking behavior for decision making in Uganda. Int J Med Inform. 2006;75(10–11):714–21. 10.1016/j.ijmedinf.2005.10.003 16343988

[37] Dutta MJ, Basu A. Culture, communication, and health A guiding framework. Routledge Communication Series: Routledge Handbook of Health Communication [Internet]. 2nd ed Taylor & Francis; 2011 https://www.statsbiblioteket.dk/au/#/search?query=recordID%3A%22summon_FETCH-credo_entries_187584753%22

[38] Buchanan A. Medical Paternalism. Philos Public Aff [Internet]. Wiley; 1978;7(4):370–90. Available from: http://www.jstor.org/stable/2264963 11664929

[39] Carcone AI, Naar-King S, Brogan KE, Albrecht T, Barton E, Foster T, et al Provider communication behaviors that predict motivation to change in black adolescents with obesity. J Dev Behav Pediatr [Internet]. 2013;34(8):599–608. Available from: http://www.ncbi.nlm.nih.gov/pmc/articles/PMC4184411/pdf/nihms518766.pdf 10.1097/DBP.0b013e3182a67daf PMC4184411 24131883

[40] Sandman L, Munthe C. Shared decision making, paternalism and patient choice. Heal Care Anal. 2010;18(1):60–84. 10.1007/s10728-008-0108-6 19184444

