# Supplementary material for: Providers’ perceptions of communication with patients in primary healthcare in Rwanda
Source: PLoS One. 2018 Apr 4;13(4):e0195269. doi: 10.1371/journal.pone.0195269 (PMC5884556; doi:10.1371/journal.pone.0195269)
Supplement: S1 Dataset — (ZIP) [file pone.0195269.s001.zip › S1 Dataset/PPC-Provider 6.docx]

**PPC-Provider 6**

I: Interviewer; R : Respondent

**I:** I would like to remind you as I told you, everything we are going to talk now is about the conversation you have with patients in the consultation room, so you should direct the conversation to that. I would like to start asking this: What can you tell us about the conversation the patient have with the nurse in the consultation room at the Health Center?

**R:** It is the problem related to his/her sickness you talk about in the consultation room, he/she can also talk about what caused of his/her illness.

**I:** Eeh. What is the importance of the conversation between patient and nurse has on the work you do in the consultation room at the health center?

**R:** It has the importance in knowing the sickness the patient has, knowing diagnosis, treatment, giving medication, and helping the patient.

**I:** Can you tell us in detail how you understand or you see the good conversation between the patient and the nurse?

**R:** The good conversation is that, no matter what when the patient come to the hospital he/she seeking for treatment, he/she may have a normal illness which can be treated by medications, he/she may also be having sickness which does not need medications, rather need conversation only. The good conversation is listening to what the patient says, and explaining to him/her well depending on what he/she says so that they can have importance on him/her.

**I:** Uuh. Is it important for the nurses to have enough knowledge related to having conversation with patients?

**R:** Uuh, it is important to have enough information mostly because the patient come asking you the questions. He/she can ask you the questions about the sickness he/she has, you need to have enough knowledge in order to be able to explain to him/her.

**I:** Why do you think you need to have a good conversation with the patients who come to you?

**R:** I need to have a good conversation with the patients in order to have the origin of the sickness they have. Because if you don’t have a good conversation with him/her by listening well the problem he/she has, what caused the illness you cannot know what you can do for him/her.

**I:** Do you think the good conversation you have with patient can help in improving the healthcare or improving the way you look after the patients?

**R:** Yes, the conversation i have with patients can improve all that, because when you take time to listen to someone and sees that you are listening to him/he, he/she find it important.

**I:** Uuh, how? Can you explain more?

**R:** Please ask the question again.

**I:** I was saying that, do you think the conversation you have with the patient can improve the way you look after the patients?

**R:** Eeh, I said that when you give attention to someone it shows that you care for him/her which shows that you don’t value other things than him/her.

**I:** Uuh. What is the advantages of having good conversation with patients?

**R:** There are many advantages, on my side I am satisfied of helping someone, because after having a conversation and feel satisfied on his /her side, and also goes home having the result he/she was waiting for.

**I:** Do you think that having enough knowledge about having the conversation with the patients, can help you more in helping the patients who come to you?

**R:** Uuh. Having enough knowledge is important, that is why the ministry of health organise training on new things or strengthening the knowledge on a particular thing. That knowledge is important, and it should be available.

**I:** Uuuh, there you spoke about the knowledge of the nurse in general, the training you are given in general but here i am focusing on the knowledge about having the conversation with patients.

**R:** Yes

**I:** Here i am specific on the knowledge about having the conversation with patients. Do you think having knowledge about conversation with patients can be helpful in helping the patients who come to you?

**R:** Yes

**I:** How?

**R:** It can help because when someone have learnt something specific, like if I learnt counselling in general I can learn having conversation with patient only so that I can know how to direct the talk in the conversation I have with the patient. **I:** Uuh, can that be helpful in helping the patient who comes to you? Yes, it can be helpful in helping the patients who come to me.

**I:** Uuuh, in which way?

**R:** It is the one i was talking about, when you have learnt on specific thing you get to know how to follow certain steps in order to do things well based on the knowledge received**. I:** Uuuh. For example if I learnt certain thing, if I learnt malaria on a pregnant woman aside, in that case I will have more knowledge about malaria on a pregnant woman. It is the same, if there is a lesson or training which can be available about having conversation with patients can be helpful.

**I:** Uuuh. Have you had training or lessons which help you improving the way you have conversation with patients who come to you?

**R:** No, those training didn’t happen, we received counselling on different things, like patients who live with HIV, Counselling on Mental Health, those enable me to assess other patients, but i can’t say that i received training about having conversation with patients on its own.

**I:** Uuuh, ok. Do you find it important to be trained or learning about having conversation with patients?

**R:** It can be important**. I:** Why? It can be important since I focused on one side of the patients, and I have learnt it in general, if I am leading the conversation on those patients if I am trained on the conversation I have with patients and the way I conduct it, I think it can be important.

**I:** Uuh. On the measure of 1 out of 10, how many marks can you give to yourself on the knowledge you have about having conversation to the patients in the right way?

**R:** 1 out of 10? **I:** Uuh. I can give myself 7**. I:** You understand is not 10 out of 10, it is understandable that there is a gap somewhere. Uuh.

**I:** Where do you find a gap in relation to the knowledge you have which makes you not having conversation with the patients in the right way?

**R:** The gap? **I:** Where do you have the gap in the knowledge you have. Is it about my knowledge? **I:** Eeeh which makes you not having a good conversation with the patients who come to you? I can’t total say that the way I talk to them is not right, but if there is to be learn in order to help you having conversation with patients**. I:** Uuuh. Since I don’t have that knowledge or training, I think is the one which prevent it from being 10 out of 10 but no matter what I also try my best. **I:** Uuh. I do help them in the knowledge I have.

**I:** Uuh. Is there an area you can point, and say that in this area I have a gap of having knowledge about having conversation with the patients in the right way?

**R:** I cannot point immediately and say that in this area I have a gap, because in the conversation I have with patient there is a beginning and an end. **I:** Uuh. It also depends on the patient, and how he/she wants to be open or not to the thing you are talking about.

**I:** Uuh. What do you need to be improved?

**R:** What do I need to be improved? “laughing” **I:** Uuh, in order to have 10/10? “laughing” in order to have 10/10 if there is conversation regarding a certain thing, I think when a person is giving himself marks it requires someone else to give it to him/her**. I:** Uuh. Then, when the person is the one giving marks it requires the other person to give it to him/her. **I:** Uuh. If the person is giving marks, is when you can know that the person has weakness on this or that. What I can say is that training is always needed.

**I:** Uuh. How does the relationship you have with other nurses makes the conversation you have with patients go well?

**R:** My relationship with nurses? **I:** Uuh. If there is something I cannot explain well to the patient or don’t have knowledge about, I can ask another person so that I can explain well to the patient the problem he/she has.

**I:** Do you think it is important to ask patients if there is something specific they are waiting for which make them come seeking for treatment?

**R:** Eeh, there is a time the patient come and have a conversation with him/her but feel that he is not satisfied I do ask this: what can I do for you ? In that case when you ask, he/she immediately tells you what you can do for him/her. **I:** Uuh. It is also important to ask. **I:** Why do you think it is important? It is important because if someone comes to you asking questions and you don’t give him/her a good answer, he/she doesn’t go back home feeling satisfied. When you have a conversation and he/she is not satisfied, you can look at him/her and sees that, then ask what he/she was expecting from you? Then he/she tells you that.

**I:** Uuh. Is there something you do in order to know that the patient who come to you wants to know more information about his/her health status?

**R:** Those are my responsibilities. When I find out that the patient I am having conversation with is not understanding what I am telling him/her, I try to explain about his/her illness. Because when he/she is having the conversation with the patient you can know that he/she satisfied with this, she understand this or he/she doesn’t understand this.

**I:** Uuh. Then why do you do that?

**R:** I do that so that he/she can know for example about the sickness which can kill him/her, or try to prevent consequences caused by illness before they develop by giving advices.

**I:** Uuh. Do you think it is important to explain to patient everything he/she needs to know?

**R:** Eeh, what concerns about him/her. You take the patient as someone who comes having illness, it is important to explain everything related to his/her sickness.

**I:** Uuh. Why do you think it is important to explain to the patient everything he/she needs to know?

**R:** If it is something related to him/her, in case he/she has a particular sickness and needs to know everything related to that illness I do explain to him/her. It is important to explain so that he/she can know about it, and also know how to behave during sickness.

**I:** Uuh. Is it important to make patients who come to you, participate in making decision related to the way they wish to treated?

**I:** Uuh. **I:** Why? It is important because we don’t treat patients by force, you cannot also instruct him/her to do something you don’t understand. For example you can’t tell someone to adhere to ARVs if he/she doesn’t want it, it is not possible. He/she can refuse to take it and become lost to follow up but when you explain to him/her well, he/she gets to understand that once he/she takes it well, he/she will get better and continue his/her daily activities and in that case he/she does it by understanding what he/she is doing and also have contribution during their treatment.

**I:** Uuh. When the patient has to make choices about the treatment, should the nurse give value to that?

**R:** There is a time the nurse should give value to that **I:** When? There are some choices the nurse consider. There is a time the patient choose something due to lack of understanding about his/her sickness, and you find out that his/her choices are not profitable to him/her then you have to explain to him/her so that he/she can get understanding without imposing him/her. You have to explain to him/her very well till he/she gets to understand that what you are telling him/her is more profitable.

**I:** When the nurse is showing his/her emotions, how does the emotions contribute to the conversation he/she has with patient?

**R:** Do you mean emotions towards patient illness? **I:** Yes it is possible. When you are having a conversation with the patient, the patient can have certain emotions. Do you understand what I mean about emotions? Uuh. **I:** When you show them like a nurse, how does it contribute or influence the conversation you have with the patient? The emotions can bring out two things, you can have emotions and the patient keeps quiet, you can also have emotions and the patient tells you everything even the ones which were hidden. Then what does the question asks about? **I:** how does the emotions influence the conversation you have with the patient? It can influences due to those reasons, you can have emotions and makes him/her talk or keep quiet. To me I think those are influences. **I:** Can you give me examples? “laugh” it is not easy to find an example but we are not allowed to have emotions towards patients, sometimes you don’t need to have emotions you just have to keep quiet and listen to the patient, when you try to have emotions you may make the patient keep quiet and leaves without knowing anything. The same way he/she can feel free to talk. Unless you give me an example so that I can clarify what I said!

**I:** “laughing” is it acceptable for the nurses to show joy or sadness while they are with patient**? R:** It is acceptable. It depends on the case, it does not mean that we have to show all that at the same time but it depends on complicated cases or there is a time the patient will be open about his/her social life or talk about the improvement about his/her life since you started being together. There is a time you follow a patient, in that case if you show that you are happy it is not a problem. There is also a time you have to be neutral when the patient has a serious case which may even end his/her life it is not good to show sadness. **I:** Why not? In that case you are taking him/her in other mood, patients believe in nurses that he/she is someone who can treat the patient no matter the situation, and sometimes he/she has those thoughts in him/her. So when you show that you are not of doing something, he/she feels that his/her life has come to an end. That’s how I understand it, he/she can feel dead**. I:** Uuh. Do you mean that it can make him feel hopeless? Yes they can make him/her hopeless.

**I:** Do you think patients can have challenges about talking to nurses about problems related to their health?

**R:** For me I think they don’t have them, unless if is the patient we know each other from where we stay. Even the one you know each other in the area where we stay can trust you and tells you his/her social problems. But I am thinking that if I meet a patient without knowing each other from somewhere, there is no problem of telling me his/her social problems.

**I:** Uuh. Why do you think that the patients cannot have those concerns?

**R:** Most of the time patients trust nurses and feel that they can get solutions to the problems they have from them.

**I:** Uuh. How is it having conversation with the patient with low education background? For example like patients who doesn’t know how to read and write?

**R:** Patients who don’t know how to read and write in the conversation related to sickness? **I:** How do you find it? It is not about those who doesn’t know how to write only but everyone in the domains. He may have a problem and explain to him/her about sickness and he/she may not understand it because he/she doesn’t know the cause or may ask me something and find out that he/she doesn’t know anything in that area.

**I:** Uuh. How do you use the knowledge you have in having conversation with people or patients in that stage?

**R:** Those patients are complicated when you have to explain to them something which is not familiar **I:** Things which are how? Things which are not familiar, things which goes deep in medical term. In that way you try to explain in the way he/she can understand it. **I:** How do you do it? “laugh” you can’t write for someone who doesn’t know how to read and write, it is understandable that you can’t read for him/her but if he/she comes and tells you that he/she is suffering from kidney and says that it starts by having swollen eyes, if he/she tells you that you don’t have to accept that it is started by having swollen eyes because you know the symptoms of the illness, rather you try to explain and make him/her understand that the symptom given is not among kidney symptoms.

**I:** Do you feel Rwandan culture can contribute to the conversation the patient have with nurse?

**R:** It depends on the sickness the person has, like those related to the culture. **I:** Explain more please? You mean Rwandan culture? **I:** How does Rwandan culture contribute to the conversation the patient have with nurse? You mean making him/her healed or being of the importance to him/her? **I:** I mean any contribution or influence Rwandan culture can have in the conversation between you and the patients? In Rwandan culture, there are things required to express respect in general. When the patient find out that the nurse has respect Rwandan culture require us to have, it helps because he/she sees that you have respect toward him/her and becomes easy to express about him/herself.

**I:** Uuh. Based on how you see, what makes the conversation not go well to patient side due to the patient? **R:** The conversation does not go well due to the patient? **I:** Uuh. The conversation may not go well due to the patient or due to lack of knowledge and resistance, and he/she does not understand what you explain to him/her that is the example I can give or he/she doesn’t want to talk or tell us something.

**I:** Uuh, are there other things which you think can make the conversation not go well because of the patient?

**R:** The patient are the cause? **I:** Uuh. It is because the patient does not trust the nurse who is going to treat him/her or have certain mind about him/her, when he/she is also going to treat him/her, he/she doesn’t have trust towards him/her. When the patient doesn’t have trust for you, things does not go well.

**I:** Based on how you see, what are the reasons which mostly cause the conversation not go well on the nurse’s side?

**R:** On nurse’s side things may not go well due to small based on the time the nurse has for the patient. If you have a small time for the patient, he/she also sees that and decide to be reserved. **I:** Uuh. Are there other reasons which can make the conversation not go well due to the nurse? Maybe knowledge, if the nurse does not have enough knowledge that can also be a problem but nurses mostly face the challenges of time, because nurses experience makes you understand things but time to give to the patient may be small depending on the number of the patient you need to receive.

**I:** What are the reasons that make the conversation not go well because of the function of health center?

**R:** On the functioning of the health center? **I:** Uuh. Here I don’t know what to say! **I:** Uuh, its ok think about it. On health center side **I:** There is a way that the health center may function, and those things may make the conversation not go well? On my side I cannot say much on that because even if the health center has bad reputation outside, and the patient has managed to enter as a nurse you have to be useful to him/her. On that side unless it is a problem to the patient or the nurse? **I.** “Laugh”. Here is where you can say that the health center has bad functioning or bad leadership I don’t know. The nurse who work in that situation may not do his/her work well, even when the patient comes will be received depending on the nurse’s mood that’s what I can say, but normally the problem related to nurse’s good or bad behavior stopped there does not affect the patient.

**I:** Uuh. Can you give an example of things which are difficult to talk to the patients who come to you?

**R:** Difficult to talk about? **I:** Uuh. The patient who come to me have different problems, there is nothing I cannot talk to the patient, depending on what brought him/her, no matter what if the patient comes having a particular situation which brought him/her and have certain beliefs. What can makes it difficult is his/her belief in order to explain to him/her, but there is nothing difficult which can prevent me from being useful to him, that’s what I think. **I:** Uuh, I am talking mostly about thing you can say that, this can be difficult of talking to the patient. It depends on the category, giving the patient sad information may be difficult but no matter what he/she needs to know about it. You need to find ways of saying it so that he/she can know about it, because you can’t let him/her go without knowing about it. It is difficult to inform sad information but we have to do it. Uuh. Announcing death or telling someone that a particular person is dead is difficult, informing prenatal death. Mostly we are not the ones who announce that, we tell that them that the baby is having a problem of breathing and transfer them to the hospital to do echography then inform her that the baby is no longer breathing. It is also difficult to inform someone that he is HIV Positive and he/she is going to live with it all his/her life but that does not prevent us from saying it because they need to know how to behave.

**I:** Uuh. Then on those examples you gave, in case you have to announce to the patient when he/she is having serious sickness, a particular problem or sickness you think is not going to be cured. How do you behave in that situation?

**R:** In that case, a person remains neutral and know about his/her sickness then give the patient a chance to react, but no matter what you need to inform him/her.

**I:** Uuh. Have you ever met a patient who was challenging to talk to at work because of a particular problem?

**R:** The problem he/she has? **I:** Uuh. Yes **I:** What was the problem? He/she was having stomach-aches, and thinking that if he/she is having stomach-aches and think that he/she doesn’t have to do normal activities. If a person has that problem and feel that he is always sick while stomach problem does not prevent a person from working. A person can have stomach crises today and don’t have it after tomorrow. Making him understanding that he/she has to work while he/she feeling sick is a problem, but at the end he/she understands it. **I:** How did you behave in that situation? I worked on it slow by slow, and took me time to talk and make him/her understand, but at the end he/she understood that he/she needs to work even though he/she is sick.

**I:** Have you ever met a patient who was difficult to talk to at work because he/she was having mental health problems?

**R:** No, we don’t meet them often.

**I:** Have you ever met a patient who was difficult to talk to because of his/her character or disabilities such listening, talking or others?

**R:** Not listening? **I:** Not talking, blindness and other things like that? I didn’t meet someone who is blind, also dump people I met are not many and when I meet them I use sign and he/she also has someone who interpret for him/her. In that case he/she is the one who speak, and also explain to him/her.

**I:** Have you ever met a patient who was difficult to talk to because of the difficult personality of the patient?

**R:** No **I:** Here I am talking about character, hard personality? No **I:** you didn’t meet anyone? Uuh.

**I:** If it happens that you meet him/her, how can you behave in that situation?

**R:** I can try my best and if I find it challenging, I ask support to a colleague. I can try to have a conversation with him/her or explain what brought him/her and if I find out that he/she doesn’t make an effort to understand, I try to find my colleague who can help.

**I:** Is it necessary to talk to the patient about a particular problem or sickness you think he/she has?

**R:** When he/she doesn’t know about it? **I:** Of course he/she doesn’t know about it! He/she doesn’t know about it, it’s sickness discovered just like that? **I:** Uuh. It is necessary. **I:** Why is it important? Because he/she needs to know about his/her sickness so that he/she can participate in treatment process.

**I:** What can you tell the patient, in case you are not able to know well the problem he/she has?

**R:** In case I can’t know well the problem the patient has, I can’t stop him/her on my level in health center, I just explain and also tell him/her that I didn’t manage to know the problem you have due to this reasons. After telling him/her that I also say that we do meet these cases in the health centers but you can get more support in the hospital. For example I can’t tell the patient that I didn’t find the sickness while I know that she has sickness which can be known after doing x-ray checkup, I can’t stop him/her on my level, I do explain to send him/her for check-up. About the medications prescribed by nurses, it is important to explain to the patient the type of medication prescribed, how he/she should take the medication, medications side effect, and how the medication work.

**I:** Eeh, it is important. **I:** Why is it important? “laugh” when we give patient medications which makes him/her sleep, we tell him/her that this medication treat this. If he/she has allergy we say that this is anti-allergy or sedatives which cause much sleeping. If is someone who drives a car, we tell him/her not to drive while taking the medications. It is important to tell him/her that so that he/she doesn’t get medications side effect and cause other problems. So it is important.

**I:** Some of the patient in Rwanda think that they don’t receive enough information about medications. Based on your experience or what you see, is it like that?

**R:** What i see is that, they don’t even want to ask information. The nurse talk to them about medications, and find out that they don’t even need to know about the medications because they mostly think that what is important is the medication. Even though they say that they are given few information, you find out that they are not interested of knowing more information than what was given. That is how I see it. There is an example, they like buying medications in the pharmacy, sometimes he/she come and prescribe amoxicillin, next time when he get the cough for a particular period and prescribe amoxicillin , next time if he/she is having the cough or his/her child he/she will buy that medication while the nurse who prescribed the medication explained the medication use, side effect or cure, and he/she goes and buy it thinking that he/she has all information even when goes there, he/she doesn’t even ask information before getting medications, he/she doesn’t need to add more information on few ones received in order to develop the knowledge.

**I:** Uuh. What can the nurse do in that situation?

**R:** If he/she finds out that the problem is there? **I:** Uuh. Myself as a nurse I do what need to be done. I have explained the medication use, and function.

**I:** Uuh. What cause that?

**R:** What make them say that they don’t receive information? **I:** Can in it be like that or as you are saying patient are not interested of knowing more much or knowing more information given by nurses? It depends on patients categories. Like those you said that they don’t know how to read, or write even the ones who are just there waiting to be helped and leave without any contribution. Sometimes you explain things and he/she doesn’t understand well but when an intellectual person comes and explain about the medication he/she understand it.

**I:** Does your system function or the situation you work in prevent having a good conversation between you and the patient?

**R:** It is the situation of many patients who are waiting one nurse, just that I don’t have precise number, but there are number of patient a person need to treat per day and give everyone certain minutes. I think the only obstacles to the conversation is time**. I:** How? If I have to give you medication in 20 or 30 minutes and give you 10 or 15 minutes it is the half of the minutes I should give you. Those type of functioning are the ones which can be an obstacle to the conversation. **I:** In which way? If I have to receive the patient in 30 minutes, in those 30 minutes we can talk all things need to be spoken, then I give him/her 5 minutes because they are other patients who are waiting for me, in that case I will not give him/her enough time, he/she will also speak less because of the time I have.

**I:** Uuh. How do you handle the situation of the patient who wants to be transferred to the hospital while you think it is not important?

**R:** It depends on the patient. There is a patient who listen to the information given, myself I can give him/her time. I can tell him/her that I can see that you want to meet the doctor but it not yet time, go and use the medication given if you find out that is not helping you, please come back and see what we can do. He/she can accept that but there are also the ones who insist. On my side i haven’t met the ones who insist.

**I:** What can you do in case you meet the one who insist?

**R:** If I meet the one who insist, I would allow him/her go and meet the doctor.

**I:** Uuh. Are there problems you see related to having conversation in Kinyarwanda?

**R:** Most of the time finding Kinyarwanda words for medical terms it’s challenging. It’s just trying.

**I:** Uuh. Learning in French or English is a challenge because you have to speak to the patient in Kinyarwanda?

**R:** The fact that I have learnt in French or English, there are some terms I learnt in English and French and it is challenging to translate them in Kinyarwanda, but I try and find them. I don’t think that can be the reason I don’t explain to the patient well, even though it is challenging I have to find Kinyarwanda words to you use. They are working on Kinyarwanda, now we can find words to use**. I:** Uuh. They are still working on it, they haven’t found words if I am not mistaken? They are doing their best working on it, there are Kinyarwanda terms they are getting which help us explain to the patients.

**I:** What do you do when you have to explain medical terms which are in French or English which does not have Kinyarwanda terms?

**R:** I don’t meet those cases much, but you can draw in order to make the person understand things.

**I:** Drawing how?

**R:** We draw by showing the patient a certain sickness which attacked a certain area of the body and is in French which cannot easily find Kinyarwanda terms to use. You draw by saying for example that this is river, kidney and they are like this and this and explain where the illness has attacked by showing that and in that case he/she can know that the kidney has attacked this part of the body according to the body structure. He/she understands. **I:** Can you give us example of where something happened and found it difficult to explain in Kinyarwanda till you start drawing? “laugh” do you see nephropathy, it attacks nephron. By telling someone nephropathy you draw the kidney by showing those parts, after drawing those parties by showing them to the patient by explaining where the illness attacks and got to understand where the sickness attacks.

**I:** Are there other challenges you experience by having a conversation with patients who come to you we didn’t talk about in what we discussed about?

**R:** I think, we have none.

**I:** What can be done to improve the knowledge you have in having conversation with patients?

**R:** Improved? **I:** Uuh. Unless there are particular lessons related to having conversation with patients only. **I:** Uuh, the conversation with patients? Is that what you said? I was talking about the way the nurse talk to the patient, what can be done so that the way you have the conversation with the patients can be improved? I am talking about trainings, I learnt something but training also is needed.

**I:** Patients are different, which means you even have different ways of having conversation. How do you use your knowledge in different ways patients use in having conversation?

**R:** Is relating them with the problem they brought to me or have in order to have a conversation with them. **I:** How do you do that? They are different, and bring different problems. The way they are different, they bring different problems. In that case when the patient come and tells me a particular problem, I look at the knowledge I have and explain his/her problem.

**I:** Uuh. What can be done so that the nurse can help the patient have a better conversation in the consultation room?

**R:** During the consultation? **I:** Uuh. I know that I have to receive the patient well during consultation, I do see him/her by the time he/she comes the same way they see me also. When you are prepared and receive him/her well or not he/she sees that. The way you receive him/her that’s what make you having a good conversation with him/her.

**I:** Uuh. How do you handle the situation where the patient cry?

**R:** When the patient cry you allow him/her cry, because he/she is having something which is making him/her cry and after crying you ask what is making him/her crying. For example he/she sometimes comes and start crying without telling you anything, there is also another time he/she cries because of the result received about his/her health status. In this case you need to allow him/her and cry.

**I:** Is it necessary to support the patients who come to you, by dealing with emotions caused by the sickness they have?

**R:** Uuh. It is necessary. **I:** How? The same way the patient have this sickness and think that it can kill him/her soon, you need to help him/her by showing examples of others who have that sickness or how others accept their sickness. No matter what, you need to find out what to tell him/her so that he/she can feel well and also understand that his/her emotions don’t have to make him/her worried and end his life immediately. **I:** How important is that? They are important, so that he/she can live with his/her life well and also control his/her emotions, and also understand that those normal situations.

**I:** Is there something you do to make sure that the patient understands well what you are saying?

**R:** Uuh. **I:** What do you do? You ask this: do you understand this well? He/she can tell you that he/she understands or not. **I:** Why do you ask? Is because he/she doesn’t also understand.

**I:** The nurse should help the patients participate in the treatment they receive.

**R:** Uuh. **I:** How can he/she do that? The nurse should support and contribute to the treatment the patients receive. Some of the sickness are caused by bad behavior so we don’t need to wait till they get sick, rather we educate him/her in order to be prevented from those sickness or if he/she got the sickness once, he/she doesn’t have to get it at the second time. You have to educate him/her by telling him/her that even though he/she treated, he/she doesn’t have to give opportunity to the sickness to come again.

**I:** Is there something else you would like us to talk about on what we said?

**R:** « laugh » you ask too much! These questions are too much.

**I:** We are about to end.

**R:** These questions are too much! I don’t even know their purpose, if there are about knowing the conversation, what is their purpose? I think you didn’t explain that to me? These questions are too much, they make you tired.

**I:** When we finish there is no problem we will explain to you. Do you think they are other questions we should ask which will help in having a good conversation between patient and nurse?

**R:** I think there is none, you asked many questions.

**I:** Uuh. [*Name*], thank you very much

**R:** « *laugh* »
