# Supplementary material for: Providers’ perceptions of communication with patients in primary healthcare in Rwanda
Source: PLoS One. 2018 Apr 4;13(4):e0195269. doi: 10.1371/journal.pone.0195269 (PMC5884556; doi:10.1371/journal.pone.0195269)
Supplement: S1 Dataset — (ZIP) [file pone.0195269.s001.zip › S1 Dataset/PPC-Provider 7.docx]

**PPC-Provider 7**

I: Interviewer, R: Respondent

**I:** Well, the reason is that, you see, I cannot keep in my head all the answers you will give me for all the questions nor can I write all of the answers because you…[*Interrupted*]

**R:** I was thinking that you writes the answers somewhere?

I: You write…[Interrupted]

***R:*** I thought that you give someone a piece of paper and they answer the questions on the paper?

I: Aha, no. We do it orally. It’s not a questionnaire, it’s an interview. We do it orally. That means, it helps us to recall what you said even when we are not here. This is a qualitative study, so we analyze what you say; we are not asking you to fill in the questionnaire. There are people who do quantitative study and see how many people said something. This is a qualitative study. Is there a problem?

**R:** No problem.

**I:** Okay, thank you. So, before we start, I would like to remind you that everything that we will discuss is related to the conversation that you have with patients when you are in the consultation room. Now, I would like to begin by asking you, “Can you tell us a little bit about the conversation that a healthcare provider has with a patient in the consultation room at the health center?”

**R:** Concerning the conversation that a healthcare provider has with a patient in the consultation room, when a patient enters into the consultation room, you greet the patient, give them a welcome and they sit down. After that, you introduce yourselves to each other. You tell them your name as the healthcare provider who is going to treat them, and then the patient also tells you their name. So, you proceed with the conversation. You tell them that what you are going to discuss will remain secret between them and you. You keep talking and then you ask them about the problem that they have; I think it’s that.

**I:** Okay. What is the contribution of the conversation that a healthcare provider has with patients to your work in the consultation room at the health center?

**R:** On the side of the healthcare provider, the contribution is… [Interrupted]

I: So, contribution in general?

*R:* When a patient comes to see you, there are so many things they need from you. They need to recover from their illness. Then, when you are conversing with them and spend some time together and talk about various things*,* there is a possibility that – actually the conversation is not only about illness – it can also be about their life. So, the patient can recover from the illness and resume their everyday life.

**I:** Tell us in full details what you think a good conversation between a healthcare provider and the patient is like?

**R:** A good conversation between the healthcare provider and the patient is the one that happens in a place where you are the only people who are there. The place should be calm and secured in such a way that no-one else can suddenly open the door and interrupt you. In that case you can converse smoothly and the patient can tell you everything that they feel they have to say and as a result you get all the information you need from them thanks to the fact that you are alone.

**I:** Is it necessary that healthcare providers have enough skills in conversing with patients?

**R:** Huh, it is necessary. It is necessary because if you aren’t able to successfully engage someone in conversation they cannot feel open to you and tell you about their illness. Therefore, it is necessary to know how to proceed so that you understand their illness.

**I:** Why do you need to have a good conversation with patients?

**R:** Huh, as I said it multiple times, it is essential to talk to a patient in a kind way because if you have been able to talk to someone in a friendly way, I think the friendly conversation is already half the cure of their illness. Therefore, when the conversation has been going well, everything else goes well too. When you give them medications, they go home saying, “There is hope of recovery because what the healthcare provider has done for me is good.”

**I:** Do you think that having a good conversation with a patient can help you to improve the way you care for patients?

**R:** Huh, as a saying goes, “A good word entails an equally good word from the listener;” Rwandans say this, don’t they? So, this means that when you have had a friendly talk with someone, everything goes smoothly.

**I:** Now, in a detailed way, try to explain to me how a friendly conversation can help you to improve the way you care for patients?

**R:** Huh. For example someone can come to seek healthcare and then you give them medications but it may happen that when they arrive at home they won’t be able to find something to take along with the medications, for instance good food or drinks that help the body. So, when you have had a good conversation, you get to know all those issues that exist at their home and you know that they won’t be able to get food to eat while on the mediations; you know about all those issues and you help them accordingly.

**I:** What are the benefits of the friendly conversation that you have with patients?

**R:** The benefits of the conversation that one has with patients include the fact that you can advocate for them. You can advocate for them thanks to the conversation that you have had. As I said, someone can come to seek healthcare for malaria but you also see that - for instance you can see that a child has got malaria but maybe they also have malnutrition. Thanks to the friendly conversation that you have had, the child may be experiencing problems at home that have led to their malnutrition. In that case you can advocate for them so that they receive support in terms of fighting against malnutrition. Additionally, a good conversation can enable the healthcare provider to effectively help the patient. I can give you an example of cases that we usually encounter with. Someone may come to seek healthcare for malaria on multiple times and when they come, you just examine and give medications and finish by there. But when you have been able to go deep and ask for more information, you can be able to know if the patient has got a mosquito net at home, if there is a problem where they live. When you have had a good conversation you can give them advice, and as the health care provider you have the opportunity to help them definitively so they no longer have malaria every now and again.

**I:** Do you think that having enough skills in talking to patients can enable you to help patients in a better way?

**R:** Huh.

**I:** How?

**R:** It can help you; because after you get the sills, you get to know how you can behave, what you can do if a patient reacts in a certain way. You can know how to talk to patients step by step. For me I think it is necessary.

**I:** Did you receive any courses or training that can help you improve the conversation that you have with patients?

**R:** I didn’t receive any training at all. Maybe when it comes to courses, it’s just the ones that we get at school.

**I:** Do you think that it is necessary?

**R:** To learn about that?

**I:** Huh.

R: It is necessary. It would be helpful because they can even teach you how you can hold the conversation even if a patient answers you in a weird way and things like that. They can also teach you where to start the conversation and where to finish it. I think it is necessary.

**I:** Huh, what importance does it have?

**R:** There is importance. It I useful for me as the healthcare provider because it can help me to know bow to start a conversation with a patient. Then, you understand that a patient benefits from the fact that they are being treated by a healthcare provider who knows how to talk to them.

**I:** On a scale of one to ten points, how would you rate your skills in conversing with patients in a suitable way?

**R:** Out of ten I would give myself six points.

**I:** Okay, in any case you understand that you lack four points; because, according to the way I understood it, there is a gap. Can you tell me where the gap is so that, once filled in, you would able to talk to patients in a suitable way?

**R:** Where the gap is, as I said, I think there should be “guiding steps” that one should use when talking to patients. I think that’s where I have a problem.

**I:** By ‘steps” you want to mean ‘methodologies”, is that right?

**R:** The way you can proceed with the conversation when talking to a patient.

**I:** Explain that in detailed way?

**R:** In detailed way, for example when someone comes you can start by greeting them, give them welcome, and then ask them how they are feeling. They can answer you that they are doing well and finish by there and say nothing else. So, it would be helpful to know how you can progress with the conversation and go deep into details to know more information when the patient is reluctant to talk. You should know other techniques you can use to subtly drag enough information out of the patient.

**I:** What do you feel you need to improve?

**R:** Concerning the conversation that one has with a patient?

**I:** Huh, so that you score ten points out of ten? Maybe you can say ‘I need to improve such and such skills in my communication with patients”?

**R:** What I need to improve, I would like to know an individual very well.

**I:** How? Explain to me well?

**R:** I think what I need to know is how to talk to any type of patient who comes to see me. Because people are different and each person has their own culture. So, what I need is how I can talk to each type of patient as an individual.

**I:** I don’t know if I understand well what you say, but are you trying to tell me that what you need is to know how to carry on a conversation with people who have different problems?

**R:** Huh, you should know how to handle them.

**I:** How does your collaboration with other healthcare providers influence positively the conversation that you have with patients?

**R:** My collaboration with other healthcare providers? How it can play a role?

**I:** How does your collaboration with other healthcare providers positively influence your conversation with patients?

**R:** It’s good that we have a common goal, and work together. For instance, there are educations that we give to patients before we start treating them. So, when we collaborate well and organize the education very well, it helps community members who come to see us. Because we teach them before we treat them. In this way, if my colleague teaches them something and that I have a conversation with them after, I do not struggle because my colleague has preceded me to talk to the patient.

**I:** Can you relate this to the consultation?

**R:** If we relate it to consultation, you see for example that malaria has risen up, cases of malaria have risen. So, if a patient comes to seek healthcare for malaria and finds that we have taught people about malaria on that day, I do not encounter with any difficulty because I already have a starting point. The conversation happens after my colleague has taught them what malaria is and then I go from there when we are in the consultation room.

**I:** Do you think that it is important to ask patients if they came to seek healthcare expecting something particular?

**R:** Yes, I think it is important.

**I:** In which way?

**R:** It is important because a patient can come to seek healthcare and say “Let me just try and go even if I don’t know if they will help me”; but I think it would be helpful for them.

**I:** Asking the patient if they expect something from their visit to the health facility, how does that help them?

**R:** It helps them to recover. Because sometimes someone falls sick and stay at home. But when they come to the health facility and that you have a conversation with them and give them advice, they realize that it was necessary that they seek healthcare.

**I:** Is there something that you do to know if the patient needs more information about their health?

**R:** I cannot say that it’s for all patients, but there are some patients who come and you see that they really need to know more information about their illness depending on their illness.

**I:** What type of patients are they for example?

**R:** Like whom? Like patients who, let me see, like patients who have mental problems.

**I:** What do you do for them?

**R:** It’s an example I am giving you; you can advise them to go to health facilities which are more advanced than the health center.

**I:** Why do you do that?

**R:** I do that so they can get more advice. Because we are at the lowest level, the health center; so we refer them to hospitals where there are people who offer high level medical care.

**I:** Do you think that it is necessary to explain to patients everything that they would like to know?

**R:** It’s necessary, it’s their right.

**I:** Why is it necessary?

**R:** It’s necessary. You should not do for a patient anything that they do not want. You should know everything they would like to know: if they wish to have information about the medications they have been given, they should get it. If they wish to have information about the tests that they have had, it’s their right to know about that.

**I:** What is the importance of that? How useful is this?

**R:** It is useful for them. It’s only because we are in countries which are not yet developed, but you can tell a patient that you are going to do a test for HIV/AIDS and they have right to refuse. If they refuse, you cannot do the test. Additionally, concerning medication, patients should know about the medications they are going to be given because if they do not understand that they have to take them, you can give them the medications but they refuse to use them and the whole thing becomes useless. But if you have discussed about the medication, they know about it and therefore they take it properly.

**I:** What you say triggers me to ask you the next question. Is it important to let patients be involved in making decisions related to their healthcare?

**R:** Huh, it’s important.

**I:** How?

***R:*** Repeat the question?

I: I had asked you if it is important to let patients play a role in making decisions related to the healthcare that they would like to be given.

**R:** Yes, it is important like I have said. It’s just what we have said; a patient is in a weak condition but they have right to their life. They are responsible for their life. So, in case you want to do for them anything that they dislike, you are ignoring their responsibility.

For example, the patient might be an Adventist, sorry a Jehovah’s Witness can tell you “I do not want any healthcare provider to give me blood transfusion. My church recommends that we don’t receive it”. So, when you have had a good conversation and that you know about it, there is no reason for giving them blood transfusion when their church does not allow them to receive it, and I think it is right. If you do that, you are going against their belief.

**I:** When a patient would like to make a choice regarding healthcare, should the healthcare provider take the patient’s choice into consideration?

**R:** You can give importance to it but also give them advice.

**I:** When a healthcare provider exhibits emotions or feeling, how does it affect the conversation that they are having with the patient?

**R:** It would be useful because the patient can see that the healthcare provider is feeling their pain. Suppose that the patient tells about something that made them very sad; you cannot cry but you can just show them that you are sorry for them.

**I:** Do you think this is important? How important is it?

**R:** It is very important because the patient realizes that you have understood them and that you have felt their sorrow. They might even feel so open that they can tell you other things that they wouldn’t tell you if they had realized that you are not sorry for them.

**I:** Is it acceptable that healthcare providers exhibit happiness or sadness when they are with patients? Take your time and think about it, no problem.

**R:** In my opinion, as the healthcare providers we are not allowed to cry when a patient cries, but we can still show that we are sorry for them. But you cannot cry. For instance if a mother comes to seek healthcare for her baby and that the dies shortly after arriving; she can burst into crying you cannot cry. No, you can just show her that you are sad about the death of her baby, but you cannot start crying.

**I:** Why is it necessary that you behave like that? I mean to show sadness or happiness? Why do you think it is acceptable?

**R:** As I said, it shows the patient that you are together. Because even you, if someone shows you that they are sad but you laugh, they will see that you are not sympathetic for them. But normally, they have to cry but you should not cry so that you are able to help them get out of their sorrow.

**I:** Do you think patients can be worried about telling their health related problems to healthcare providers?

**R:** Yes, it is possible.

**I:** If it is “yes”, sorry you said that it is possible. Then what can be done?

**R:** What can be done is having a good conversation that helps the patient to feel comfortable and then tell you about their problems. Because they cannot tell you anything unless they are comfortable with you.

**I:** What is your experience with patients whom you think they have low level of education, like those who are illiterate?

**R:** Challenge that one encounters with?

I: Huh [yes]

R: Challenges happen when you are explaining things to them. It is difficult to explain things to them. It requires long time to explain to them because, you know, a person who studied is not lie the one who didn’t study.

**I:** How do you apply your communication skills to talk to patients of that category that we are talking about?

**R:** Thanks to the knowledge that you have, you know how to handle illiterate people. You give them so much explanations because an illiterate person doesn’t know many things; they know that – it’s an example that I am going to give you – they know that malaria is caused by sugar canes. But someone who studied knows that malaria is caused by mosquito. In that case, they have different levels of knowledge but you try and help them.

**I:** Does the Rwandan culture play a role in the conversations that a patient has with the healthcare provider?

**R:** Huh.

**I:** How?

**R:** Basically, we abide by the culture in what we do. If someone believes in something because of their culture, we must let them their right. If someone dislikes something because of their culture, we must also let them their right because they are compelled to dislike it by their culture.

**I:** So, our culture, the Rwandan culture, how do you think it influences the conversation that you have with patients?

**R:** How the culture influences the conversations that I have with patients? I don’t see what one can say about it but I think one should respect the patient’s culture because it is also necessary. I do not have an idea, once I get an idea I will tell you.

**I:** Okay, no problem; let us continue. In your opinion, what are the factors that hinder the conversations? I mean the conversations between the healthcare provider and patients? But factors arising from the patient’s side?

**R:** Reasons that can hamper the conversations on the side of the patient?

I: Yes, on the side of the patient

R: It is the way a patient can behave. This can hamper the conversation. Also, the state in which the patient is in can also hamper the conversation.

**I:** Explain it in full details?

**R:** Conversations can go in a bad way because of the patient. There is a person who comes to seek healthcare being drunken. In that case, it is difficult to talk to a drunk person. Additionally, the patient can have a mental problem, or the patient can also be a dumb who cannot speak. This can also hamper the conversation. There are many reasons.

**I:** You say that there are many factors and I thought that there are many other factors you didn’t mention. Can you also say them?

**R:** People have different moods. Someone can come speaking to you angrily; because a patient does not start from the consultation when they seek healthcare. They may come after being bored because they have been waiting for so long and therefore they might arrive in front of you still feeling bored. So, when you start to talk to them, they do not immediately regain their normal mood, they still see the picture of everything that happened before they come to see you. That issue becomes a hindrance to the conversation that you are going to have.

**I:** Here you are talking about the time when a patient has been given a bad service before they come to see you. Now what are the reasons that usually hamper the conversation on the side of the healthcare provider?

**R:** There shouldn’t be any reason.

**I:** There shouldn’t be any reason, but there are. There are some even if you wish that there aren’t.

**R:** You can go out and someone speaks to you in a rude way and you get angry. Maybe within that a short period of time it can be a problem and you can be unable to help someone. But this shouldn’t happen. But we are all human beings, getting angry is inevitable.

**I:** If I got you well, do you want to talk about personal problems of the healthcare provider?

R: Huh [yes]

I: Like family problems or the fact that someone at work can drive you angry? I don’t know if that is what you are trying to mean?

**R:** Normally, family problems should not be brought at work; you should not show your anger to a patient if you have had a problem at home; it isn’t even good. No, it’s not good. What I was saying is for instance, if I have been in the consultation room and that I go outside and then I meet with someone who tells me something bad, I can get angry as a human being but I shouldn’t remain angry all the day long. However, of thirty patients that I will receive, I will not talk friendly to maybe one patient because of the bad feeling that I have; but it can subside and the conversation goes in normal way.

**I:** Apart from what you have just said, do you think there are other reasons that can hamper the conversations? Reasons coming from the healthcare provider’s side?

**R:** No other reason. I don’t think a patient should be victim.

I: Think deeply.

R: No reason. It’s only the reason that I have mentioned – the fact that a patient should not be victim of the healthcare provider’s problems. It can only happen temporarily if there that drives the healthcare provider angry and that they fail to get rid of their anger in immediate effect; but in any case there is no reason for that to happen. If you have other problems, you can ignore them.

**I:** What you are answering me is like a wish; a patient should not be victim but it can happen that they are victim?

**R:** They can be victim of that temporary anger. But if you have had an argument with your husband at home, you should not spend all the day angry in the consultation room. No, you try to leave all those issues over there at the main entrance. What I am trying to say is like you can go outside of the consultation room for example and then you meet with somebody and they tell you something that upsets you. Maybe shortly after that you can come back to the consultation room feeling angry, but the anger subsides slowly; I think it has to subside. Even if two patients out of thirty can be victim of that, others cannot be affected.

**I:** A while ago, you said that a healthcare provider is a human being. Aren’t there any factors that can come from the healthcare provider that can negatively affect the conversation with patients?

**R:** No factors. We correct any potential problems.

**I:** Which one do you correct? What?

**R:** There are no problems, there is nothing that can prevent the conversation from going well.

**I:** Now, on the side of the health center’s work conditions, what are the reasons that usually hamper the conversation between a healthcare provider and a patient?

R: Because of the work conditions?

I: Yes, the work conditions at the health center. What are the usual reasons why the conversation does not go well between the healthcare provider and the patient?

**R:** Huh, maybe it’s between us, employees. For instance we are in the middle of the conversation with the patient and suddenly my colleague opens the door and come and say “I need you.” In that case the patient will stop talking. Later on when you want to resume the conversation with the patient, you won’t remember where you stopped, you have difficulty remembering and then you restart the conversation once again. That is one factor. The other factor is general, it doesn’t happen here only. It is everywhere because of the big number of patients while healthcare providers are very few. You can have more than fifty patients and you have to see all of them alone. So, when you are talking with the patient, you try to say little in order to try and save time to see other patients who are outside.

**I:** Can you give me examples of things which are difficult to tell patients about?

**R**: Things that are difficult to discuss with patients?

I: Yes, things which are difficult to tell patients. The ones that can make you say “I think I may have difficulty explaining these things during the conversation in the consultation room!”

**R:** Because of one’s illness?

I: Because of any reason.

R: Difficult things are things which are related to infertility. Infertility for someone who is married, who has been infertile for a long time; that is one thing. Another thing is the fact that someone is very old and they don’t have relatives. In that case, you have difficulty talking to such people.

**I:** How? What do you do when you receive such people?

**R:** When you receive such a person and that you find it hard to talk to them….[interrupted]

**I:** I mean, it can happen that you find the subject you are going to discuss with them is hard for you, what do you do?

**R:** In that case I can look for my colleague to come and help me; I don’t deal with it on my own.

**I:** At any time during your work, did you ever receive a patient whom it was difficult to talk to because of their own problems?

**R:** Huh [yes]

**I:** What was the problem?

**R:** Let me see, you mean receiving the patient in the consultation service or in other services?

I: It’s only in the consultation room.

R: In consultation room only?

I: Huh.

**I:** I remember you told me that it is difficult to talk to old people who live alone, who don’t have children. Can you go from that example and think about other types of people whom it is difficult to talk to because they have certain problems? Think about those you received?

**R:** Among the ones I received I saw an old woman who had no child but she used to have children.

**I:** What did you do at that time?

**R:** It was difficult, but I managed to get help from my colleague, because he studied about “counseling”; he tried to counsel the patient and then I was able to talk to her.

**I:** Did you receive a patient with whom it was difficult to talk to because they had a mental illness?

**R:** Huh, I received one.

**I:** What did you do?

**R:** It is difficult but in that case you try to side with them. If they tell you, “I see something over there”, you also agree and say “yes, it’s true.” You try to agree with everything that they say. It is in this way that we managed to talk even if it was difficult.

**I:** You also touched upon people who do not hear, who cannot speak. There are also those who cannot see or who may have other disabilities. Did you receive a patient with whom it was difficult to talk to because of such disabilities?

**R:** Yes, the one I received was a dumb.

**I:** Or a deaf? How did you manage to talk to them?

**R:** You try and use signs, and they also use signs to show you what they mean.

**I:** Did you receive a blind patient?

**R:** No, I have not yet received a blind patient.

**I:** Did you receive a patient with whom it was difficult to talk to because they had a difficult personality?

**R:** Huh.

**I:** How was that?

**R:** They speak to you in a rude way; they are reluctant to answer you when you ask them something, it was in that way.

**I:** How did you handle that?

**R:** You put up them and continue the conversation.

I: Do you want a short break now?

R: No, there is no problem.

I: Can we continue?

R: Let’s continue.

**I:** Is it necessary to tell patients about a problem which you think they have?

**R:** Yes, it is necessary.

**I:** Why is it necessary?

**R:** It helps them to know how they have to behave. For example, if you see that they have diabetes, or hypertension, it is necessary that you discuss their problem with them so they know how they have to behave, because it will help them in their illness.

**I:** What can you tell a patient when you are unable to identify their problem?

**R:** For us who are at health center level, if you aren’t able to know a problem that a patient has, I don’t think you should keep the patient; you should rather refer them to higher health facility where they can find appropriate help. And this is what we do. They can explain to them more about their illness, or they can do further tests and be able to explain to them their illness.

**I:** Concerning medications that the healthcare provider prescribes, is it important that the healthcare provider explains to the patient the type of medication they prescribe for them, how it works and any potential side effects it may or not have and even how to use it?

**R:** It is important.

**I:** How is it important?

**R:** Medications can have what we call “unwanted effects.” If you have not explained those effects to the patient, they can go home and use the medication but when they experience those problems they can stop using it. As a result, instead of getting better, the patient’s condition becomes worse.

**I:** Some of Rwandan patients think that they do not receive enough information about the medications. Is that true as per your experience?

**R:** That’s true.

**I:** What is the reason behind that?

**R:** As I said, this problem is caused by the fact that there are many patients while there is a few healthcare providers. The healthcare provider has many patients to see and therefore they do not have enough time to give all necessary information to the patients.

**I:** Do some of the situation at work hamper the smooth conversation between you and patients?

R: Can you repeat the question?

I: Do some of the situations at work or do your work conditions hamper or can your work conditions be a challenge to the smooth conversation that you have with patients?

**R:** No, they can’t.

**I:** What do you do if a patient requests for a transfer to go to the hospital when you think it is not necessary?

**R:** It is very difficult because there are people who leave home [***I:*** *Speak up a little bit?*]. It is very difficult because there are people who leave their homes feeling that they must – for example, mostly community members feel that they have to go to [name] hospital to have an x-ray test but when you examine them, you see that it is not really necessary. So, thanks to the conversation you have with them and advice you give them; and as a healthcare provider you can use other methods to show them that it is not really necessary to go there. You show them how long it will take them and the expenses it will incur. You use all these factors to convince them that it is not necessary. However, when they insist that they need a transfer, you let them go because it is their right.

**I:** Do you have any challenges when you are talking about health related issues in Kinyarwanda?

R: Challenges?

I: Yes

R: Because of Kinyarwanda language?

I: Because you have to talk about health issues in Kinyarwanda. Do you have any challenges?

**R:** Yes, challenges are there.

**I:** Which ones?

**R:** It happens that someone comes to seek healthcare having an illness on their sexual organs. So, when you ask them, “Where is the problem?” they seem to be ashamed of saying where they have a problem. They feel ashamed of saying it in Kinyarwanda. If it is a person who studied, they tell you in French.

**I:** The fact that you studied in French or in English, is it so challenging for you that you aren’t able to talk to patients in a suitable way?

**R:** No, it is not a challenge for me.

**I:** You don’t think there are challenges?

**R:** No challenges.

**I:** What do you do when you have to explain to patients English terms or French terms that are used in healthcare system but they don’t have equivalents in Kinyarwanda?

**R:** What one does?

I: What do you do?

R: It is difficult. You don’t have any other option, so you are forced to speak in the language that you know.

**I:** In the language that you know, can you explain more? Those terms to not have equivalents in Kinyarwanda.

**R:** Well, you just try to paraphrase and manage to show them what you mean, but you still use the original language in which the term is. In that case, for instance when you are talking about ‘hernia” the patient knows that they have it, so you try to tell them like, “This illness is like this, these are its signs”.

**I:** Can you elaborate on this point?

**R:** For instance a patient can come complaining that they have a problem of hernia, for example they may have a swelling and feel pains. In that case you tell them that hernia is the illness that they have.

**I**: Do you encounter with any other problems that we haven’t touched so far?

**R:** Challenges that one encounters with?

**I:** Yes, the ones you encounter with when you are talking with patients who come to see you; but I mean the problems which we have not discussed?

**R:** Yes, there are.

**I:** Can you explain in more details?

**R:** There can come a male patient and find that there is a female healthcare provider in the consultation room whereas they would rather talk to another healthcare provider of the same gender; therefore the patient feels uncomfortable. Similarly, there can come a woman patient and when she finds that it is a male healthcare provider who is in the consultation room, she abstains herself from talking, she feels uncomfortable.

**I:** Can that affect the conversation that you have with the patient?

**R:** yes, it can.

**I:** In which way?

**R:** In that case, if the patient is female and that she finds a male healthcare provider, she will feel less open to say everything; she will say just some things and skip other things. Therefore, the conversation will not be good and as a result you cannot reach your goal.

**I:** What can be done to improve your skills in conversing with patients?

**R:** I should go to study. When one studies, one learns new things that they didn’t know. Additionally, it would be helpful if there was training about that.

**I:** Patients are different. How do you apply your communication skills to converse with different communication styles of patients?

**R:** The way one talks to adult people is not the way one talks to young people thanks to the skills that one has got. If a young person comes from outside and says to you ‘Thumps up.” It’s true that you have to do that so you can build rapport with them. But if it is an old person, you will also know how to talk to them.

**I:** What can be done so that the healthcare provider helps patients to talk more comfortably to them during the consultation?

**R:** It would be better if the healthcare provider would close firmly the door so that patients feels that they are the only two people in the room.

**I:** Anything else? What else can the healthcare provider do to help the patient talk to them more openly?

**R:** Also, the healthcare provider should try to remain kind so that the patient feels comfortable enough to talk to them.

**I:** But here I can ask you: it is possible that the healthcare provider shows to the patient that he/she is a very superior person; does that happen sometimes?

**R:** Yeah, it happens.

I: So, do you think it is a problem that spoils the conversation that a patient has with the healthcare provider?

R: Yes, it is a barrier because once a person is not comfortable to talk with you, they feel that you are very highly important and therefore they cannot tell you anything.

**I:** What do you do when a patient cries?

**R:** When a patient cries, you let them cry first and after they have finished crying, you start to talk to them.

**I:** Is it useful to help patients manage their emotions resulting from their illness?

**R:** Huh, it is very useful.

**I:** How is it useful?

**R:** I think it is useful, especially psychologically.

I: How?

R: If the patient has lost hope thinking that they will not live longer in the future, you can help them psychologically and this can help them and this can prolong their life.

**I:** Is there anything that you do to ensure that patients understand what you are saying? What do you do?

R: You can tell them something and ask them to repeat it after a while. In that case, you can evaluate and see if they have understood what you agreed on.

**I:** Why do you do that?

**R:** It is good. It helps you know if the patient has kept something about what you discussed. For example when it comes to the schedule of taking medications, if they have to take three types of medications, you can explain to them how they will use them. So, it is good if they can repeat the schedule for you; you get to know if they have understood because sometimes they repeat and you understand that what they repeat is different from what you told them.

**I:** Should a healthcare provider help patients to be involved in the healthcare that they are given?

**R:** It is good that the healthcare provider helps patients to play that role.

**I:** How should they do that? What should they do?

**R:** In my opinion the healthcare provider is like an advocate for the patients. They can prescribe medications for a patient but unfortunately they patient can find that the medications are not in the stock, so the healthcare provider can help the patient by advocating for them so they can get those medications. Maybe if the medications are not in the stock today, they can do their best to find them the next day so that the patient gets the medications.

**I:** I don’t know if you understood the question correctly, but if we come back to the consultation room, because this is where we are focusing on; should a healthcare provider help patients to participate in the healthcare that they are given?

**R:** Yes, the healthcare provider must help patients.

**I:** How can he do that?

**R:** For example in the consultation room you can receive a patient in April and you treat them for a given illness. Then, the same patient returns in May for the same illness and you notice that they didn’t respond to treatment since they did not get any better; you can help them so that they can go somewhere there is advanced healthcare so they can recover from the illness.

**I:** So, in that case, how does the patient participate in the healthcare that they are given?

**R:** The patient participate when their illness did not respond to the medication and then they return. But there are patients who can stay at home when their illness did not respond to treatment.

**I:** Why should the healthcare provider help patients participate in the healthcare that they are given?

**R:** It’s because it is the patient’s right to participate in the healthcare which they are given.

**I:** Do you have anything to add on the conversation that we have had?

**R:** What I can add is that, as we have said it, the conversation between the healthcare provider and the patient is very important. What I can say is that it would be good if the healthcare provider would receive only as few patients as he will be able to have enough conversation with. He should not just say little to patients thinking that there are many patients outside waiting. The problem is that it is impossible, but it would be better for patients to have a conversation with the healthcare provider because it is essential.

**I:** Do you think there are other questions that we should ask that would contribute to the improvement of the conversation between patients and the healthcare provider?

**R:** I don’t think there are other questions you should ask. Because you have asked me many questions, I cannot remember what you asked and what you didn’t ask.

**I:** [*Name*] thank you so much!

**R:** Thank you! [*Both interviewer and respondent laugh so much as they finish the conversation*]
