# Supplementary material for: Providers’ perceptions of communication with patients in primary healthcare in Rwanda
Source: PLoS One. 2018 Apr 4;13(4):e0195269. doi: 10.1371/journal.pone.0195269 (PMC5884556; doi:10.1371/journal.pone.0195269)
Supplement: S1 Dataset — (ZIP) [file pone.0195269.s001.zip › S1 Dataset/PPC-Provider 1.docx]

**PPC-Provider 1**

I: Interviewer, R: Respondent

**I:** So we are now going to start our conversation; and as we start, I would like to ask you “ Can you tell us a little bit about the conversation that a patient has with the health care provider in the consultation room at the health center?”

**R:** *Speechless moment*.

**I:** Huh, tell us in general

**R:** For example when a patient meets me in the consultation room?

**I:** Huh, can you tell us about the conversation that you have?

**R:** Usually when a patient comes in the consultation room, they arrive when we already have their address. Over there at the reception service, patients’ addresses are recorded; so when a patient comes in they usually sit down and then I ask them what they have come to see health care for. They explain to me what they have come to seek health care for and then I understand it and either I prescribe medications for them or I recommend tests for them and they go to have the tests at the laboratory. If they need just medications only, I prescribe them and they go and get them at the pharmacy.

**I:** Thank you. In your work in the consultation room, what is the importance of the conversation between the patient and the health care provider?

**R:** The importance of the conversation is that when a patient talks to you, you are able to go deep into details and find out more about their illness. Although a patient comes to seek health care for a specific problem, but you may uncover other reasons that made them come to seek health care. You may discover another illness that might be the root cause of what they came to see health care for.

**I:** Tell us in full details what you think about the best conversation between a health care provider and a patient?

**R:** The best conversation happens when a patient comes and then they feel comfortable and then you have a conversation and the health care provider doesn’t speak to the patient in a rude way, he gives them a good reception; that’s when the patient feels free and then they talk about their problems. The patient can even go out of the subject a little bit and tell you about their family. Usually when you have a good conversation with a patient, when you have received them in a good way, they feel free to talk to you and it is really useful because you get to discover more about their problems.

**I:** Is it important that health care providers have enough knowledge concerning how to converse with patients?

**R:** Yes, it is very important [**I:** Why is it important?] because when you have a good conversation with someone, it’s only then that you are able to know more about their problem. Someone may come to seek health care but if they notice that you have not engaged them in conversation in a good way, they can hide their illness and they may even return home without having sought health care saying “How can I tell my problems to that damned health care provider who doesn’t know how to talk to me?” So, that is why when you have had a good conversation, they feel free and tell you everything. There are those who come to seek health care for headache, they come and tell you “I have headache” and then you go deep in the conversation and you ask them more questions and you come to find out that they also have…they usually hide illnesses that affect sexual organs. So, depending on how you have had the conversation, they can tell you about it whereas they had feared to tell you about it before.

**I:** Do you think that the conversation that you have with patients can help you to improve the way you care for the patients?

**R:** Yes, very much indeed. [**I:** How*?*] Of course you understand that if you have had a good conversation and that they feel free, they tell you the illness which they were likely to hide from you. You see that the conversation is useful. But if you do not talk to them well enough, they can go back home without having sought health care, and you see that the conversation becomes useless *[I: And what is the importance of the way in which you care for them?]* When you have had a good conversation, you understand that when you have had a good conversation with someone, you care for them better. For example there may come a patient who is rude to you and you lose your temper and you feel that you cannot care for them and you wish that they go out immediately; because there are some patients who are rude.

**I:** Okay. Do you think that having enough knowledge in engaging patients in conversation can help you to improve the way you care for patients who come to see you?

**R:** Of course yes! [**I:** How?] Well, you understand that when the healthcare provider…it’s very true compared to giving medications. What was the question? [**I:** *I had asked you if enough knowledge can help you to improve the care that you give to patients who come to see you.]* Yes, because you see, if you get more knowledge concerning engaging patients in conversation you will be able to discover more about their illness which they probably wouldn’t tell you. And you also understand that if you know more, it will be useful. If you have a long conversation, they can tell you many things concerning their life in the community, and from that story you can for example find out that it is an illness that is ravaging the whole community. So, that is also very important.

**I:** Did you ever receive training that was aimed at helping you to improve the way that you talk to patients?

**R:** In fact we had a training about counseling, I don’t know if it is the same as engaging patients in conversation [**I:** *Communication skills*?] No, I haven’t had a training about it yet. [I: Is it necessary to have that training?] Yes, of course. It would be useful. [**I:** How*?*] You see, when you have conversed with someone, there are a lot of things that you know about them. In the same respect, if you have a good conversation with these patients who come to see us and you talk about their illnesses, there are a lot of things that you can discover thanks to your ability to converse with them.

**I:** And then on a scale of 1 to 10 points, how prepared are you to converse with patients who come to see you in an effective way?

**R:** I am prepared at least at 5 per cent [**I:** Five out of ten!] Yes, sorry, five out of ten. [**I:** *So, where do you have a gap that prevents you from effectively engaging patients in a conversation? Because you understand that you still need another five points?]* The gap is mostly caused by the large number of patients that we receive. I work in the consultation service, but on daily basis, it is very rare that I receive less than 70 patients per day. So, you understand that it is very difficult to have enough time to converse with a patient when you see many others on the queue waiting to see you. That is a very big problem that impedes your possibility to effectively engage patients in conversation. [**I:** *But do you think that there is a gap on the side of your knowledge or your skills in conversing with patients?]* No, I don’t have any gap related to the conversation with patients because I have a lot of knowledge; even if I didn’t have training about it but I studied about it at school, I had a course about it. The problem is mostly due to lack of time. But conversing with patients is not a problem for me.

**I:** Okay. How does your collaboration with your colleagues make the conversation that you have with patients better?

**R:** Collaboration of what …? [**I:** Your collaboration with other health care providers, your colleagues, how does it impact the conversation that you have with patients and makes it better?] The impact that it may have is that when you have a good relationship with your colleagues, our patients can see it through the way we talk to one another. But if for instance a patient overhears you quarreling with your co-worker, they cannot feel comfortable when they come to see you; they may overhear your colleague and you arguing and shouting to each other and when they meet you into the consultation room or elsewhere and may say, “I am going to see the health care provider who speaks rudely, I am afraid, I don’t know what I will tell them!”

**I:** In what ways do you think that it is important to ask patients if they expect something in particular from their having come to seek health care?

**R:** Importance? [**I:** How or in what ways do you think that it is important to ask patients if they expect something special from the fact that they have come to seek health care?] That helps you to know the quality of your service. If they tell you that they expect good things, you can sit down and evaluate yourself and find that for instance you are giving a good service, but if you understand that they do not approve of your service, then you can get to discover that you have been giving a bad service and you therefore decide to make amendment accordingly.

**I:** Okay. Is there anything that you do in order to know when a patient needs to know more information?

**R:** What I do maybe…after service when we meet or…? [**I:** *Well, we are talking about your experience in your daily work, do you do something to know if patients who have come to see you need more information?]*More information about what we were… [ **I:** *Yes, what you were talking about or…]* Well, nothing except that they ask me questions on their own. Sometimes you can be with someone and you realize that they are trying to know many things and therefore they ask you for example the reason why….or they can ask you “What has caused my illness?” during the conversation, that’s how it happens.

**I:** Do you think that it is necessary to explain to the patient everything that they need to know?

**R:** Yes, it is very necessary. [**I:** *Why ?*] because if you explain to them for example how an illness is transmitted, if you tell them things which are incompatible with the medications, that can be useful; it helps them not to relapse any more. If they know how an illness can be transmitted to other people and also its causes, it is important. But when you do not explain to them, they can relapse anytime and they can return to see you and yet, the more patients return to the health facility, the more our work increases; if they do not come back, it is good because the health care provider has less work.

**I:** Is it important that patients participate in decision making concerning the care that they would like to be given?

**R:** Yes, it is also good but patients should make sure their decisions are good for them; if the health care provider finds that the decisions are not good, they should correct them in such away that they agree on them. For example a patient may want to be given a certain type of medication and you see that it is not convenient for them. However, if a patient prefers a type of medication which you think it cannot have side effects, you can let them have it and then they go happily. But if you think that the medication can cause problems for them, you can deny them the medication. But if a patient wants to make their own decision and that I see that it cannot cause any problem, I don’t mind. [**I:** Why do you think that it is good?] Come again? [**I:** Why do you think that it is good that they get involved in making decisions*?*] It helps them to…when they take medications which they are happy for – well, actually recovery depends also on hope. For me if a patient comes in the consultation room and that I see that their suggestion is similar to something that I was planning do for them, I don’t even argue with them; I do as they wish. [**I:** *So, if a patient has a preference as you are saying in the example that you gave us, should a health care provider consider it?]* Yes, it cannot cause any problem, you can consider it.

**I:** When a health care provider shows their emotions, how do the emotions influence the conversation that they have with the patient?

**R:** Someone may want to try to care for a patient and you see that they are doing for them what is not being done for other patients or you can see that someone is taking a patient from other patients in order to care for them and maybe you want to do for them something which they did not come for. That’s when, I think, you can see that the health care provider is showing their emotions.

**I:** So, is it acceptable that they show their happiness or sadness when they are with a patient?

**R:** It is not acceptable! [ **I:** *Why is it not acceptable?*] From my perspective, you should not show your happiness to a patient. The patient may be surprised when you have happiness and therefore they say, “Hey, is the health care provider happy always? Is it the first time that they feel this feeling?” and they may not consider it as good or they may lose trust for you and consequently think that you don’t have enough skills in what you are going to do for them. You had said happiness and what? [**I:** *And sadness*] And sadness, it is not good to feel very sad either. You can feel sad and make the patient feel that their condition is very serious and therefore they feel that their illness has become complicated because they see that the health care provider is very sad and then they say, “Oh, it’s not easy!” For me the best thing is that you should disguise your happiness and your sadness; you should show the normal mood all the time. *[****I:*** *If I got you well, it means showing your feelings can have negative consequences to the patient?]* Yes, it is possible: you can see how serious their problem is and then you have feel sadness and as a result the patient feels that it’s dangerous and feel that their illness has gone to a complicated stage. But if you remain in your normal mood even if the patient’s illness is serious, they cannot feel hopeless.

**I:** Do you think that patients can be worried about sharing their health problems with health care providers?

**R:** Yes, they can be worried. [**I:** So, what can be done?] What can be done is that health care providers should show patients that they are together, and they should feel free and show that they have unity. But it happens that health care providers make themselves very important people to the extent that patients feel afraid when they want to see them. In that case, you understand that the patient cannot be comfortable with the health care provider. It requires that they both feel equal and have good conversation, and therefore the patient can feel free even before they meet with the health care provider, feeling that they are just going to converse with their friend.

**I:** What can the health care provider do?

**R:** What they can do, the health care providers should try and talk to patients even outside the work, they should show them that they are equal and avoid to show them that they are very important people. At work, if the health care provider passes patients somewhere, they should talk to them that they are not just an important person. [**I:** For example when they are having a conversation in the consultation room?] No, there is a way that your face can show it. Sometimes when you are conversing with someone, you can show them that you are actively listening to them and that you are a simple person and you don’t scare them, therefore they feel open to you.

**I:** What is your experience with conversing with patients who you think they have low level of education, for instance those who didn’t study how to read and write?

**R:** Those patients with low level of education feel very afraid of you. Therefore you need to make them feel free by showing them that you are at the same level, you show them that even if you studied, but they also have value, in that way they can feel open to you.

**I:** I want also to ask you how you use your knowledge to talk to patients who are in that category.

**R:** We try and guess their category. For instance if it is an illiterate patient who comes from the village, you converse with them in a way that is related to their category. Also, you may receive a patient who is smart – you immediately guess they studied or that they are rich – so you start to converse according to their category. So you know how to talk to them depending on their category.

**I:** How does the Rwandan culture influences the conversations that patients have with health care providers?

**R:** You try to comply with the culture of the place where you work; if you are talking to a patient you should not bring in things that happen in other countries where you once lived or travelled to; you just concentrate on things related to that specific area; you should not go beyond and tell them things that they do not know about. During the conversation, if you want for example to give them an example, you use an example of something that happens in their community, you use for example Rwandan things and if you are talking about cleanliness you try to show them… I mean what they see there, you should not tell them things that they cannot understand but when it comes to the culture, I do understand but I don’t know how I can explain it to you.

**I:** I can help you and put the question in another way. You see the Rwandan culture, our culture as Rwandans, does it have an impact on the conversations that you have with patients?

**R:** Yes.

**I:** So, how does our culture as Rwandans, have an impact on the conversations that you have with patients? [**R:** How shall I explain it to you?] You know, there are many things in the Rwanda culture. Rwandans have their way of speaking, they have their way of behavior and so forth, in short you know that a culture comprises a lot of things. Those things that make a culture, do they have an impact on the conversation that you have with patients?

**R:** It may have an impact maybe through the examples that you give them mostly. If you are for example telling a patient how they must behave – in the former time, old men had a culture of remarrying their sons’ wives after their sons died; so if you are teaching them about sexually terminated diseases you can tell them that this culture has disappeared and things like those related to the new culture that we are adopting.

**I:** In your opinion, what are the factors that usually make the conversations bad on the side of the patient?

**R:** Bad conversation on the side of the patient? [***I:*** *Huh. What makes the conversations bad, but on the side of the patient?]*On the side of the patient, I think it is usually dependent upon the way they feel about you as the health care provider. That is when they may opt to not talk to you, they can in short refuse to converse with you. Because the patient comes to see you, they are usually willing to tell you about their problems. The way they feel about you is what can make them stop telling you anything, I think the most reason may be the way they have felt about you the health care provider. Some patients might refuse to talk to you but as you keep asking and asking, they end up being open to you especially because they know you want to help them.

**I:** Huh. From your experience, what are the reasons why conversations are usually bad on the side of the health care provider?

**R:** For the health care there are many factors involved. A heath care provider may be tired having a lot of work and feeling tired can cause that they do not talk to the patient effectively. There are others who have a bad mood and who do not know how to converse with people or who are not kind enough to converse with people. But most of the time you see that it is tiredness and a lot of work that usually cause that.

**I:** What are the factors connected with the health center’s organization that make the conversations better?

**R:** There is leadership and also the relationship among co-workers because when they have a bad relationship, things do not go well too. If there are conflicts among some employees, in short when employees do not have a good relationship, many things are negatively affected. A team that do not collaborate is not good.

**I:** Can you give us some examples of things which are difficult to tell to patients?

**R:** Difficult things? [**I:** *Difficult to tell patients*….] If patients are not so many, I don’t think there would be something difficult. It is difficult only if you see that you have many patients who want to see you or if you have a lot of work. You can be there working in the consultation service thinking that you will soon go to dress wounds, I will go to vaccinate children. Shortage of time or having a lot of work are the things that can make it difficult for you to talk to patients but if you have enough time, I don’t think there is a problem there. [**I:** *Is there something that you think is difficult for you to tell patients? Is there one?*] For me I don’t think there is something.

**I:** Okay. In your daily work, did you ever receive a patient whom it was difficult to talk to because of their mental illness?

**R:** Yes. I received such patients.

**I:** Or was there any other problem apart from the one I have just told you? Was there any other thing that made it difficult for you to talk to the patient?

**R:** Yes. There are people who come to seek health care when they have other family issues and therefore you try hard to talk to them but they are unwilling to talk to you because of the problems that they have at home. We usually receive such patients.

**I:** Did you ever receive a patient whom it was difficult to talk to because of their natural condition like deafness, dumbness, blindness?

**R:** We usually receive such patients. They come to seek health care but they come with their care givers. For instance a dumb person who comes with a caregiver, the caregiver speaks in the place of the patient.

**I:** Did you receive a patient whom it was difficult to talk to because of their character?

**R:** Yes, there are. Young men for example are harsh in a certain way and when you ask them something you see that they are unwilling to answer. We usually receive them. Also, we receive drunken people. We also receive those cases. They are so drunken that you think they have fought with other people in pubs and you feel that they may not allow you to talk to them. We also receive those ones.

**I:** You usually receive them. You once gave an example and you said that sometimes someone comes to seek health care having also other family problems, or like those young boys whose character is difficult. What do you do in such situations?

**R:** You try and keep your temper because if he gets angry you cannot be angry too. You may feel a slight anger in your heart but you try and disguise it. Because both of you cannot get angry, otherwise you cannot achieve anything. If he speaks to you angrily, you try and speak kindly and sometimes they talk to you in a humble way in the end.

**I:** Is it necessary to tell a patient about an illness which you think they have?

**R:** Huh, it’s good. [**I:** Why is it good?] You cannot hide it from them whereas you want them to try and seek health care for it. Because if you tell them about it, you also show them the consequences ahead, that’s why they decide to go and seek health care. But if you ever didn’t tell them about it, you would not have helped them at all.

**I:** What can you tell a patient when you are not able to identify their real problem?

**R:** You tell them that you are not able to understand their illness but you ensure them that you are going to refer them to people who can do more than you are able to do.

**I:** Concerning medications that the health care provider prescribes, is it important for the health care provider to explain to the patient the type of the medication that they prescribe?

**R:** Yes, it is important. You tell them the type of medication that you prescribe for them, how to use it and the illness for which it is. For me I think that it is important because the patient takes the medication knowing what it is treating them for. But you can give them a bunch of three or four types of medications without explaining to them what they are and they may even throw them away saying, “What are these damned medications that they gave me?” Sometimes you can take medications for two days and you start to feel well, in that case the patient may throw them. But if you have explained to them for example “These medications have to be taken for two or five days” they cannot throw them away even if they may feel better after two days. If you have told them “The medications are effective if taken for X number of days otherwise the illness does not get cured completely” so they may keep taking the medications until they finish them. But if you don’t explain that to them, they can even go and immediately throw the medications. Those are the examples that we usually see.

**I:** So, it is necessary to explain to the patient about the medications?

*R: Yes it’s very important.*

*I: The type of medication, how it works, how it is used and any side effects that is may have?*

R: Yes

**I:** Some patients in Rwanda think that they do not get enough information about the medications. Is that true as per your experience?

**R:** That’s true.

**I:** In your opinion, what is the reason behind that?

**R:** That’s what I was telling you, here we receive more than one hundred and fifty patients. Let us suppose that we have three consultations in the morning and patients leave the consultation heading to the laboratory. We give patients their tests results at about eleven or twelve o’clock, and all of them go to the pharmacy. At the pharmacy, there is only one pharmacist – it is rare that there are two pharmacists – and when patients arrive at the pharmacist’s, she is maybe tired. So, what she does is to just write “One pill in the morning, one pill at noon and one pill at evening”, this is an example. She cannot afford to explain everything to the patient. Also, it is difficult to explain everything to the patients when you are in the consultation room. Since patients outside keep complaining “They are delaying”, you cannot take time to explain “These are three types of medication, this one is for this and that one is for this, their side effects are these.” If you do that, you can take ten minutes while other patients complain about the delay of service. In fact those are the challenges that we usually encounter with, but if there were a few patients, you would take time and explain to them without any problem.

**I:** Does any of your working conditions impede the effective conversation between you and the patients?

**R:** For our working conditions…

**I:** Yes, some of your work conditions that can hamper the conversation between the patients and you?]

R: Repeat it again so I understand it?

I: Okay, do some of your work conditions prevent the good conversation between the patients and you from happening?

*R:* Yes, it is possible.

**I:** *Can you explain more*?]

R: Well, you may not have… I mean you there should be enough infrastructure in order to effectively converse with patients. Sometimes you are not given the equipment, sometimes you don’t have time because of work load and I think this also can have an impact. On top of that, you may not have a place where you can talk with patients, this also can impede the conversation with the patient.

**I:** The place where you can talk with patients?

R: Yes, the place may be inconvenient because of the room’s placement. Maybe the environment where the room is or the rooms are not available*.*

**I:** Okay. What do you do when a patient requests to be referred to the hospital while you think that it is not necessary?

**R:** Yeah, they sometimes request that but you think it is really not necessary. For that one, you try to explain it to them and make them understand. On the other hand, some patients ask you for a transfer when you are not also able to figure out what their illness is or when you are not sure that you will definitely cure their illness; in that case I let them go. But a patient may ask me for a transfer it is not possible, so I explain to them and show them that their illness is not so serious and I ensure them that I will cure them at any cost. You can tell them “Go and take these medications for five or seven days first of all and come back here if you do not get cured, I will refer you there you want to go.” In fact, it all depends on how you discuss. Yes, you do not immediately allow them to go. However, if a patient asks you for a transfer when you also see that it is necessary, you let them go.

**I:** Do you think there are any problems related to the conversation about health in Kinyarwanda?

**R:** Is it difficult?

**I:** Do you see any problems related to conversation about health in Kinyarwanda language?

R: Aha, there are some terms which you don’t have equivalent in Kinyarwanda because of how we learnt them. But in such a situation you try and explain them and maybe if you don’t have words to use, you try to paraphrase it until you make them understand the terms, but it really happens.

**I:** When you said like that, you made me think of another question for you. The fact that you were taught in French or in English, is it a challenge when it comes to conversing with patients in Kinyarwanda?

**R:** Yes, there are challenges.

I: Can you explain more?

R: Well, there is an illness which you cannot easily explain in Kinyarwanda. In that case, for instance if it is *ascaris,* you tell them how it is called in French and you try and tell them that it is a tapeworm. It is a type of stomach worms which is caused by lack of hygiene. That is the example I can give you.

**I:** Huh. Are there other challenges related to the conversation with patients that we didn’t discuss?

**R:** I think we have discussed many of them.

**I:** Okay. What can be done to improve your knowledge in engaging patients in conversation?

**R:** Maybe, even if one thinks that he/she knows how to converse with patients, if one had a training whereby one would be shown the true way of conversing with patients, maybe they would gain some more knowledge in that subject. Because for me, I would say that I had a chance to learn things like those and even if one does not remember well what one studied at school, if they organized a training after sometime, that would probably be useful.

**I:** Patients that you receive are different. How do you match your knowledge with different communication styles of patients?

**R:** Normally, you have to examine the personality of the patient. You usually go where the patient leads you to, but you have to be careful; if they lead you to bad things, you avoid going in deep details. You go there, but you try to return them on the right path where you see it is necessary.

**I:** What can be done so that the health care provider helps patients to have a better conversation with him/her during consultation?

**R:** The first thing I do is giving patients a good reception. You have to receive them well, telling them good words, in a kind way and showing them that you are together and that you are happy for them; that is what can build the foundation of a good conversation between the health care provider and the patient. That is the first thing: receiving a patient well clears the way for other things.

**I:** What do you do when a patient cries?

**R:** When a patient cries, you normally let them cry for a while until you see that they have colled down to some extent and then you try to resume the conversation. But when they start crying you cannot tell them to stop crying or do anything else, you first of all let them cry a little bit and then cool down.

**I:** It is important to help patients to manage the feelings resulting from their illnesses?

**R:** On their own?

**I:** To be able to manage or to control those feelings caused by the illnesses that they have, is that important?

R: Feelings for patients who have…I do not understand well.

**I:** For instance here, if a patient cries, it is probably caused by an illness that they have. They cry because of problems that they have for most cases. So, is it important to help patients who come to see you, to manage the feelings that are resulting from their illness?

**R:** It is very important.

I**:** In which way?

R: Because they may feel hopeless because of their illness and they think that they are going to die soon or that their life reaches an end. So, if you are able to talk to them and give them many examples that show them that there is no problem, you understand that it is important.

**I:** Is there something that you do to know if a patient understands what you are saying?

**R:** Yes

**I:** Why?

R: You can ask them a few thing on what you have discussed. You can ask them some questions about what you talked about and you see if they tried to understand.

**I:** Should a health care provider help patients to be involved in the health care that they receive?

**R:** A health care provider should do what?

**I:** Should a health care provider help patients to play a role in the health care that they are given?

R: Yes

**I:** How and why?

**R:**  Playing a role in the health care that is given to them?

**I:** Huh

R: It would be useful because for instance if you talked about how to protect oneself against illnesses. If you tell them how to do so and that they share it with their neighbors; if you discuss with them and ask them to share the knowledge with other people, you understand that it would be useful.

**I:** So, we have been talking for a while and we have discussed many things. Do you think you have something to add on what we have talked about?

**R:** Well, what I can add is that it is only now that I come to think about it because of these questions that you have asked me about. I was thinking that we can have a conversation with patients, but thanks to the many questions that you have asked me, I realized that we were not taking these things seriously enough. We work at the health facility, we did not give much value to these things but now I have realized that they are important. Now that we are sitting together, because of the questions that you have asked me, I understood that they have a great value.

**I:** Okay. So, do you think that there are other questions that you think we can ask health care providers concerning the conversations that they have with patients?

**R:** You can do what? Are there what?

**I:** Since the beginning, we have asked you many questions and you were answering them until now. Do you think there are other questions that we can ask health care providers in connection with the conversation that they have with patients?

R: Questions that you want to ask me now or you which you can ask…?

I: Which you can add on the questionnaire.

R: Aha!

**I:** Questions related to the conversations which health care providers have with patients in the consultation room.

R: If there are some of health care providers who regularly conversed with patients, you can ask them and see if that had a good outcome compared to those who did not regularly converse with patients. That is because a health care provider may have conversed with some patients and didn’t converse with other patients and then look at the outcome later for those he conversed with as compared to those who didn’t converse with. You can ask and see if anyone did that.

**I:** And then, what questions do you think we can ask health care providers concerning how to improve the conversation they have with patients?

R: You can ask them how long the conversation they have with patients takes, um, and maybe how long they talk. Did you ask me about that?

I: No, I didn’t ask you about it.

R: And also a standard period of time that a health care provider can talk with the patient.

I: How long is the specific period of time during which a health care provider should talk with the patient.

I: Okay! Thank you so much, I am really grateful for you!
