# Supplementary material for: Providers’ perceptions of communication with patients in primary healthcare in Rwanda
Source: PLoS One. 2018 Apr 4;13(4):e0195269. doi: 10.1371/journal.pone.0195269 (PMC5884556; doi:10.1371/journal.pone.0195269)
Supplement: S1 Dataset — (ZIP) [file pone.0195269.s001.zip › S1 Dataset/PPC-Provider 3.docx]

**PPC-Provider 3,**

I: Interviewer, R: Respondent

**I:** So, I would like to start by asking you this, “Can you tell us a little bit about the conversation that a patient has with the health care provider in the consultation room at the health center?”

**R:** Well, in reality the health care provider should have an in-depth conversation with the patient. However, it is a fact that today a patient enters and the health care provider asks them, “What are you suffering from?” and then the patient describes how they are feeling and then the health care provider examines quickly and roughly and then prescribes medications, um, sometimes he doesn’t even tell you how to use the medications – he just tells you “The person who will give you the medications is the one who will tell you how to use them” and sometimes the conversation does not happen as well as it should be owing to the fact that there are many patients. More often than not, a patient takes an initiative to ask the pharmacist, “How will I use the medications?” because the health care provider did not explain to the patient how to use the medications; but in reality, the health care provider should tell the patient that information, he should also tell them the root cause of their illness and advise them on the behavior they should adopt in order to protect themselves against the illness. Because nowadays we receive many cases especially for those illnesses that are called “chronic diseases”; if a patient has hypertension, the health care provider should tell the patient to go to a higher health facility, but unfortunately they don’t say so to the patient, they don’t even ask them questions to know the reason why the patient developed a hypertension. They should actually have a long and detailed conversation, which would make the health care provider understand the root causes that have led to the blood hypertension. So, you will find that those conversations do not usually happen; health care providers do not engage patients in such conversations as well as they should do because of many patients amounting to fifty, sixty or one hundred patients waiting for them.

**I:** What is the contribution of the conversation that a patient has with a health care provider to the work that you do in the consultation room at the health center?

**R:** When they have had a conversation, the patient gets to understand the causes of their illness and therefore they know how to behave accordingly. They understand and you work together – you give them medications and they also contribute to their recovery by taking measures on their own. It is good that the health care provider tells the patient how they should behave, but the patient also do take their own initiative and resolve, “I am going to give up such and such things”. The health care provider tells the patients what to do, but the patient also understands why they must do so, and therefore they participate in their own recovery.

**I:** So, tell us in full details how you think the best conversation between a health care provider and a patient is?

**R:** In reality, a health care provider should understand the patient. That would help him to know the patient comprehensively; because most often than not, the health care provider treats the patient of obvious illnesses and he/she forgets other factors that can cause the illness , like the family where the patient was born, things that have happened in their life; usually the health care provider doesn’t remember those things. And yet, there are many psychological illnesses that can become bodily illnesses, which we call somatization. So, we usually forget all those things and superficially investigate in the real root cause of the illness either because we have so many patients to see or because health care providers did not have training and therefore they do not go into much details. Additionally, there should be what is called “two-sided conversation”; the health care provider should not monopolize the conversation whereby the patient just keeps saying “Yes” to everything that the health care provider says, the patient should also commit themselves to do some things. The health care provider should not kind of force the patients to do some things, there should instead be a kind of discussion and sharing ideas; and then after discussing, they should make decisions together; a patient should take some measures according to their health condition. If possible, um, the trouble is that we usually have difficulty doing it but normally the health care provider should conduct a home visit to the patient’s home and see if they are putting into practice the advice they gave them. Treatment should not end in the consultation room.

**I:** So, you touched “training” and I had an immediate question for you. Is it necessary that health care providers be given training concerning how they should talk with patients?

**R:** That is very important, because that’s where things do not go well; they should talk to patients in a particular way. If I can give an example, there are patients who come to the health center after having taken drugs, and usually, health care providers feel that – there are even those who go far to say, “Why did people bring that patient here? They should have let him/her die at home!” but the health care provider should understand why – as the health care providers have not been trained to talk to patients, they think that patients are to be blamed of having caused their own illness whereas there might be other root causes that influenced them to have certain behaviors. Therefore, it is very important that health care providers learn how to better engage different types of patients in conversation in order to achieve the objective of treating them for their illnesses.

**I:** Do you think that the best conversation that you have with a patient can help you improve the way that you care for them?

**R:** As we said, the best conversation that one has with a patient helps them to participate in the care that they receive; such a conversation makes the patient understand that it is necessary that they take medications; it helps the patient to take steps of self-protection against the illness and commitment to abide by the advice that the health care provider gives them because a patient must also play some role in their treatment. If the patient does not play their own role, the treatment fails. Let me give you an example: people who usually have stomach pains – if you tell them, “Never eat such and such foods again. You should give up taking beer,” and explain to them that that beer damages their health, and that such and such foods worsen their health condition, they decide and commit themselves, “Oh, they always tell us to not eat this food, I shall never eat it anymore!” A good example is the hypertension that we have been talking about before. If you tell patients that they’d rather not eat fat or eat salty food, and explain to them that fat collects in their blood vessels, they realize in the end, “Well, I was used to eating such and such foods, but since now I won’t eat them anymore.” So, you find that there is a good relationship between the health care provider and the patient when they have had a good conversation. Additionally, the conversation allows mutual understanding and trust.

**I:** Do you think that enough knowledge in conversing with patients can enable you to better help patients that you receive?

**R:** Enough knowledge can enable a health care provider to better help patients they receive, but they also have to know how to use that knowledge. People can have enough knowledge, but they also need to have the required skills in engaging patients in the conversation. People may really have enough skills but in reality enough knowledge should be coupled with the knowledge of how to have an efficient conversation and also with enough time to converse with patients; because when one has too much work, when there are so many patients waiting, they do not have a good conversation with the patients even if they may have a required knowledge. I therefore feel that the best thing is that an effort should be made so that a health care provider has the knowledge, receives the training and does some practice regarding engaging patients in conversion and also have enough time – because it is reported that one health care provider who has the title of medical doctor has to see thirty thousand patients, that person can by no means have enough time to converse with patients. For instance here we receive one hundred and fifty patients and one nurse cares for fifty or sixty or more than fifty patients; so a nurse will have difficulty conversing with patients in a better way. There are still challenges because of many patients, and patients would like to be served as quickly as possible too.

**I:** Yeah, let us continue. Did you receive any training that help you to improve the way that you talk with patients whom you receive?

**R:** Yes, I got the training. When we were at school, we had a course about engaging patients in conversation.

**I:** So, what were those courses?

**R:** Um, the course was both in French and in English, it was entitled “communication skills”. It talked about how you engage a patient in conversation, how you build rapport with a patient, how you tell them to sit down – first of all you greet the patient after having given them a good seat, then you make them feel comfortable. After that, you start to tell them your goal and then you engage them in conversation. You talk to each other and you ask them questions that they can answer and then you reach a conclusion and then you make decisions; you help them to make a decision concerning their problem.

**I:** Huh, is that useful?

**R:** Yes, it is very useful because when the patient does not get involved – for instance if we teach people to protect themselves against malaria but a patient doesn’t take and use a mosquito net, or they don’t close the door before the mosquitos enter into the house, they don’t remove any grasses around the house, we can give them coartem (pills for malaria) but they relapse soon. We have been noticing that there are patients who come to the health center twice in a month to seek out heath care for malaria. So, it is always useful if the patients take their own decision and also understand the problems that they have and why they have them and also have enough knowledge about how they can protect themselves against them.

**I:** Huh, coming back to the point of training and improving the way you, as the health care provider engage patients in conversation, do you think that it is useful to have those trainings?

**R:** Yes, it is useful. Normally “learning is a lifetime journey”; so it is necessary that people continuously learn new things and make self-evaluation and see if they can put into practice what they learnt. So, it is important to review what they learnt before and if there are any new changes, then they try to understand them and put them in practice.

**I:** How would you rate your ability to engage patients in conversation on a sale of 1 to 10 points?

**R:** That’s what we were saying – owing to too much work because of shortage of staff in health centers and because of many patients that one has to care for, one would give oneself six and half points out of ten. For instance if someone has had an accident or if someone knocks themselves down when they are drunken, we immediately do all the necessary primary care for them but we don’t have time to talk with them. Many times we receive patients and we immediately start treating them without prior conversation with them.

**I:** Well, what you said are other factors like external factors. Now still on this question of grading yourself on a scale of one to ten points, how much points would you give yourself for your know-how to engage patients in conversation in a required way?

**R:** As I told you, there are a few courses that one had at school, but in reality they are not enough; that is the reason why I said that training is needed so that people are given refresher courses; for that reason I would give myself six points at least.

**I:** Yeah, you understand that you lack four points so that you score ten points out of ten; would you like to tell us where you have a gap in your knowledge that it prevents you from effectively engage patients in conversation with you?

**R:** The gap is usually there especially because patients have different illnesses. There is a specific way of talking to a patient who lives with HIV/AIDS, there is a specific way of talking to a patient who has tuberculosis, there is a specific way of talking to a patient who has trauma. All those are examples of different approaches that can be used to talk to different types of patients. The health care provider is not equipped with enough knowledge as per how to engage conversation with any type of patient who comes to seek health care because they have also various problems. There are patients who are widows, there are women who do no longer live with their husbands; there are many types of patients for whom one should learn how to talk to in accordance with their specific problems. For example if patients have sexually transmitted diseases, there is a health care provider who would tell shout at them, “Why did you get infected? What happened?” So, there are many approaches that one should learn with regard to how to talk to patients, and unfortunately, one does not learn all those approaches at school. Additionally, it is a long time since one finished one’s studies and therefore one needs to be given a refresher training.

**I:** So, what would you like to improve on?

**R:** Well, I may give an example right now. The way I can help someone who lives with HIV/AIDS, someone who had a misfortune of contracting HIV/AIDS, um, someone who has experienced domestic violence; we need to know how to help people who have had those problems. There are many different areas in which people need to be trained so that they are able effectively to interact with patients and treat them in a way that they all agree with.

**I:** How does your collaboration with other co-workers influence positively the conversations you have with patients?

**R:** Well, that’s a good question. That is an issue that we discuss in every morning staff meeting. It sometimes happens that a patient comes at the health center multiple times but this happens because both the health care provider and the patient did not have a detailed conversation and therefore the health care provider did not really understand the root cause of the patient’s illness, which makes it that the patients comes frequently to the health center, because the health care provider failed to figure out the real cause of the illness. So, we discuss that, we share it in the morning meeting, and I tell other health care providers that the reason why a patient comes multiple times is that they did not ask them properly so that they would get to uncover the real illness. As a result, when the health care provider has not had a detailed conversation with the patient, they keep returning to see health care at the health center because their real problem was not well understood and that the health care provider did not know the true illness of the patient. So, we share this in the morning meeting and I tell the health care providers how they should ask questions to patients, which is referred to as “questioning”, by doing so, they can get to know more information on the patient’s family. There are people who have a given illness, but no-one knows that the illness develops from the family because they did not question thoroughly the patient so they can know the background and root cause of the patient’s illness.

**I:** So, coming back to yourself, your collaboration with other health care providers, does it play a role in making better the conversations that you have with patients?

**R:** Well, things go well. When we discuss it as I said and that I tell them what they should do – there have been some improvements. Improvements are there because if you look at patients who are hospitalized, there are remarkable improvements because health care providers were able to follow up the patients, and if they encounter with any problem, they call me and say “We have used the approach that you told us and we had good results”. What I can add on this is that even if we try and teach one another, one does not understand or know well unless one receives a training, one cannot fully understand something as someone who sits before a trainer, or as someone who has gone somewhere specifically for a two, three, four or even five day training. It is good to share knowledge among the staff because they retain something, but it would be much better if health care providers would receive training on engaging patients in conversation. Because you find that most of the ministry of health’s trainings focus on some domains, but it is very rare that they plan training for health care providers on how to converse with patients. We have never heard of any training which talked about how to hold a conversation between a patient and a health care provider.

**I:** Huh, thank you. Do you think that it is useful to ask patients if they have come to seek out health care expecting something special?

**R:** That is important because a patient sets off because they are suffering. Although the patient believes that the health care provider is the answer to their problems, but they do not know that they are the ones who must provide the answer for their own problems, they are the first ones to give the answer. The problem that is there is that a patient believes that any health care provider, even a nurse, is the one who has the answer for their health issues while it is, in fact, the patient who largely has the answer.

**I:** Huh, is there anything that you do to know if a patient would like to know more information about their own health?

**R:** Well, that happens sometimes. It happens because some patients ask us, “why did I have this illness when I did not behave in a risky way?” for instance if someone is found with high blood pressure, they ask you, “How comes that I have hypertension when I don’t drink beer?” and when you ask them many questions you find that they really tried to protect themselves. So, you see that they need to know the reason why such and such health issue has happened to them when they think that they have been trying to protect themselves. For instance if one is diagnosed with malaria, they ask you, “I sleep in a mosquito net, there are no stagnant water around my house, I close doors and windows, how comes that I have malaria?” You find that they are curious to know why certain health issues have affected them.

**I:** That is the side of the patient, but you as the health care provider, do you do anything in order that you know if the patient needs such information that you were explaining?

**R:** Yes, we ask them so that we are sure that we understand each other and make sure that what we are doing is not useless. You ask them and know what knowledge they have and in order to give them more knowledge so that you share the knowledge and therefore they know what they have to do to protect themselves; this is good because there are some patients whom you find that they don’t have the knowledge; for example they can tell you, “I got malaria because I ate a sugar cane” and you realize that they really don’t have knowledge about different illnesses. For example for the issue of hypertension, there are people with hypertension who will tell you, “I eat too much iodized salt; I sprinkle it over the food”; so you teach them that they should eat little salt and inform them that too much salt takes water away; by doing so, together you find ways of curbing and reducing their illness.

**I:** So, do you think that it is necessary to explain to the patient all things that they want to know?

**R:** Yes, it should be necessary except that, as I told you, we have the problem of shortage of time, but in reality a patient should know everything that they would like to know about their illness so that they are fully involved in finding a solution to their own problem.

**I:** So, you once touched “decision making” between the health care provider and the patient. Is it important to make patients be involved in making decisions regarding the health care that they would like to be given?

**R:** Yeah, that thing is very important because if patients are not involved in decision making, the health care provider would only give medication today, tomorrow and repeatedly for the same illness. Because when they are involved, they protect their health by abiding by the advice that you give them. For example we treat children who are malnourished and we give them milk and they get well. But if they got recovered and resumed having a bad nutrition, they would come back once again to ask for milk. So, it is a good idea that we teach parents how we can fight against malnutrition; their child receives milk because they have malnutrition but the parents also must receive an education about how they can find other foods that work in the same way as the milk works or we teach them how they can find the milk on their own. By so doing they get involved in making sure that the same problem does not happen to their child anymore.

**I:** If a patient has a preference about health care, should the health care provider attach importance to it?

**R:** Yes, we must respect the patient’s preferences as long as they make a choice that lead to the solution of the problem that they have.

**I:** In case the health care provider shows their emotions, how do the emotions impact the conversation that they have with the patient?

**R:** Emotions play a role, they play a positive role in understanding a patient. Sometimes you may have emotions that are not showing that you are sympathetic for the patient. For instance if someone has got sexually transmitted diseases and when you see them you exclaim, “Hey, what’s this?” this is an emotion which does not help to address the problem. But if someone shows you their illness or if you see their illness and ensure them that there is a cure for it and then you tell them that there is possibility that they can recover from the illness and don’t blame them for having been affected by it and show them that you are caring and supportive and that you are going to do your best and try to find a solution to their problem, the patient will be more hopeful and they will trust you - there are some patients who call us later on and say, “My illness has recovered” and some others also come and say, “I am very grateful for you! You helped me a lot, I was going to die of the illness but you saved me.” However, if you show a strange emotion, the patient gets scared and say “Oh my God, I die” additionally, if you blame them, they see that you do not care for them and this is not good. I can look at emotions in two ways: an emotion that shows that you feel the patient’s pains and the emotions that make you blame the patient, which is bad and unfavorable for the patient.

**I:** Is it acceptable that health care providers exhibit their happiness or sadness when they are with patients?

**R:** Yeah, health care providers are also human beings so they can feel happy when they are together with patients, especially when a patient comes to report that the medications that you previous gave them have responded to their illness. This is usually when they come for post treatment checkup; this pleases the health care provider and they have what we call “self-confidence” and they pride themselves because the work that they did has given fruits. That cannot prevent the health care provider from being happy. A health care provider can also have sadness, for example if he receives a child who has been raped; the health care provider deplores that because he/she is also a human being but nevertheless, they should not be overwhelmed by the sadness; instead they should exhibit emotions that lead to finding ways of preventing the raped child from suffering any side effects or consequences. So, the sadness cannot prevent the health care provider from caring for the patient. This happens to us sometimes and the emotions compel us to help the raped child even more than we would care for someone who has headache or malaria.

**I:** Huh, do you think that patients can be worried about sharing their health issues with the health care providers?

**R:** Yes, there are some patients who are worried and there are even those who refuse to admit that they have a problem. For example people who have contracted HIV/AIDS, some of them are very reluctant to report that they are HIV positive. We used to send social workers to conduct home visits for those people but they warned the social worker, “Please don’t come to my home, I don’t want you to come to my home.” There are those who are worried about telling their problems to health care providers, and there are others whose problems we get to know only after probing, they beat about the bush but we end up knowing their real problem. I can give you an example; if you talk with a young girl who has got pregnant, you have difficulty knowing that she is pregnant. She hides it from her parents, she hides it from the whole society and she wants also to hide it from health care providers. Most often young girls who are members of these churches which are known to seriously check if brides-to-be are not pregnant before they get married – we encounter with such cases most of the time – they are afraid of letting the health care provider know when they are pregnant because they fear that they can disclose information and consequently spoil their marriage. If it is a young girl who is soon to get married, the church leadership can ban her marriage from happening. We usually encounter with that problem.

**I:** So, what can be done to solve that?

**R:** Well, what can be done is to know the best ways of engaging a patient in conversation so that they feel free with you and then they trust in you; also the health care provider should be, I would say, you should be the patient’s parent, you should be the patient’s secret holder; in that case the patient goes home with confidence that you will keep their secret. It happens sometimes that someone spends the day at the health center and then they come and tell you, “I have been waiting for you, I cannot let so-and-so know about my problem.” You find that patients have preferences when it comes to telling their problems to health care providers, they say “I will talk to so-and-so, but I cannot talk to that one.” We encounter with those cases.

**I:** What is your experience when you are conversing with patients whom you think have low level of education? For instance those ones who did not learn how to read and write?

**R:** Yeah, there are problems because when someone did not learn how to read and write, they have low level of knowledge, and it requires that you explain to them a lot of things. When it comes to the one who studied, you explain to them things and you find that they seem to already know some things while for the one who didn’t study, you have to tell them so many things. On top of that, when you are making decisions, you are required to repeat the decisions multiple times so that they can understand. You repeat and repeat for them even the way that they have to use the medications. For example if an illiterate patient has a sexually transmitted disease – we usually give them ciprofloxacin or doxycycline and if need be, we also give medications for preventing trichomoniasis and candidiasis – you see that it’s a combination of four types of medications; you therefore have to explain to them how to use these medications. You give them a piece of paper which has an image of the sun and the moon so they know when to take the medications. But for the other one who studied, you just tell them the time when they have to take the medications and they immediately understand, you only have to write the time of the medications while for the illiterate one, you show them the sun and tell them that it means they will take the medications in the morning and the moon to mean that they will take the medications in the evening; we usually encounter with such problems. Some patients also come and tell you, “I used the medications but they didn’t help me”, that is an example.

**I:** How do you use your communication skills to engage that category of patients in conversation?

**R:** Well, for patients who are in this category it requires you to abase yourself and seem equal to them. That’s why I was telling you that for those others who studied, you explain to them and they understand and when ask them they answer you correctly. But for the others who didn’t study, we have to show them pictures and explain, “Look, this is a moon which is setting on, and this is the sun which is setting on.” So, it requires you to abase yourself and show and explain to them the meaning of the pictures, then they really understand that it is for instance the sun which shines over a mountain. In order to understand each other, you have to abase yourself and explain to them in simple language, you don’t have to use complicated language. You have to use the language that they understand, you don’t have to tell them in English or in French, you instead use their language, and you even try to illustrate the time when they will be taking the medications, so you can tell them for example, “Take the medications when students come back home from school” or you can tell them for example, “Take the medications when goats are brought back home from grass yard”; you basically have to illustrate the time using things that are closer to them, what they see at their home.

**I:** How does the Rwandan culture impacts the conversations that a patient has with a health care provider?

**R:** Yes, the Rwandan culture has an impact especially nowadays; today people can wear shoes – it used to be said that one should go to see a health care provider when one is not wearing shoes. This is something good, now people can wash their bodies, they keep themselves clean, nowadays no-one can dare to go to see a health care provider when one’s clothes are dirty, today people know that they have to wash their clothes compared to how it was in the former time. A health care provider is no longer considered like a superior person whom people can fear to talk to, people have known that the health care provider is there for them, they know that they have to see him/her, there are many factors that nurture a relationship between health care providers and patients: we visit patients at their homes, then we share foods and drinks, sometimes there happen public events to which patients and health care providers attend; so today a health care provider is not considered like a scary person as it used to be in the society.

**I:** What impact does that have on the conversations that you have with patients?

**R:** Well, patients feel that the health care provider is a fount of knowledge on their problems, so they go and see the health care provider, have a discussion and learn more from them.

**I:** From your perspective, what are the factors that spoil the conversation on the side of the patients?

**R:** Well, on the side of the patient, there are still a few patients who still think that a health care provider is a scary person, there are those who still feel that a health care provider is a superior person. Another factor is the illiteracy that we were talking about; so if the health care provider does not know how to abase themselves, then they cannot understand each other during the conversation. Another factor which has been noticed is that patients can have a different belief. If it is a patient who has been told – I give you an example – I recently heard on the radio Rwanda that patients who live in the catchment area of the Kiziguro hospital go to seek traditional healers before delivery if they think that their pregnancies have been bewitched; then traditional healers make cuts, give them grass medicines; those factors can be a challenge and impede the conversation between the health care provider and the patient if patients still hold on the former practices. For example in this community we are fighting against people who claim to be able to *treat ikirimi (swollen uvula and thus makes it painful to swallow) and those who say that they can guhara gapfura (To treat angina by scraping the throat with traditional medicines)* – all those are the challenges – people say that this illness is confirmed when they introduce burdock in the patient’s nostrils and they bleed from the noses. So, all those are the beliefs that people still maintain and they are some of the challenges that health care providers encounter with while they are trying to discuss with patients because they believe in false treatments.

**I:** Do you think that there are other factors that can hamper the conversation between the patient and the health care provider?

**R:** Yes, there are other factors; for example if it is a patient who has sexually transmitted disease and that you ask them to bring their partner, their spouse, or any other person with whom they have a sexual relationship, they refuse to bring that person or just tell you that they do not have any partner or spouse or they tell you that their partner has gone away; so it’s a problem in that case. When you request her, “Please do tell your husband to come here?” and she answers you – yesterday I had a similar case; I received a woman and when I asked her to bring her husband, she told me that he lives in Kibungo and yet, when I called him on telephone, he told me that he has gone to Muhanga city but he had been at home. So, sometimes there are challenges and you cannot succeed in curing a woman if you don’t treat her partner too. We usually have such cases. Also for girls who are pregnant, when you tell her to come with her partner so that they are tested for HIV/AIDS in order to save the fetus, she tells you that the boy who impregnated her lives too far away whereas he lives in her neighborhood. So, you really understand that things are not going as well as the health care provider would like them to and therefore the solution to the patient’s problem is not as satisfactory as the health care provider want it to be.

**I:** From your perspective, what are the factors that spoil the conversation on the side of the health care provider?

**R:** On the side of the health care provider as we said, there are those who work in a condition that I would call “under stress” I don’t know how to explain it in Kinyarwanda, but owing to many patients that a health care provider must see, they ask themselves, “When will I be done? They are so many!” The fact that a health care provider has many patients to see, he/she doesn’t have enough time to converse with them. Another factor is insufficient knowledge. That is the major factor because in any case even if you have to see many patients, you can at least tell them something helpful if you have enough knowledge of how to converse with patients although you may not maximize; you can try and tell them something. So, there is the problem of insufficient knowledge. The major factors are just many patients, insufficient knowledge and the lack of a regular organization at work. We are familiar with the fact that people come to supervise how tuberculosis is treated, they come to see how antiretroviral medications are administered but we never saw anyone who came to see how the conversation between health care providers and patients is held. Well, when no supervision is carried out for a specific task, people tend to feel that the task does not have to be done. So, when something does not fall under the performance contract of the health facility, that thing is ignored. We lack practice, we need to have it integrated into our system so that we care for our patients.

**I:** Are there factors associated with the functioning of the health facility that can hamper the conversations?

**R:** Yes, as we have been saying, when the health care providers do not receive training, each one develops their own way of talking to patients and therefore there is no harmony in conversations, and as a result the way one converses with patients is not the way so-and-so converses with patients. There should be a universal way which is known so that each health care provider says, “I have to engage a patient in conversation like this”; therefore they would be able to begin from the beginning instead of beginning from the end and they should also be able to know when to start the conversation and for how long.

**I:** Can you give us examples of things that are difficult to explain to patients who come to see you?

**R:** Things that are difficult with regard to conversation with patients, sometimes you find that there are various age groups of patients; in that case it is very difficult; for instance if we want to teach them about using family planning when there are children, too young children, it is difficult because children are not really concerned with the conversation. On top of that, patients have different illnesses and each one wants their problems to be solved. So, if we want to teach them about stomach worms while one has not come to seek health care for that particular problem, you have difficulty teaching them because you basically have to choose a problem on which the education will be centered so that they feel that the education they are being given will help them to solve their problem. So, sometimes is it difficult to educate patients.

**I:** Huh, let us come back to the time of consultation, do you have an example of things that are difficult to discuss with patients?

**R:** In reality it shouldn’t be difficult to have a conversation with patients. The only problem is insufficiency of knowledge on the side of health care providers. For instance we receive patients who have epilepsy and there should be a specific approach to be used to talk to that type of patient. It is caused by the fact that health care provides do not receive training with regard to conversing with such patients. Otherwise I think that what is important is to know how to engage various types of patients; and as for any patient who is able to hear, speak and understand, there shouldn’t be any problem regarding the conversation with them.

**I:** When it happens like that, what do you do?

**R:** When it is difficult to hold a conversation with a patient because of lack of knowledge, one manages to do it in such a way that one feels – because we have conscience, so we do something that we feel “This can hopefully help the patient” but in fact, you don’t know whether or not the way you converse with that patient is the best way. You do it to the best of your knowledge but it may be true that you lack some other knowledge. Yes, that happens sometimes; you say to yourself, “Although I have conversed with them, but I don’t think my goal is reached. I don’t know if I have guided this person in a good way.” If for example it is a person who has HIV/AIDS because patients bring their test results into consultation room, you start a conversation with them but you don’t know exactly if the conversation is the one that is appropriate or if you missed something and so forth; so you see that there is an additional knowledge that one needs to have so that one is able to have a conversation with patients.

**I:** In your daily work, did you ever receive a patient with a mental illness and you had difficulty engaging them in conversation?

**R:** Yes, that happens so often, especially during this period of commemoration of the genocide, we receive people who have trauma and you don’t know how to approach them, you don’t know how to start a conversation with them; maybe what you tell the patient is dependent upon the problem that they have. So you ask yourself, “What is the first thing that I have to do for the patient?” and then you make a conclusion and say, “As I am a grown-up person who studied, let me talk with the patient” but in reality you don’t know where to start; that problem happens sometimes. Maybe slowly by slowly you approach the patient, you give them water and see if they take it and if you are lucky, the patient’s condition subsides but in fact you don’t use any methodology which is appropriate to help you achieve your targeted goal.

**I:** Did you have difficulty conversing with patients depending on the nature of their problem, like deafness, numbness, blindness and so forth?

**R:** Such patients come to seek health care but many of them come with their caregivers, you have a conversation with the caregiver. For the blind people, they are able to hear most of the time, but when someone is a deaf, it’s a great problem so you speak with the caregiver because usually when someone is a deaf, they come with a caregiver.

**I:** So, did you receive a patient with whom it was difficulty to have a conversation due to their personality or their perception?

**R:** It happens very often, here come people who have had mental problems, they answer you things which are different from what you ask them, you realize that they are in another world. In that case, it is difficult to have a conversation because they are not capable of sharing their ideas with you whereas you basically have a conversation in order to make changes in their life. So, we usually receive them and we talk but we see that the conversation cannot lead us to any change, so we talk to the patient’s family member except that in such a case we refer the patient to other health facilities so that they register them in their program and then talk to them after getting better.

**I:** So, at anytime during your work, did you ever receive a patient with whom it was difficult to have a conversation because of another health issue that is not among the ones we have discussed?

**R:** Yeah, we receive those ones, there are patients who come and when you talk to them, either they just ignore you or they simply burst into tears because of their sorrow or because of their feelings. Their feelings subside little by little and then you start the conversation. We sometimes receive people who had tried to commit suicide and when you ask them why they wanted to do that, what happened to them or how they are feeling, they just ignore your question and start crying. This is usually because most of them come after they have already started to regret what they had wanted to do and therefore they fail to speak; but slowly by slowly you approach them and then you talk, and you explain to them that they wanted to harm themselves and reassure them that their life will continue even if there are problems.

**I:** Is it necessary to tell patients about an illness or any other problem that you think they have?

**R:** The illness which I think they have?

**I:** Yes, is it necessary to tell them about it?

**R:** It is necessary because a patient has right to know their problems so that they know how to behave accordingly. Because if they don’t know about it when there is something that is causing the illness, they cannot avoid it. They have to know their illness and if you give them a medication, they also have to know how to take it. For example people who live with HIV/AIDS, if you do not explain to them that the HIV virus destroys the body’s immune system and tell them that they need to take their medications each single day in order to curb the virus, they would take the medications but stop taking them as soon as they feel better. But it is necessary that you explain to them the nature of their problem and how to solve it so that they help you to reach your goal.

**I:** What would you tell a patient if you failed to know exactly their problem?

**R:** If the patients problem is not known, because it also happens sometimes especially because, of course in health centers we don’t have equipment, we don’t have x-ray machine, we do not have a machine that can help us to do abdominal tests, so we give them medications and tell them to take them as well as we instruct them and ask them to come and report how they are doing after taking the medications. If they do not get well after finishing the medications, we refer them to other higher health facilities where they can do more tests. We really admit our inability to examine all tests and that we cannot do all things; but we explain to them thoroughly because there are those who go saying, “Oops, I have gone to the health facility but they failed to cure my illness” and they feel that if the health care provider at the health center was not able to cure them, then it is time to resort to traditional medicine whereas there are other higher health facilities which would help them. We do not tell them that health care ends by there, we instead tell them that there are other health facilities that have equipment that can do tests that we aren’t able to do.

**I:** Concerning medications, is it important for the health care provider to explain to the patient the type of medication that they give them, the way it works, how to take it and any side effects that it may have? You once touched that earlier.

**R:** Yeah, that is something very important, because if you don’t explain to them how to take the medication, they might even take too much quantity believing that they can get better very quickly and therefore they might have severe adverse effects. That is why you have to explain to them the functioning of the medication. If it is a medication that kills viruses that are in the body, you emphasize that they should never cease to take the medication or take it later than instructed because it may not kill the viruses; and you also emphasize that they should take the quantity that is indicated because if they take too much of it, then they will experience side effects. All this information is given to the patient so that we can achieve our goal, otherwise we would create new problems for the patient.

**I:** Some patients in Rwanda report that they are not given enough information concerning their medications. Is that true as per your experience?

**R:** Actually that is true, there is truth in what they say, but there are health care providers who try and explain to patients as we said before. If you go to Kagbayi hospital at the OPD (Outpatient department) and see how many people there are, it is not easy for a medical doctor to take time and educate each and every patient who is there; that is the reason why patients complain about delaying, the service delays because patients think that the doctor just prescribes the medication, gives it to the patient and they go outside, but they don’t know that explaining the patient’s problems takes time and that it plays a great role in their recovery. So, you find that people complain about that but it is very useful to explain to them. Additionally, as I told you, health care providers do not have enough time to explain that information to patients because they work under stress and therefore they do not tell to patients how to take their medications as enough as needed. Also, there are medical doctors or other health care providers who prescribe medications and give to patients and say, “Go and take your medications at the pharmacy’s window, they will explain to you how to take them” whereas he should explain to them how to take the medications on the spot. Even if the pharmacist can explain to the patient how to take the medication, it should be a repetition of what the doctor has told the patient in order to help them remember even something which they might have forgotten. As patients say, they leave their home and go where there are strong buildings, so there is change in their mind because of the new environment and therefore when they leave the doctor’s room or the nurse’s room, they may not understand well the instructions and it would be very useful if the doctor would explained well the instructions; it would be better if the pharmacy officer repeats what the doctor has already said.

**I:** Do your work conditions or does your situation at health center impede the effective conversation between the patients and you?

**R:** Well, that problem shouldn’t happen actually because the place where you meet with a patient is private and no one else is there, it is a place that you can close; in short that’s what we call confidentiality and it is secured because people do not treat patients in an open place or in a corridor; it is rather a closed room. The only thing that can hamper those conversations is lack of know-how and the stress, but it is mostly lack of knowledge. If people were trained and make a commitment to doing it, there wouldn’t be any reason of not having the conversation, I would say that it is because we are not fully committed to doing it; we developed the culture of just prescribing the medications and give to patients and they leave.

**I:** What do you do if a patient requests to be referred to the hospital when you think it is not necessary?

**R:** Well, that happens very often, it happens most of the time; patients request that. However, if we see that it is not necessary, we explain them why. Myself I explain to the patient that they are going to waste their money and make travels which are not necessary while they would use their money and time for other things. I explain to them that they should wait and take medications properly and if they have another illness, I tell them that they should respect appointments; sometimes it is necessary that we teach patients. There are patients who get treated, discharged and are given medications but they say, “I want to return to the health center” or they simply tell you, “I want to go to the hospital” but when we think that it is not necessary, we first of all teach them. When we find that the patient has a problem that must be dealt with by a medical doctor, we regard their request as her rights. There are others who come and rudely ask you, “Me, I want a transfer!” A transfer to go and have a consultation for their sight whereas we also do that test here. But they neglect our services and feel that they must go to the hospital. In that case we do not deny them a transfer, but we mention, on the transfer, that they want to go there on purpose. We do respect the patient’s right entirely because when we do a test and see that the patient needs to go to the hospital, we immediately give them a transfer. But when a patient says, “I don’t like your service” we let them go and we do mention that they go on their own will.

**I:** Do you have any difficulties conversing with patients about health subject in Kinyarwanda?

**R:** Some problems happen. Some problems happen especially with regard to conversations about reproductive health; as I was telling you earlier, you can have difficulty talking about sexual organs because of age groups of patients who are there because you know well that in Rwandan culture we were taught that talking about sexual organs is a sin and that sexual organs are so respectful that they should not be discussed in the public; therefore we try to evade that when we are addressing patients who are of different age groups. When you are in the consultation room, you try to talk about it openly because it is a one-in-one conversation, even if you may not call the spade a spade and say the name of the sexual organ the patient understands what you mean. You don’t need to teach them the whole anatomy and say “This organ is called this” and so forth but if the patient has a wound on their sexual organ, you just tell them that they have a wound and we feel that it is enough.

**I:** You said so and you reminded me of a question that we talked about earlier concerning how the Rwandan culture impacts the conversation with patients. Is that connected to this?

**R:** That is very true because in Rwandan culture I would say that sexual organs are sacred or they are a taboo I such a way that they are not discussed; we found it like that when we were born and we believed in that and we take it like that today, that is why people try and evade talking about it; but when you talk to an adult person, they don’t have difficulty understanding what you mean, though it is sometimes difficult to explain. Additionally, I personally don’t think it is useful to say “Sexual organ X has a problem”, I just show to the patient that there is a wound somewhere on their sexual organ. Until now I have not yet understood the importance of telling the names of the sexual organs to patients; I don’t think it is important at all.

**I:** So, how does that influence the conversation that you have with the patient?

**R:** Since the patient already has a problem, they feel pleased when you tell them that they have a wound at a place that they couldn’t even see; they feel happy and they say to themselves “As my problem has been identified and that I have got medications….” what tells you that the patient is happy and that they understand you well is that when you give them another appointment, they do respect it and you see them come to see you. If it is a married woman and that you ask her to come the next day with her husband, she does come with him. That shows us that the conversation that we have had was useful and that it will bear fruits in the future.

**I:** But still on this point, you see, because of the Rwandan culture there are some words or terms that you cannot use when you are talking about or describing sexual organs; does that influence in one way or another the conversation that you have with the patient?

**R:** Yes, because evading to say it by its name, respecting things and abiding by the culture is a good way that makes the conversation go smoothly; because there are some things that you can say and the patient loses trust of you and say, “The health care provider is a mentally ill person; he says things as they are!” So, I think that one has to be careful so that patients do not say that they have met with a health care provider who has lost their mind.

**I:** So, to close the subject that we were discussing, if I got you correctly you said that a patient can trust you because you have not used some terms or words which are not culturally accepted in Kinyarwanda during your conversation and therefore the patient feels free, but they become uncomfortable when you use these words?

**R:** Huh, yes because that is the respect of the culture. If one doesn’t respect the culture, people do not trust them. We work with people who did not study up to a higher level and who did not go abroad to study, but there are also other staff who came from abroad but in our community, we are required to behave like community members; if you do not behave like them, it would be very difficult to get on with them. It requires that you try to be like them, you speak like them and you show them that you are equal to them, it is only then that they can feel free to talk with you.

**I:** Is the fact that you were taught in French or in English a problem for you when it comes to conversing with patients in Kinyarwanda?

**R:** That is not a problem at all, the problem is insufficiency of knowledge. The language of instruction should not be a problem for us, it should rather be an additional value that helps us to know more and study more; translating what you know into Kinyarwanda is not a problem.

**I:** So, what do you do when you have to explain English or French words that do not have equivalent in Kinyarwanda?

**R:** Well, it is not difficult actually, you do not go into details to say, ‘This is antibiotics”, nope. You just tell the patient ‘This medication kills viruses”. If we are giving them coartem for malaria – there are people who already know that coartem is not Kinyarwanda – you tell them that it is a medication that kills malaria virus. We also add that if they share the pills with someone else the viruses will not die completely and that the viruses will survive and multiply and make them sick once again. There are some medications that are already known to people but there are also others which we try to find how to explain. You cannot give antiretroviral medication to someone who has HIV/AIDs and tell them that you give them limivivine, that is not necessary; you simply tell them “this medication is strong, it reduces the strength of the viruses; you have to take them at a regular time” I think this is enough, we don’t need to go into lengthy details; you would say it if the patient is a health care provider like you. It’s no use telling a teacher that you have given them limivivine, you cannot tell it to a cultivator or someone else, it’s not very important. However, if the patients would like to read, you should let them read the name of the medication or you could also tell them “this medication is called ABC” so that they can differentiate it from others but it is useless to say it to an illiterate person. So, this does not prevent us from conversing with patients and make them understand what they have to do.

**I:** When you are conversing with patients, do you encounter with other challenges that we didn’t discuss?

**R:** In reality I think we have discussed the challenges in general, but there are many things that prevent us from having a good conversation with patients. For example you see that patients use medical insurance, so if a patient comes to the health center the first thing that you ask them is “Which medical insurance do you use?” If they do not have a valid medical insurance, the conversation is terminated and you do not meet once again anywhere else. That is a very big challenge.

**I:** If I understood well, do you want to mean that if a patient does not have mutuelle, it means this will cause a problem when you start the conversation?

**R:** If a patient doesn’t have a medical insurance, it means they will not be given health care too. Except that we use our critical thinking and tolerate when the patient is very seriously sick, but when the patient comes to seek health care on their own, walking, we ask them to look for money to pay so that we are able to serve them as usual. But if the person does not have money, they don’t even go into the consultation room. Then, once they reach in the consultation room, they become yours and you receive them. So it usually becomes a problem when a patient does not have mutuelle. Some of such patients come and just say “I go back home, I don’t have mutuelle” and they leave after having arrived here because there is no other option. Normally having a mutuelle is a must, so when they don’t have the mutuelle, it means they had not respected the must and their problem cannot be solved.

**I:** So, do you ask them if they have medical insurance when you are in the consultation room?

**R:** It’s not a problem in the consultation room because they come there after having passed by the insurance service because a patient goes to medical insurances first of all.

**I:** How can that have an impact on the conversations that you have if that has to be checked early before coming into the consultation room?

**R:** There is no problem in the consultation service, what I was saying is that a patient comes to the health center to seek help which includes medication and conversation. There are even those who come and we have a conversation but they go back home without that you prescribe any mediations for them. But if there happens a problem at the medical insurances service, there is no way that the patient can see you, that’s what I was saying. For a patient who has come into the consultation room, you cannot have a conversation only if they are deaf but I said that even if one is in a coma, you tell their relatives the reason why they are in a coma. We recently received a person who had drunk too much drugs, he had drank those little beers that are locally knows as *suruduwili (small Uganda waragi beer)* and no sooner did he arrive at the health center than he died. He came agonizing and he died soon. In that case, you try to counsel the relatives who have brought the patient. In fact when a patient arrives before you, nothing should prevent you from conversing with them or with the people who have brought them to the health facility.

**I:** What can be done in order to improve your knowledge of engaging patients in conversation?

**R:** It’s like what we said before; there should be organized a training. People should not stay there thinking that they know how to do things while they may be doing some things in a wrong way. They should be able to know when they are doing things in the right way. Health care providers should be able to know how to approach a specific type of patient, things should be standardized, there should be something like specificity if I can say so, a particular way of talking to a specific type of patient. Health care providers should be equipped with knowledge that enables them to receive and talk kwith any type of patients. For instance there are prostitutes who are well known, you cannot talk to those people like you would talk to someone who has malaria. You must have some specific skills that you use to converse with such a patient and then persuade them to no longer have unprotected sexual intercourses and you can even help her understand that she should stop doing prostitution. So, all those things require you to be knowledgeable; health care providers should have that course at their formal education and later on they should receive trainings.

**I:** Patients are different and this means that they also communicate in different ways. How do you adapt your knowledge to the different ways in which patients express themselves?

**R:** That is why I said that there is a need of communication skills, you try to adapt yourself to the patient’s communication style. You try to be on the side on the patient. If it is a patient who has sexually transmitted diseases, you side with themselves; if it is a patient who has malaria, you side with themselves too; and then for each type of patient, you should be able to know how to talk to them, that is why I said that it requires that health care providers are skilled, it also requires them to have enough knowledge on everything.

**I:** What can be done so that a health care provider helps patients to have a better conversation during consultation?

**R:** In order to have a better conversation with patients, the health care provider must first of all give them a good reception, there should be a room where they receive those patients; in short there should be a quiet room, a private room where patients will feel comfortable and talk about their illness. The health care provider should receive patients well, abase themselves, listen carefully to them and ask questions to the patients and these would answer to the questions feeling comfortable. There are questions you ask and the patient just answers “yes” or “no”; the best thing is to let the patient express themselves until they finish what they have to say, and the health care provider should not be distracted away meanwhile. For instance today we are obliged to switch off our mobile phones so if the patients talk when I am listening to my mobile phone, the patients will realize that I am not listening to them. That is something about which the health care provider should be careful, they should not be looking into a book when a patient is talking. They should not be looking pictures on whatsap either. I think that it would be better if the health care provider would listen to the patient, understand them and follow them up as they speak and especially pay attention to the patient’s reactions because they also help the health care provider to know how to help the patient. But when you tell the patient “Be quick? Be short, we are running short of time?” the patient understands that you don’t really have time to listen to them. Because this is a mistake that we usually make, we tell patients “speak quickly, I don’t have enough time.” Also, the health care provider talks on the telephone and you really find that we don’t have time to listen to patients. We feel that it’s just fine to prescribe medications, give the prescription to the patient and they go and get the medications while there are problems that would be solved through conversation.

**I:** What do you do when a patient cries?

**R:** Well, when a patient cries, you do not shout at them, you just let them cry. But you have to cool them down, and when their feelings have subsided away, you resume the conversation. Because a patient can cry because they remember their problems; such patients are so many, we have them. For example a woman whose husband has been maltreating her and giving her diseases multiple times, she cries before she tells you anything. You let her cry for a minute, that’s why in the consultation room there should be tissue papers to sweep her tears and after she has cooled down, you start the conversation.

**I:** Is it important to help patients control their feelings resulting from their illness?

**R:** Yes, it is really good to help them because when you do help them, it becomes the first step that shows them that you are together and that you really listened to their problem. It is very good, we sometimes receive people who have trauma and others who have various problems. So, when you help them to manage their feelings, they feel open and they tell you about their problems and they therefore return to their normal mood and they laugh, they recover as soon as possible, they do not keep their feelings if you show them that you are near them. Yeah, they are not overwhelmed by the feelings, they return to their usual mood so quickly. So, you give them the hope that their problem have an answer.

**I:** Is there something that you do to know if a patient understands or listens to you well what you are saying?

**R:** Well, the first thing that you do is to ask them repeat what you have agreed on, and they repeat it; sometimes they even tell you, “Write it down on a piece of paper and give it to me.” After they have repeated it, you are sure that they got it well and you also repeat it in order to emphasize it and make sure that they go with it. Sometimes they really ask you, “Write down all the things that I have to avoid on a piece of paper and give it to me” and then you write those things down and give them to the patient and they go. That shows us that the patient is also trying to be involved in the solution of their health problems.

**I:** Should a health care provider help patients to participate in the health care that they are given?

**R:** That point is very important because any health care that the patient did not get involved in cannot be successful because the problem reoccurs. As I said before, if for example it is a patient who regularly drinks very strong beers like liquors when they have hypertension and then the health care provider says to “Don’t drink beer, don’t drink such and such types of drinks” and that the patient doesn’t make a decision to give up those things, the patient surely has a relapse. For example we receive cases of those people who take medications for epilepsy, we deplore the fact that those people stop using the medications when they see that they have not collapsed for some time! But when you explain to them that stopping the medications will definitely make them collapse once again, they blame themselves when they collapse into the fireplace. We urge them to come and get other medications when they remain with the pills that they will only take for the next three days. Many patients understand that and they really come back to take other medications and you see that they respect the appointments. But there are patients who get medications for ninety days and go home and take them but they don’t come back to take other medications when they finish the ones they got; but we are the ones to be blamed for that because the health care provider who prescribes those medications does not explain them the consequences that will occur if they stop taking the medications.

**I:** So, what can a health care provider do to enable the patient to be involved in the health care that they are given?

**R:** In fact as I said, it required that you sit down and discuss and both of you become one person, I was going to say that you become the patient’s colleague but it’s not what I want to mean; what I am trying to say is that you have to become the patient’s adviser; the patient should not be a very superior person in a way that the patient is afraid to talk to them, no. Instead, the patient should see the health care provider as an adviser and the health care provider should let the patient tell them about their problems in a detailed way and therefore they share ideas, in that case it is good because the patient participates too in the treatment process.

**I:** Huh, do you think you have another information that you would like to add on the conversation that we have had?

**R:** Well, this conversation was good; thanks to it I came to realize that there are things that I have to amend. There are things that I said which happened to me and there is a lesson that I have learnt even before this conversation may have an outcome; but among what I said, there are things that I do and there are things that I don’t do which should be done and that myself I should be doing. So, what I want to add is that this program should be reemphasized in hospitals and health centers; people should not see us like people who just give medications and injections only, instead we should be seen as advisory centers. We know that there are challenges, people work under stress in addition to shortage of time but we should be able to give what I can call the minimum possible so that together with people we are able to find solutions to their problems. This is because if you conducted a survey, you would find that more than fifty percent of health care providers are the only ones to find solutions to people’s problems but it is necessary that people also get involved in the solution of their problems so that we have a long time solution for the illnesses. So, health care providers need to be trained in different subjects as I was saying, but this particular subject has been forgotten whereas it is very important.

**I:** At some point you mentioned “the minimum”, did you want to say “the minimum” or you wanted to say “the maximum” in order to achieve the objective? I don’t know if I got it well?

**R:** What can help you to achieve the objective – even if you may do little – I didn’t want to say the maximum because in that case you would have done all that is required; I wanted to mean the little things that can help you to achieve the goal, that can help you collaborate with the patient to find their solution. That’s what I wanted to say. By saying ‘the minimum” I wanted to mean the minimum things that can help both the patient and the health care provider to work together and find a solution the patient’s problem.

**I:** And then do you think that there are questions that we didn’t ask you which can help to improve the conversation between the health care provider and the patient?

**R:** Yeah, it’s that although people learn how to engage patients in conversation at school, it should be detailed. It’s true that people learn it at school but it should be emphasized in schools that train health care providers, nurses, medical doctors and midwives; this skill should be integrated in the curricula of health care providers. The course is considered like a minor course, and it is not among the major subjects; this lesson of communication skills is categorized among the ordinary unimportant subjects while pharmacology and gynecology are classified among very important subjects. The conversation is of the first importance because you treat the patient’s illness which is in their body, but you also treat their mind. Therefore considerable importance should be attached to the communication skills course like other subjects that deal with treatment of patients.

**I:** Thank you so much!
