# Supplementary material for: Providers’ perceptions of communication with patients in primary healthcare in Rwanda
Source: PLoS One. 2018 Apr 4;13(4):e0195269. doi: 10.1371/journal.pone.0195269 (PMC5884556; doi:10.1371/journal.pone.0195269)
Supplement: S1 Dataset — (ZIP) [file pone.0195269.s001.zip › S1 Dataset/PPC-Provider 4.docx]

**PPC-Provider 4**

I: Interviewer, R: Respondent

**I:** So, I would like to start by asking you what your name is. I didn’t ask you it. What is your name?

**R:** I am called [*name*].

**I:** Okay, um, can you tell us a little bit about the conversation that a patient has with the health care provider in the consultation room at the health center?

**R:** Well, what I can tell you about the conversation that a patient and the health care provider have at the health center, um, in fact the major purpose of the conversation is to know what the patient has come to do at the health center. You have to know “Did the patient come to seek health care?” or “Did the patient come to seek for a piece of advice?” because we play two roles at the health center: there are people who leave their homes in order to seek health care and there are people who leave their homes in order to seek pieces of advice. That’s what I can say that happens in the concultation room. The conversation that we have is about the patient’s illness or about the advice they are seeking.

**I:** Okay**.** What is the contribution of the conversation that you have with the patient in the consultation room to your work at the health center?

**R:** The first contribution that I can say is that it helps me to know the illness the patient has because we have a conversation and they tell me about their illness, and then I am able to know how I will help the patient. The conversation helps me to know the types of medical tests that I recommend for the patient, it also helps me to know the types of medications that I prescribe for them thanks to the examination that I have done. In addition, if it is someone who has come to seek for pieces of advice, I get to know what problem they are seeking advice for.

**I:** Tell us in full details what the best conversation between the health care provider and the patient looks like?

**R:** Well, the best conversation between the health care provider and the patient is held in such a way that the patient doesn’t hide some things from you. The first thing to do is to establish trust between the patient and you so they feel comfortable and tell you everything; because when a patient comes but they don’t feel comfortable, the conversation goes badly and the patient hides some things. For that reason, it requires that there is created trust between the patient and the health care provider; after the patient is feeling at ease, they become open and tell you everything.

**I:** Is it necessary that health care providers have enough skills in engaging patients in conversation?

**R:** Of course yes. It is necessary. Because if you don’t have enough skills in conversing with patients, patients come and feel uncomfortable and don’t tell you their problems, and when they are not comfortable, there is some information that you miss and as a result you treat part of their problems. For instance you may treat the problems you found from the tests, but you aren’t able to treat other problems the patient didn’t tell you because they were not comfortable with you as a result of not having engaged them in conversation properly.

**I:** Why do you need to converse with patients in a proper way?

**R:** I need to properly converse with patients who come to see me in order to be able to solve their problems. If they are ill, I must know how serious their illness is and therefore I am able to help them accordingly. If they also need a piece of advice, I have to know their problems so that I am able to give them the advice they need.

**I:** Do you think that having a good conversation with a patient can help you to improve the way you care for the patients?

**R:** Yes.

**I:** Why?

**R:** Having a good conversation with a patient can help me to improve the way I care for them because if they come and talk to me, for instance when they have a certain illness, I will be able to understand their illness and then I will be able to help them in a better way. In this regard I need to receive them well, care for them and talk to them so kindly that they explain to me their illness and in this way I will be able to help them as well as required. So, having a good conversation with the patient has a paramount importance because it enables me to know how to help them, which is basically my goal, so that their problem comes to an end. At that time, when the patient talks to me openly, I help them as well as needed.

**I:** What is the benefits of a good conversation that you have with patients?

**R:** The benefits are those things that I was saying; it’s that I will be able to help the patient as well as needed. That means, if they come and have a good conversation with me, they will return home after I have solved the problem that made them leave their home. It is possible that I may not solve the problem due to lack of ability, but at least I will have understood it because we have had a good conversation; therefore I will refer them to a high level health facility where they will be able to solve their problem.

**I:** Okay. Do you think that having enough skills in conversing with patients can enable you to help better the patients who come to see you?

**R:** Yes. Because even if I said that we help patients and that we have a good conversation, I will use the skills that I have. So, I will relate my skills to what we have talked about during the conversation. If I don’t have enough skills, I will not be able to help the patient in a better way. That is why it is necessary to have the skills.

**I:** Here I am talking specifically about the skills in conversing with patients, communication skills. So, do you think that having these communication skills can enable you to better help the patients who have come to see you?

**R:** Of course, yes. The reason why I say like this is that, you know, there are different people in the society where we live. Someone can come and tell you about their problem and you immediately take it for granted that it is just like that and you don’t even consider asking them some questions to understand the root causes of that problem and for how long they have had it. That is why it is necessary to have skills in conversing with patients, it is of a great importance. If you don’t have those skills, you cannot know the illness that someone has, the cause of it, for how long they have had it and whether or not it is inherited from their parents; you see, if you just take what the patient tells you as it is and you don’t go in deep to ask more questions, you cannot get to know all that information. And this also requires skills. So, you understand that if one doesn’t have the skills, they understand things superficially and as a result they fail to do the right things.

**I:** Did you ever receive a training that was aimed at helping you to improve the way you talk to patients who come to see you?

**R:** I didn’t receive that training. What I know is only what anyone learns at school, but I was never given any training that people get after getting a job, or the things that one is taught when they are doing their job, I mean after they reach on the field of work. It is my own initiative, I feel that I have to talk to a patient in a friendly way so that I get to know the information about their illness.

**I:** When you were at school, were you taught any courses that help you to improve the way you talk to patients who come to see you?

**R:** At school, we had a very short course, I cannot say that it was long enough. The course was taught a few hours and people did not attach any big importance to it. Such course about engaging patients in conversation are not given much importance. People attach a great importance to the courses that deal with what you will do once you get your jobs, they do not teach you how you will talk to patients.

**I:** Okay, what is the reason behind that?

**R:** Well, maybe people who designed to courses curricula did not care about this particular. Maybe they felt that it is essential to teach someone things they will do things at their job instead of teaching them how to talk to the people for whom they will do those things.

**I:** Is it useful to have such training?

**R:** It would be very important if it would be thought of. Even if people seem to ignore it, it is actually the foundation that helps you to better treat someone because you have had a good conversation. Therefore, if much effort is dedicated for the coversation and if great importance is attached to it, it would be very useful.

**I:** On a scale of 1 to 10 points, how would you rate your skills in conversing with patients in a proper way?

**R:** I would give myself like 7 points.

**I:** It’s obvious that you lack three points so that you score ten out of ten.

**R:** Yeah, it’s three points.

**I:** So, what is the problem that hinders your ability to effectively converse with patients who come to see you?

**R:** Well, there is a gap. We have different categories of illnesses. There are people who have chronic diseases which cannot be cured and which last for a long time and there are people who have illnesses that appear suddenly; so we need to know “How should I talk to a patient who has such and such type of illness?” Sometimes you take all illnesses in the same way, which is a problem.

**I:** So, that’s where you see a gap?

**R:** Yes, there is a gap.

**I:** What do you feel you need to improve?

**R:** Well, what I need to improve is just what I have said a while ago: to know how to talk to a patient who comes with a lifelong illness, to know how to talk to a patient who has an illness that will be cured within three months. What I need is to be able to know how I can talk to a patient in relation to the category of their illness, a particular way of talking to the patients with different illnesses instead of just talking to all patients in the same way.

**I:** You want to mean “Taking all illnesses in the same way?”

**R:** Huh, in general.

**I:** How does your collaboration with other health care providers make better your conversation with patients?

**R:** Collaboration?

**I:** Your collaboration with your colleagues, other health care providers. How does it play a role in making better the conversation that you have with patients?

**R:** The role that I would say it has, you know, in all health institutions, when you feel that there is something you don’t know well, it is necessary that you seek advice from your colleague. You can go and tell you colleague “I received a patient who has such and such a problem, he has had the illness for a given period of time, he has taken such and such medications”. So, you discuss all that. You see, in this case I am sharing with my colleague the conversation that I have had with the patient in order to work together and solve the patient’s problem; I need help from my colleague. So, I think the importance of that collaboration is to help me explain the patient’s problem to my colleague so that they can help me to solve it.

**I:** Well, maybe I have to ask it once again. You see, there is a way you collaborate with your colleagues, other health care providers. The collaboration that you have, does it contribute in any way to the betterment of the conversation that you have with patients? What is that contribution?

**R:** The contribution that it may have, if I understood the question well, it’s about the conversations that I have with my colleagues when we are at work. It happens that people share work experiences and then your colleague can tell you for example “When we receive a case like that one, we handle it in this way.” You understand that it is a conversation that I have with my colleague. So after that, I go and do some researches, I can go and search on internet and see if what my colleague has told me is true or not. So, my colleague can share with me an experience which can be useful for me I the future when I receive a patient with a similar case. So, collaboration is really essential.

**I:** Do you think that it can help you during the conversations that you have with patients?

**R:** Of course. It is very helpful.

**I:** Do you think that it is important to ask patients if they came to seek health care expecting something particular?

**R:** What did you say?

**I:** Is it important to ask patients if they have come to seek health care expecting something in particular?

**R:** Yes, of course. For me, I think that it is useful although it is not done most often. But is it useful because it would help you to know where you have a weakness. Because you can ask a patient “Do you hope that you will get better?” and they may answer you, “No. I have come here in a bid to avoid being laughed at by other people for not having sought health care”. In that case, you know where there is a problem and then you invest much effort accordingly. The patient would tell you what they feel about your services and then you know where to improve. So, it is of a central importance to ask patients what they hope to see after receiving health care , because they would tell you whether or not they trust that you will help them.

**I:** Okay, is there something that you do to know if patients would like to have more information about their health?

**R:** Yes.

**I:** What do you do?

**R:** You ask them questions. You ask them questions like “Did you previously have any other problem?” and then they tell you. You orient the patient because more often than not, patients tell you irrelevant things; so it is necessary that you orient them. So, when you ask them if there is something which they may have forgotten and if they previously have had another problem, all this helps you to get more information.

**I:** Okay, but there it’s you the health care provider who are asking questions to the patient in order to try to know some more information about the patient’s situation. Now, on the other hand, there is information that the patient needs to know. Is there anything that you do in order to know if the patient needs to have more information concerning their health?

**R:** Yes. Because you ask them, “Is there anything else you would like to ask me concerning your health?” and they can tell you openly. But when you do not ask them, they do don’t consider telling you about it, they just go. I really do that when I have a small number of patients to see. When I see that I have many patients, I do not ask them that question so that I save time to see many patients who are waiting.

**I:** Why do you it in case you decide to?

**R:** When I decide to do it, I do it in order to know the illness that the patient has. I can give you an example. Yesterday I received a patient who had malaria. I gave her the opportunity and I asked her that question. I told her, “Do you have any other thing to ask me concerning your health?” and she explained to me that she also used to have epilepsy and took medications and later on she stopped taking the medications. She said that people prayed for her and she got well. However, she reported that she had dizziness in the previous week and that the dizziness was similar to the one she used to feel when she had a crisis before. So, you understand that the opportunity I gave her enabled her to tell me about other health issues which were not the reason why she had come to seek out health care.

**I:** Do you think that it is necessary to explain to patients all things that they would like to know?

**R:** It’s very necessary.

**I:** Why is it necessary?

**R:** Explaining to them helps them to know how to care for their health or if they have a particular problem, they get to know how they have to behave according to their situation.

**I:** Is it important to let patients be involved in making decisions related to the health care that they would like to be given?

**R:** It is important. However, there is still a problem of understanding. I would say that my colleagues decide to make decisions for the patients, they have not understood this. They are the ones who make decisions about what is going to be done for the patient. There is still very low understanding of this, but for me I do understand it. I know it is very necessary.

**I:** Why do you feel that it is necessary?

**R:** I feel that it is very necessary because the health belongs to the patient even if I am going to help them. It is also because when the patient thinks that what I am going to do for them is likely to cause problems, they can tell me, “Well, this thing causes problems for me. I think we should do it like this”. So, if you don’t go carefully and do some analysis to see if what the patient would like cannot cause problems, there can be disagreement between you and the patient; and it is only a few health care providers who can do what patients wish. Health care providers usually do things for the patient in such a stubborn way that the patient cannot refuse that they do what they want. It is a very serious problem, and it is very difficult to make people understand, but they will understand slowly by slowly. But for me, I think it is very important that patients make a decision about what you are going to do for them; by doing so, the patient feels comfortable and confident that they have also played a role in the decision.

**I:** In case a patient would like to make a choice concerning health care, should the health care provider take the patient’s choice into consideration?

**R:** Yes. The health care provider should take the patient’s choice into consideration because the health care is going to be done for the patient. I think that the health care provider should consider it important as long as the choice cannot hinder the objective of the health care. If it is still possible to achieve the goal of curing the patient, for me I don’t see any reason for not considering the patient’s preference.

**I:** I would like an example for this, can you give me an example?

**R:** I can give you an example. I am going to give you an example that is related to the belief. I know this example for people who are called “Jehovah’s Witnesses”. Jehovah’s Witnesses do never accept to be given blood transfusion but there is another way you can do to save their life. However, at some point, especially at the hospital – I saw that case when I was at Ngarama hospital – someone came and said, “Do not give me blood transfusion” and it turned up necessary that health care providers made a decision by force. They gave the patient a blood transfusion and she recovered and she was discharged but you understand that implementing the patient’s preference was not a culture. I even remember that the doctor said “There is another way we can use to save your life, but I do not want to use it.” I was working there at the time. But you see that it is not acceptable. Another example that I can give you is about a woman who once delivered just there at Ngarama hospital.

**I:** Maybe that is an isolated experience, but it is not common. Is there an experience that you had, on your own?

**R:** Huh, my own experience is what I was going just to give you. We were assisting a woman in the delivery room and then the woman requested that we moved her from the delivery bed and put her to another bed which was there. The other bed was lower than the one on which she was lying. I remember that we refused. You see that it was something very simple. She said, “Could you please move me to that bed?” And for sure it was reported her young sister once fell down from the bed where she was lying and as a result she had a stillbirth. They had put her on that bed in order to follow her up but she had a stillbirth. That woman was seeing another lower bed which was there. She repeatedly asked, “ Could you please move me to that bed and let me deliver there?” My colleague refused that we move her. We were both caring for two women waiting to deliver. She (my colleague) refused, but it was something simple that we would have done for that woman so that things would go well.

**I:** Huh, okay. When a health care provider shows emotions, how can those emotions hinder the conversation that they are having with the patient?

**R:** Well, when the health care provider shows their emotions, maybe I don’t know if I understand what the term “emotions” means here; I don’t know if it means a particular way of understanding a problem of someone with whom you are conversing?

**I:** When I talk about emotions, I mean what is inside my heart; that is “emotions”, it’s “feelings” and you understand that they include happiness, anger, sadness. So, when the health care provider exhibits such emotions, in what ways do the emotions impact the conversation that they are having with the patient?

**R:** Well, showing your emotions has a great impact, it shows that you are understanding what the patient is telling you. That’s what I think and nothing else. If you talk to a patient while you are busy for instance using your mobile phone or doing other things and don’t really show them that you are following what they are telling you, they see that you are not caring for them.

**I:** Is it acceptable that health care providers show sadness or anger when they are with a patient?

**R:** I would say two things on that point. Firstly, I can say that it is acceptable for a health care provider to show their happiness or sadness when they are with a patient. However, we have to be careful. When do I say that we have to be careful? It is when you are comforting a patient. If someone comes suffering and crying and that you also cry, no-one will cool down the other. There should be someone who is emotionally stronger enough to be able to comfort the weak one. That is why there is a need of skills in managing the emotions, skills in how to talk to patients. I am telling you the truth, it is very rare that you can – suppose that someone has had very serious problems and as a result they have headache and then so they come to the health facility. You see, someone has experienced serious problems, they consequently have headache and they come to the health facility and they tell you about their problems. It is very rare that the health care provider puts themselves in the patient’s place first of all so that they can to understand the patient’s problems; that’s when the emotions would appear. The patient understands that you are actively listening to them and that you take what they are saying in a serious way. If I step backwards a little bit, concerning the fact that you can show the emotions to the patient in two ways, if someone is overwhelmed by their emotions and that you also feel seized by the emotions, the situation becomes worse. That is why there is a need of skills about how one should talk to patients. People should know which way is suitable to talk to such type of patients, and I tell you the truth, this skill is nonexistent, really it is.

**I:** There you were talking about the emotions but you kind of focused on the sadness or the fear that the health care providers can have upon listening to the patient’s problem, if I got you correctly. However, can a health care provider exhibit happiness when they are with a patient?

**R:** Of course yes, and it is even recommended.

**I:** Why?

**R:** In the work that we do, that is very recommended because I think that even the society takes us like peaceful people and peace shows through happiness. So for me, I take this as a pre-requisite quality in the work that we do at the health facility. It is difficult to make someone feel open to you and tell you their problems if you do not show that you are sorry for them.

**I:** Do you think that patients can be worried about telling their health problems to the health care providers?

**R:** Come again?

**I:** Do you think that patients can feel worried about telling their problems to the health care providers?

**R:** Yes.

**I:** Can you explain more?

**R:** Patients can feel afraid of sharing or telling their health issues to health care providers because of, in my opinion, the way they see them. They can see that you are an angry person. Patients cannot tell you their problems if they see that you are angry, unsociable and unfriendly towards other people, they feel that they cannot talk to you. If patients know that you are weak in the society where you live, they can fear to tell you about their illness, especially about their health. That’s what I think.

**I:** What can be done?

**R:** I think that what can be done is to talk to the health care providers about how they should behave. There should be prepared a curriculum that talks about how they have to behave.

**I:** When should they behave in a particular way?

**R:** They should have a peaceful behavior before patients, when they are with patients and even when they are in the community.

**I:** What is your experience conversing with patients whom you think have low level of education, especially those who are illiterate?

R: What I experience as challenges?

I: Yes

**R:** I have so much experience when I care for people who have low level of knowledge. Because the first experience that I encounter with is that they refer to any illness as tapeworm, even one who has headache comes and say, “I have headache but it is a tapeworm which has moved into my head”, even when they have en aye illness, they come and say, “A tapeworm entered into my eye.” There is a problem of low level of knowledge which makes them unable to know what illness they have; they cannot do even a slight analysis or figure out the type of illness they have. They always confuse things. Another issue arising from their low level of knowledge is that they do not know how to explain their illness. So, you have to go slowly with them as they describe their illness and in the end you get to understand well the real illness. It’s different from people who are knowledgeable. These ones come and tell you straight how they are feeling and when the problem started; they know how to describe their problems.

**I:** How do apply your communication skills to converse with patients of that category?

**R:** Well, the way I apply my communication skills to converse with patients, first of all I do respect what they tell me. I show them that I respect what they say and that I am taking it into consideration, but I go slowly so that I retrieve what I need from what they are saying. The first thing that I do is to respect what they say and do not blame them or shout at them; I just listen to them and talk to them so that I can reach to what I want.

**I:** How does the Rwandan culture influence the conversations that a patient has with the health care provider?

**R:** Well, the culture plays a great role in the conversations that a patient has with the health care provider because there are many things linked to the culture that patients say. There are things that have to be respected at the health facility. They use their mindset in order to abide by the culture. I can give you an example: we recently received an old woman who had an external prolapse of the uterus. Her uterus seemed to have slid out. We struggled to make her reveal to us that problem. We wouldn’t have known the real problem if someone did not talk to her in a Rwandan way by beating about the bush; that is the culture of not immediately say something openly.

**I:** If I got you well, you said that Rwandans do not like to speak openly, they don’t say things in a direct way. So, this culture influences patients in some way to not directly explain their problems?

**R:** That is what I wanted to say. Another thing that I want to say is that people take illness for culturally known problems, or problems which are linked to our culture. Someone comes and tell you, ‘You know what? This patient is a victim of witchcraft.” This happens in this community. Someone tells you, “This patient has headache but he had been to his grandmother some days ago. When he had fever, I thought that he was victim of some rituals but when I saw that fever persisted, I realized that I was wrong and now I bring him here, but I first thought that he was victim of that.” So the caregiver tells you about the old practices of worshiping ancestors. They confirm to you, “It happens most often especially to adult people.” It doesn’t happen to young people but for adult people, they tell you that.

**I:** How does that influence the conversation that you have with patients?

**R:** The first influence if that they explain to me their illness, then I get to understand for how long the patient has had it and I also get to know whether or not they had taken any medications at home and I understand the reality about the illness.

**I:** In your opinion, what usually hinders the conversations on the side of the patient?

**R:** Well, the reasons why the conversation does not go smoothly are the working conditions. More often than not you will find that there are a few staff members in health institutions. So, when there are a few staff, it becomes necessary that…[interrupted]

**I:** Here I want to lay an emphasis on the challenges, or factors that can impede the conversation but arising from the side of the patient.

**R:** The reason coming from the patient’s side? The first challenge is low level of knowledge of the person who talks to the patient.

**I:** There you are talking about the health care provider, aren’t you?

**R:** Aha, you are talking about the patient?

**I:** Yes, I am talking about the patient. There can be things that hinder the conversation that you have with the patient and those factors can come from you the health care provider, they can arise from the work conditions as you have said. Now I want to talk about the factors that arise from the side of the patient.

**R:** On the side of the patient, what I want to say is that there is a factor which can negatively affect the conversation. That is ignorance or not the fact that one does not know how to explain how they are feeling. That is the very first factor. This makes it that you treat the patient in an improper way. But if someone is able to explain well how they are feeling, you try your best. That is the first factor. Another factor which can make the patient to not explain well how they are feeling is that they can arrive here in the morning at 7.00a.m. and you examine them at 3.00pm. The patient at this time is so bored that they do not explain well their illness.

**I:** It makes the conversation between them and you go in a bad way?

**R:** Yes, it doesn’t go well.

**I:** Now, what are the factors that usually affect negatively the conversation on the side of the health care provider?

**R:** On the side of the health care provider, the factor is the same as what I was saying before; it is the lack of skills in engaging patients in conversation. That is factor number one. The second factor is the big number of patients who are there. Because the more patients there are, the shorter the conversation the health care provider will have with each patient.

**I:** Are there other factors on the side of the health care providers that can hamper the conversation with the patients?

**R:** Another thing that can negatively affect the conversation between the health care provider and the patient is that the health care provider may have their own problems, personal problems. They may have personal problems that are so serious that they are unable to converse with patients. For example the health care provider may have problems at home; if they have problems at home, the conversation will definitely not go smoothly. The health care provider may also have been reprimanded by their superior early in the morning before starting the job and therefore they won’t do the job in a good way. They will not do the job calmly.

**I:** So, on the side of the work conditions, what are the factors that usually hamper the conversation between the health care provider and the patients?

**R:** Factors which can cause what?

**I:** Which can make the conversations between the health care provider and the patient go in a bad way, as a result of internal conditions at the health center?

**R:** Well, for the side of the health center, it is what I was going to say a while ago; there are some titulaires who, I would say, they do not know how to manage their staff. Titulaire who does not know how to – suppose that we are in the staff meeting and suddenly the titulaire points at someone and shouts at them, “Such and such thing that you did, if you do it once again, you will have problems with me. I warn you in public.” This is a small example I give you. So, after shouting at the employee like that, you send them in the consultation room. I am telling you the truth, that employee will not be productive on that day. As a titulaire, you should have invited the employee in your office and warn them for example saying, “You haven’t done well in such and such circumstances” and warn them in a friendly way. So, such management of staff at some institutions hinders the conversations that the staff have with patients. Another factor is about equipment that people need at work on daily basis. When the equipment is not supplied and distributed, employees do not have a good environment to work in. There should be a good environment at the health center and even the management of the staff should be good in such a way that the leadership encourages the staff to like their jobs in general. I think this is a responsibility of titulaires of health centers.

**I:** If I come back a little bit on the equipment that you said, you wanted to mean materials which can prevent the health care provider from having a good conversation with the patients?

**R:** Someone can come and tell me for example “I feel dizziness” – suppose that it is an adult person – if I ask them for how long they have had headache they can say for instance “I have had it for weeks”, it’s an example, or “I have had it for five months.” So, I can ask myself, ‘Why should I talk to this person when I don’t have blood pressure monitor?” Therefore you hurry up and discharge the patient because you know you will not be able to do anything further in relation to what there are telling you and that you don’t have any piece of equipment to use.

**I:** What do you do in that case?

**R:** In that case there is nothing you can do; you just give the patient what I can call “a small nothing” and in that time, the mistake is for your superiors because you report to them about the lack of equipment but they don’t solved it.

**I:** Can you give me example of things which you find difficult to tell patients about?

**R:** Yes, there are things which are hard to explain to patients. Firstly, it is things related to illnesses that affect reproductive organs. We most of the time have difficulty telling people about these issues. Another things which is difficult is to talk to patients who have incurable diseases. Those incurable diseases, especially people who have HIV/AIDS and people who have diabetes, it is really difficult to talk to these patients because usually they have lost hope. It requires that you are someone who are skilled enough to talk to them. Although it is difficult, some of us do not have those skills. We talk to those patients in an ordinary way. But people who have those categories of diseases are difficult to talk to.

**I:** What do you do when the situation is like that?

**R:** I try to talk to them and reassure them, that is the first thing that I do. I show them that in this life, what has happened to them can happen to us tomorrow. In a few words, I try to comfort anyone who has such problems or I try to put them in the mood of the conversation so that they are able to understand me.

**I:** In your work, did you ever receive patients with whom it was difficult to talk to because of a certain problem? What was the problem?

**R:** Well, I received an eighteen-year-old girl student whom I asked to have a test and I found that she was HIV positive. I had difficulty explaining that issue to her, and I struggled until nearly two hours. Imagine spending two hours with a patient when other patients are waiting! I explained to her, I beat about the bush in order to be able to tell her about her test results and in the end I openly told her the truth and she cried. Everything that I said, she cried. You understand that I spent two hours with that girls, it was a serious problem. Luckily, I continued to explain to her and finally she understood me, but it required that I use another efficient strategy. I went to bring her whatsit – I went to bring a small video about how to care for children who are HIV positive. It was on my computer; I got it when I had a training on that subject. It was a ten minute video that I had on my computer. I brought it and showed it to her, and it helped her very well. But it wouldn’t have been easy for me to explain to her if I didn’t have the video.

**I:** Did you receive a patient with whom it was difficult to talk to because of their mental illness?

**R:** Yes.

**I:** What did you do?

**R:** So, mentally ill people are difficult to handle. I didn’t have knowledge about how to talk to a mentally ill person. I had to accept everything that he told me in order to care for him. I agreed with whatever he told me but when I saw that we were going to do what he said, I refused and proposed to change it and do something different and by doing so I got rid of that particular thing from his mind and therefore I made him agree with what I proposed. So, we worked like that and he continued to tell me other things. But it is a fact, I received a mentally ill person and I struggled to deal with him.

**I:** Did you receive a patient with whom it was difficult to talk to because of their problems of deafness, dumbness or blindness?

**R:** Yes, I receive those ones every day.

**I:** What do you do in that case?

**R:** In that case you resort to their caregiver so you are able to discover what the patients is saying. Like a person who lives with the patient. It becomes a problem when the patient has come alone; in that case you use signs.

I: And then, did you receive a patient with whom it was difficult to talk to because they had a difficult personality?

R: Yes.

**I:** How did you manage the situation?

**R:** Yeah, there are people whose physical nature – for example I once received a patient who only moved using his hands. You understand that he came and sat down in the consultation room. You cannot see him when you are on a chair. So I had to stand up and squatted near him. I examined him in squatting position in order that I was as tall as he was. You understand that it is something which was difficult for me. All his legs were amputated and then it turned up necessary that I also squatted near him. I moved my chair aside and I squatted and we talked.

**I:** That was the physical nature that you were talking about. Now, I am talking about the patient’s difficult personality, did you receive anyone who was like that?

**R:** I received that case also. It was a person who was very harsh. Whatever you could tell him, he would just answer you ‘Do this for me and then I go home. Treat me and let me go home.” You understand that he was unmanageable.

**I:** How did you handle the situation?

**R:** It means that he was very angry to a high extent; it became necessary that I cooled down and became like his servant. I listened to whatever he told me. He talked to me angrily, and I told him friendly. I did what he asked me to do for him and he went back home. I remember that I later on crossed paths with him in Muhanga town, just here. He greeted me and he explained to me the problem he had had when he came to the health center. He explained to me that he had broken relationship with his wife after having not been living together for nearly two months. That is the case he had had that made him that angry. I cared for him properly, I calmed myself down and I successfully dealt with him.

**I:** Is it necessary that you tell a patient about a problem which you think they probably have?

**R:** Yes, it is necessary.

**I:** Why?

**R:** Well, you would have done nothing if a patient came and told you about their problem and you discuss it but you don’t tell them likely problems that can result from their illness. So, it is necessary that you sit down and discuss everything about their illness.

**I:** What can you tell a patient when you are unable to identify their problem?

**R:** Well, what I would tell a patient if I was unable to identify their problem, I think it would not hide the whole truth and at the same time I would not tell them the whole truth. I would work on both sides: I would not show him my weakness but I would not also hide from him the fact that I wasn’t able to solve their problem.

**I:** Then, what would you tell them?

**R:** Well, what I would tell them, um, I would explain to them the problem that they have and that I am not able to handle it successfully but I would like to refer them somewhere else where they can be given more advanced help. Because we are at the health center and we have the district hospital. If the district hospital is incompetent, they can refer the patient to the referral hospitals. So, wherever I can be, if I fail to deal with a certain issue, there is always a higher health facility where I can refer the patients.

**I:** Concerning the medications that the health care provider prescribes for the patients, do you think it is necessary to explain to the patients the type of medication you prescribe for them, how it works and any possible side effects that it can have?

**R:** It is very important because if you do not explain all that information to the patient, sometimes they go and use the medication and experience side effects but they think that it is a new problem that they have and therefore they return to the health center. But when you have explained well all this to them and that they later on experience side effects, they hang on in there. They try to put up with the side effects or they can even come and report it but having not been worried. They come and say “I had been told this could happen to me.” So, it is necessary to explain to the patient what may or not happen to them as they use the medication because if you don’t explain to them, they can even take much of the medication at a go. It is very important to explain to them so that they say “I am going to seek help for the side effects that are caused by the medication” because you have already explained it to them. When they ever experience the side effects, they don’t get surprised or worried about them, they hold on if you have explained everything to them before.

**I:** Some of patients in Rwanda think that they do not receive enough information about medications. Is that true?

**R:** Yes, it is true. It is true because because there are so many patients that health care providers or people who give medications to the patients do not have enough time to explain to them very well how they will use the medications and likely side effects as well as how much of it to take at a go. That is a fact. Patients are not given all those explanations. It is rare that they can be given those explanations. Those who get those explanations are those who are knowledgeable and who have the courage to ask the health care provider how to use the medications. That should be discussed during the conversations that the health care provider has with patients. A patient can have the courage to ask, “Will this medication have side effects?” Only knowledgeable people have the courage to ask such questions, and these are very few.

**I:** Do some of your work conditions or situations at work hamper the conversation that you have with patients?

**R:** Yes.

**I:** Can you explain more?

**R:** For example, there are places where they put benches for patients near the window of the consultation room. You understand that the patient who is in the consultation room will not be open and talk about their illness because they will be worried that other patients queuing near the window might overhear what they say. The patient can evade to explain well about their illness because they see that there is no secrecy there. That is an issue of environment that can prevent the patient from feeling comfortable enough. Usually, it is because of the environment in which we work; it is like that.

**I:** [What](file:///C:\what) do you do if a patient requests to be referred to the hospital when you think that it is not necessary?

**R:** Well, what I do when I think it is not necessary, I explain to them that the medications that I give them will help them. This is a very common issue today. Someone leaves their home with the intention of just asking for a transfer simply because they have put in their mind that they absolutely need a transfer and yet when you examine them you see that it is an illness that can be cured on the level of the health center. So, it requires serious conversations so that you try to convince them that the medications you give them will help them successfully and then they go and use the them. But what we do is to explain to the patient that they will get better if they go and take the medications. They understand when we explain it to them.

**I:** Are there any challenges that are associated with having a conversation about health in Kinyarwanda?

R: Sorry?

I: Do you see any problems that are caused by the fact that you talk about health in Kinyarwanda?

**R:** Problems are there. You touched on an issue of reproductive health. Nobody can come and tell you how their sexual organ is in its real name. If it is someone who studied, they say it in English or in French. You understand that they change a language because their sexual organ is sick. And then, when it comes to an old woman who has not studied, she just say “Far down there”. What does she want to mean? “Far down there” refers to the vagina, but she cannot openly say the name of the organ. People usually evade to openly talk about their sexual organs.

**I:** The fact that at school you were taught in French or in English, is it a problem for you when it comes to talking to patients in Kinyarwanda?

**R:** Well, I don’t think it is a problem. There is no problem for me. We have our own mother tongue that we use in our daily life irrespective of the language in which we were instructed at school. Therefore there is no problem, you can change and talk our mother tongue.

**I:** What do you do when you have to explain medical terms that are in French or in English but they don’t have their equivalents in Kinyarwanda?

**R:** It is something very difficult. It I very difficult to explain terms which do not have equivalents in Kinyarwanda. But in any case, those terms refer to an illness and the illness affects a particular organ. So, you refer to the organ wich is affected to explain the term which does not have its equivalent in Kinyarwanda.

**I:** Can you give an example?

**R:** The example that I can give, for instance the example of, let’s say, um, I don’t find an example. Let me think about it, um, maybe cancer of the prostate. It is very hard to find its equivalent in Kinyarwanda, and I don’t think I know its equivalent; but one can explain it going from the organ that the illness usually affects. You can tell the patient “Cancer of the prostate has such and such signs.” You just explain it basing on the organ that the illness usually affects, because you cannot find how to say it in Kinyarwanda. Another example is hernia. Also, it is very difficult to explain hernia. Some people try to explain it in Kinyarwanda, but you try and say “It happens like this and this…” and patients understand.

**I:** Do you also encounter with some other problems that we haven’t discussed so far?

**R:** Problems are there. We experience many problems. Sometimes someone comes to seek health care and tell you about their life in the community. They can say for example, “My husband has insulted me” and things like that, and in the end you see that what they tell you has nothing to do with the illness they have. Another thing which is very challenging is to deal with patients who have chronic diseases. They lose hope for the future and don’t want to seek out health care. They come and tell you “Why should I continue to seek out health care when I will not be alive tomorrow?” It requires us to use much effort in order to convince them about the importance of seeking out health care. Such patients have usually become bored of their life; it’s a huge problem.

**I:** What can be done to improve your skills in conversing with patients?

**R:** What can be done to improve my skills in conversing with patients is that I should be given training about how one can talk to patients. It’s just training. It would be very wonderful to have it, my skills would increase. One should recognize the importance of the conversation that one has with patients because it is basically the foundation of the successful treatment.

**I:** Patients are different, and if they are different, they also express themselves differently. So, how do you apply your communication skills to adapt yourself to different communication styles of patients?

R: Come again?

I: How do you use your communication skills to handle various communication styles of patients?

**R:** Applying my communication skills to deal with communication styles of patients is to listen to them and give importance to what they are telling me. Even if it’s someone who is angry, I just listen to them although they talk to me angrily. That helps me to be able to help them. The one thing that helps me to achieve that is to only listen to them and don’t stop them from talking to me. I let them talk and I understand what they are telling me.

**I:** What can be done so that the health care provider helps patients feel more comfortable when they are conversing during consultation?

**R:** Well, what can happen so that the health care provider helps patients is first of all to give them time, listen to them and let them express themselves because, there is something that usually happens:when someone has many patients to see, they tell patients, “Stop by there, I understood.” But maybe the patients was going to say something else and you stop them from saying it. It would be very useful if one would give enough time to patients and listen to them. Enough time would enable patients to say everything and all this would help the health care provider to know more about the illness. However, this is very difficult to be achieved because there are a few staff but patients are many.

**I:** What do you do when a patient cries?

**R:** When a patient cries, I let them cry. When a patient cries, I try my best to make sure that no-one else comes in the consultation room when the patient is crying. If a patient cries, I close the door and sit in the consultation room and wait until they have finished crying and then after crying I try to cool them down and bring them back in their normal mood so that they are able to talk to me. But I don’t shout at them “Go out of my office”; I let them cry first.

**I:** Is it useful to ask patients to control their emotions like those ones which are caused by their illness?

**R:** Well, I think it is good to help someone manage the emotions resulting from their illness because otherwise, I won’t have been useful for them if I don’t help them calm down their emotions. I think that I have to give them advice, tell them about their illness and tell them that it sometimes happens that one has sufferings but emphasize that after suffering life continues. That is the truth for me, I cannot help educating someone about how they can manage emotions that result from their illness. I think that I would teach them how to control those emotions but I wouldn’t expect to prevent them to feel the emotions because it is very difficult.

**I:** Is there anything that you do to know if patients understand what you say?

**R:** So, what I do to ensure that the patient has understood what I am saying is that I ask them something and they give me a relevant answer. This gives me the assurance that they are getting what I am telling them. Additionally, if I ask them to repeat what I told them, they repeat it correctly. For instance I can ask them, “How will you use the medications?” and they start explaining to me like “You said that I have to use it like this”, from there I am hopeful that they are understanding what I am telling them.

**I:** Why do you do that?

**R:** I do it to make sure that the patient will abide by what I told them when we are no longer together; that’s why I do it.

**I:** Should a health care provider help patients to be involved in the health care that they are given?

**R:** Yeah, the health care provider should help them.

***I:*** *He should help them to participate in the health care that they are given?*

*R: Yes.*

**I:** How should he do it and why?

**R:** He should do it, he should let patients play a role in order to encourage them to prevent the illness from reoccurring and to protect themselvesagainst it. If someone comes to seek health care for malaria and you just give them medications but you don’t tell them how they can protect themselves against it, it is not good. You have to give them medications and also tell them to sleep in a mosquito net, to close doors in the evening, to remove any stagnant water around the house; that is what I think one should do. In that case the patient plays a role in the health care that they are going to be given. You give them medications and they will additionally protect themselves in the future.

**I:** Is there anything that you would like to add to this conversation that we have had?

**R:** Well, what I can say about this conversation that we have had concerning how to converse with patients is that so many people don’t grasp the importance of it to the extent that conversations between patients and health care providers are too short. They are too short, I have to repeat this. This is because people don’t recognize the importance of the conversation that patients should have with health care providers, or maybe they recognize the importance of that conversation but they encounter with the challenge of the big number of patients. So, I would suggest that there is organized a way of encouraging staff who work at health facilities, I mean health care providers, to give special importance to the conversation that they have with patients because it is the only way that one can understand well the illness that patients have. That’s what I think I can add on the conversation we have had. All people should know it. You have discussed it with me [*name*], but there are many of my colleagues who don’t care about it. There are those who would even tell patients*, “Just say what you have to say and then I give you medications and you leave here? There are many patients outside, I won’t be able to see them all if you tell me all those details.”* So, those are the things that we should be aware of as health care providers and therefore we should give importance to the conversations that we hold with patients. I don’t know if it is the government or whoever should do it but I think it is needed so that all staff who work at the health facilities recognize the importance of the conversation that health care providers have with the patients. They should know that the conversation clears the way for better understanding the patient’s illness which they are going to treat. That’s what I think I can add.

**I:** Do you think there are other questions that we should ask that can help to improve the conversations between the health care provider and patients?

**R:** I think what you asked are enough, they are so many. I don’t think you forgot something that you should have asked about the conversation that happens between health care providers and patients. Everything is included.

**I:** [*Name*] Thank you!

**R:** Thank you!
