# Supplementary material for: Providers’ perceptions of communication with patients in primary healthcare in Rwanda
Source: PLoS One. 2018 Apr 4;13(4):e0195269. doi: 10.1371/journal.pone.0195269 (PMC5884556; doi:10.1371/journal.pone.0195269)
Supplement: S1 Dataset — (ZIP) [file pone.0195269.s001.zip › S1 Dataset/PPC-Provider 8.docx]

**PPC-Provider 8**

I: Interviewer, R: Respondent

**I:** So, let us start our conversation. As I told you before, our conversation will be centred on the conversation that you have with patients when you are in the consultation room at the health centre. I would like to start by asking you, “Can you tell us a little bit about the conversation that a healthcare provider holds with patients when they are in the consultation room at the health centre?”

**R:** The conversation that a health care provider holds with a patient [*I: in the consultation room*] is a conversation that aims at knowing where the patient is coming from and also to know any health issues that the patient may have. All that information helps the healthcare provider to know well who the patient is and how to better treat them.

**I:** What is the contribution of the conversation that a healthcare provider holds with patients to the work that you do in the consultation room at the health centre?

**R:** The contribution of the conversation is that, first of all, the healthcare provider does not know the next patient who comes to see them; so the conversation helps the healthcare provider to know about the patient, how to help them and treat them or give them advice. It is thanks to this conversation that all this is possible. I think that the conversation is the foundation for the best treatment of the patient and for the best interaction between the patient and the healthcare provider; the conversation also is an opportunity for the patient to know who the healthcare provider is and vice versa.

**I:** Tell us in full details what you think is the best conversation between the healthcare provider and the patient?

**R:** Well, in my opinion I think the best conversation is the one that gathers both a healthcare provider and a patient in such a way that the healthcare provider is able to treat the patient successfully and without causing any conflict between them. Additionally, the conversation should not let the patient take the healthcare provider as someone who wronged them or a cruel person but instead it should be something that unites them and makes the patient have a need to go to the health facility thanks to the good care that they were given when they last visited the health facility and also because the healthcare they were given helped them to get better and to interact with the healthcare provider and to be acquainted to the healthcare provider.

**I:** Is it necessary that healthcare providers have skills in conversing with patients?

**R:** Yes, it is necessary.

**I:** Why is it necessary?

**R:** Because you find that the way healthcare providers care for patients is not the same due to lack of skills. Secondly there are healthcare providers whose behaviors are so strange that patients do not feel satisfied or they do not feel comfortable with them. They also need modern skills because I think things keep changing in medical domain whereas they still have outdated knowledge; so they need to have updated skills in caring for patients.

**I:** Here you are laying an emphasis on the skills in healthcare delivery in general, but let us come back to the skills related to engaging patients in conversation. Is it necessary that healthcare providers have this skill?

**R:** Yes, it is necessary that they have it.

**I:** Why do you need to talk to patients in a good way?

**R:** Of course I need to talk to patients who come to see me in an appropriate way so that we have a good interaction, so that I know them well and vice versa, so that both of us are comfortable talking to each other and that the healthcare provider can see that the patient is so pitiful that they become sympathetic and put themselves in patient’s place to better feel their pains because what the patient is experiencing can also happen to the healthcare provider one day. The healthcare provider should manage to not let the patient see that they are not sympathetic for them and that they intend to harm them; they should instead try to put themselves in patient’s place. It is necessary that the healthcare provider does this.

**I:** Do you think that the best conversation that you hold with patients can help you to improve the way you care for patients?

**R:** Yes.

**I:** How?

**R:** The best conversation that I may have with a patient can help me to do well my job because when we have a good conversation, the patient does not hide anything from me and therefore I cannot do anything superficially. This helps me to know well the person that I am treating, I am able to know both the inside person and the outside person and therefore I cannot treat the outside person because the inside person is also needed so much. When they hide the inside person from me, I can treat the outside person whereas the inside person might be the root cause of the outside problems.

**I:** When you talk about the inside person and the outside person, can you explain these terms in easy to understand words?

**R:** Well, it is very difficult to explain it in Kinyarwanda but it is philosophical, I don’t know.

**I:** You want to mean psychological?

**R:** Yes, psychological. Because if a patient comes complaining of stomachache, I can focus on the pains and give them medications for stomach pains whereas the root cause of the stomach pains is their thoughts or the problems that they experienced. I can therefore treat stomach pains without knowing what caused them. So, when I have had a conversation with a patient, I know the root cause of their problem and therefore I can provide them with an appropriate medication.

**I:** You are right. What are the benefits of the best conversation that you have with patients?

**R:** There are many benefits. It helps me to treat patients; as I said before, we can look at a person in two ways: there is the inside person that we can call psychological person and there is the outside person that we can call physical person, that means their physical appearance, their body, the way I can see them and see their pains; for instance if they have a wound I can see it. However, someone may come complaining of headache and the headache being caused by a great sorrow that they have been experiencing. So I can give them pills for headache but in reality I have not treated the root cause of the headache. Therefore, a good conversation helps the healthcare provider to not treat the patient’s problem superficially and it also help the patient to understand that the healthcare provider is a human being because some patients think that healthcare providers can read their minds; they think that the healthcare provider is able to know that they have headache because of lots of thoughts. Usually, the good conversation is a channel through which patients see that healthcare providers collaborate with God because they are able to uncover more information about the patient’s problems, even the ones that they did not say. It helps the patient to build trust in the healthcare provider.

**I:** Do you think that having enough skills in conversing with patients can help you to improve the healthcare that you give to patients?

**R:** Yes.

**I:** How?

**R:** Can you repeat the question for me?

**I:** I was asking if enough skills in conversing with patients can help you to better care for the patients who come to see you.

**R:** Yes. Enough skills help me to help patients who come to see me in many ways. If I had enough skills, it means that I would also be able to engage patients in conversation successfully. I will be able to orient the conversation, and if a patient wants to deviate it in a bad direction, I will be able to redress it in a good direction. For example if an angry patient comes in, I will remain so kind that they will talk to me in a friendly way once they see that I am also friendly to them. In addition, for the patients who come with false belief that healthcare providers are harsh and evil people, I will be able to calm them down and then we will be able to interact and they will tell me things that they would not want to tell me.

**I:** Did you receive any courses or training that aimed at helping you to improve the way you talk to patients who come to see you?

**R:** Yes.

**I:** Which ones?

**R:** I had those courses at school, and I also received such training.

**I:** Did you receive any training related to communication skills? Which aimed at improving your communication skills ?

**R:** No. It’s only at school that I had a course which was entitled “communication skills.” There is another formal training that I was talking about: I studied about psychology, I am a psychologist even if I work here as a nurse. So, in all those circumstances I learnt about the ways one can talk to patients.

**I:** Do you think it is useful?

**R:** Yes.

**I:** Why is it useful?

**R:** It is necessary that the healthcare provider has those skills because the healthcare provider’s objective is to appear kind to the patient, to give a good welcome to the patient and to treat them well depending on the way that the patient talked to the healthcare provider.

**I:** On a scale of 1 to 10 points, how would you rate your skills in conversing with patients in a suitable way?

**R:** On a scale of what?

**I:** On a scale of 1 to 10 points…[interrupted]

**R:** 1 to 10?

**I:** Huh [yes]

**R:** I would give myself like 5 points.

**I:** It’s five. You understand that if it is five out of ten, you see that it is half.

**R:** Yes

**I:** So, where do you have a gap in your communication skills so that you are not able to talk to patients in a suitable way?

**R:** I once realized that I had a gap in conversing with patients and then I went to school. Now I have not yet finished studying, that is why I gave myself 5 points out of ten. It’s because I have not yet finished studying; when I finish, I think I will have like eight points out of ten.

**I:** But you can specify and say “I have a gap in such and such areas” in my skills related to holding a conversation with patients?

**R:** I think many trainings should be given to healthcare providers so that they are able to converse with patients. The trainings would be in form of discussions and videos about how a healthcare provider should talk to a patient. Those discussions can be designed for healthcare providers including myself; so this would help me to improve the way I talk to patients. I feel that I don’t have comprehensive skills, I feel that I am lacking in this subject.

**I:** So, what do you need to improve?

**R:** I think that if that particular course was provided, those who would study it would teach others and I hope I would benefit from it. I am speaking on my own behalf, I am not talking on behalf of others.

**I:** Of course that is what I am also going to ask you. The way you explained it was general. Where do you have a gap in your skills related to conversation with patients? Is there something particular that you would like to improve?

**R:** Yes.

**I:** What is it?

**R:** I think it is what I have explained to you; if I remember well I told you that if I had the skills, if I learnt about it in a detailed way, there are some habits that I would correct that I have been having. There is a way you talk to a patient and when you ask them a question, you use a violent tone because it is the only way you know. But if you knew other ways in which you would ask them the same questions – I want to say that there are two forms of questioning: there is an open question and an open-ended question. These are types of questioning that I have known very recently, but I haven’t been using them. I think that I have little skill, if I had more skills in conversing with patients, it would be helpful for me. Because in the consultation room you can for example ask someone “Who is your father?” while in their whole life they never knew who their father is and consequently they suddenly start crying and after crying you become frustrated even if you know there is nothing bad that you have asked them. So, you can start asking yourself how you will resume the conversation after they have finished crying, you don’t know where to start in order to bring them back in the mood of the conversation and at the same time you don’t have a clue about what made them cry. You hurt them unawares because they don’t have their parent, but if you knew another way that wouldn’t hurt them, it would be very helpful. That is an example that I give you.

**I:** Thank you so much! How does your collaboration with other healthcare providers contribute to the improvement of the conversation that you have with patients? The collaboration between your colleagues and you, how does it play a role in making better the conversation that you have with patients?

**R:** I would say that the collaboration between my colleagues and I causes – I can give you an example – I can wake up today feeling irritable and then share it with my colleague so they can help me. For instance if I was to work in consultation on that day, I would request my friend that we switch work so I can work in another service depending on how I am feeling. It can happen that my colleague refuses that we switch work and therefore I can be forced to stay in my assigned service while I examined myself and found out that I should not work there on that day because I cannot talk to patients properly. So, when my colleague refuses, I can hurt a patient. Therefore, I think such collaboration is not good and it doesn’t allow us to have a good relationship. I may have a challenge of receiving patients whereas I had seen that I should not work in the consultation service.

**I:** Yes, it is true, but you talked about the negative side. Let’s now come to the positive side. Your collaboration with other colleagues, healthcare providers, can it influence the conversation that you have with patients?

**R:** Yes. We can have a training on our own, we can teach one another. If we hear about any of our colleague who is rude to patients we can approach them and give them constructive remarks and therefore they can abandon their bad behavior. I think this is a good collaboration.

**I:** Do you do that?

**R:** We do it. We give pieces of advice to one another among ourselves.

**I:** Do you think it is useful to ask patients if they have come to seek healthcare expecting something special?

**R:** Yes.

**I:** How is it useful?

**R:** Asking them way?

**I:** Asking them if they have come to seek healthcare expecting to gain something in particular?

**R:** Yes. It is useful because what they expect from their seeking healthcare, in any case if they come to see healthcare, it means that they want to get better. Additionally, we tell them that anyone who has come to the health facility does not return home with the same knowledge. In reality, if one comes to the health facility knowing only one thing, they will return home knowing three things. We teach them each morning before we treat them, we teach them about a given illness. So, I think that they do not return home as they came. I think it is useful.

**I:** So, the fact of asking patients if they expect to know something, how important is it in itself? What is its purpose?

**R:** When you ask them if they came expecting to get something, it makes them start thinking about what they will gain. You ask them what picture they had for healthcare providers, what they hope to get and also you ask them if they think that some of their problems will get solved or if they will return home with all their problems unsolved. You also ask them if after meeting with the healthcare provider they will return home just as they came. I think this is what I can say about it.

**I:** Do you usually do that?

**R:** I try.

**I:** When you do it, is there anything that you are driving at?

**R:** Yes.

**I:** What is it?

**R:** Well, when I ask patients, I want to know how they feel about me, what they think about our services, and also to know if they see us as people who are useful for other people or if we are useless. When I ask them, I get to know the perception they came with the perception go home with. I find out that our profession is useful, which is the reason why I keep doing it and I plan to continue to study. I also think that we should have enough training about the way we can talk to patients.

**I:** Is there something that you do to know if patients would like to know more information about their health?

**R:** Yes.

**I:** What do you do?

**R:** Well, when a patient comes to see me, I talk to them. When I talk to the patient, I have the aim of knowing what they know about healthcare. So, I understand what they knew before. There are things they know which are true and there are also falsehoods that they think are true. So, I try to get those falsehoods out of their minds and explain to them that what they learn from their friends is not the truth. For example someone can come saying that using family planning kills people because they have seen only one person who experienced side effects and immediately take it for granted that anyone else who uses family planning must experience similar side effects. So, then I explain to them that what I am telling them is not a rumor and that I know it for sure because it is written somewhere, they get convinced and then they change their perception and behavior thanks to the information that I give them.

**I:** Do you think it is necessary to explain to the patient everything they would like to know?

**R:** Yes.

**I:** Why?

**R:** Well, I think a patient is a human being and it is a person who has mind and whose aim is to live, someone who has ideas. I think a patient is not clothes that one can take to the market so that anyone comes and asks how much they cost. Instead, a patient is someone who has mind, whom we must respect and to whom we must give an answer for what they ask us and we also have to know what they need to understand things, so we must explain things to them. Because, I don’t think a patient is something for sale or an animal, a patient is a knowledgeable person who has right to know everything that is being done for them. When they want to know it, we have to explain it to them and additionally we must do anything for them while explaining it to them because it is a person who has mind.

**I:** Is it useful to let patients participate in making decisions related to the healthcare which they would like to be given?

**R:** Yes.

**I:** Why is it useful?

**R:** Well, it is useful because a patient is a human being and who has rights. So, we must respect their rights. We have our own rights but they must also enjoy their rights about everything that is being done for them. It even happens that we make an agreement with patients concerning what we are going to do for them so that we avoid any injustice to them; we make a common decision so that we avoid any conflicts between us. This helps all of us to do things without worries. Both the patient and healthcare providers reach an agreement before doing anything.

**I:** Can you give me an example of an agreement that can be established between a patient and a healthcare provider?

**R:** Yes, I will speak on my behalf. I won’t speak in general even if all of us are healthcare providers, but I will speak for the nurses. Suppose that I am going to give an injection to a patient; in that case I will make an agreement with the patient and I will explain to them “ I am going to inject you a medicine for such and such a problem, but the medicine has a,b,c side effects. Tell me if you agree that you will put up with the side effects if you experience some. I know that it has those unwanted effects although I am going to give you the medicine, so tell me if you will hang in there and don’t think that I inflicted on you the side effects on purpose.” In that case we can make an agreement. If the patient agrees, they write it somewhere. So, I think this is an example that I have given you.

**I:** When a patient would like to make a choice about healthcare, should the healthcare provider take the patient’s choice into consideration?

**R:** Yes.

**I:** When a healthcare provider shows their emotions, how does that impact the conversation that they are having with the patient?

**R:** Pardon?

**I:** When the healthcare provider shows their emotions or their feelings, how does the fact of showing those emotions play a role in the conversation that they are having with the patient?

**R:** So, concerning the emotions that a healthcare provider can have while they are conversing with a patient, for example the healthcare provider can have emotions with the intention of convincing the patient about something they are going to do for them but not necessarily because they care for the patient’s suggestion. In that case the healthcare provider thinks like, “I am the healthcare provider for you, you don’t have to right to say anything, you just have to agree with what I have done for you. You have to agree with it because you cannot treat yourself.” Those are the things that I would take like feelings.

**I:** Is it acceptable that healthcare providers exhibit their happiness or their sadness while they are with patients?

**R:** No.

**I:** Why not?

**R:** Between happiness and sadness, I think they must show happiness; I don’t think they must show their sadness, I do not support this. They should show happiness, but if a patient is sad, you should also feel sorry for them, but you should make sure you don’t let the patient see that you are sad because a healthcare provider is supposed to remain neutral and don’t lean on any side. If the healthcare provider shows sadness while the patient is happy for instance, that would not be good. So, I think the healthcare provider must remain neutral thanks to the skills that they have. The healthcare provider should not exhibit too much happiness nor too much sadness, they should remain neutral. This is what I think, from my perspective.

**I:** Why should they remain neutral?

**R:** The healthcare provider should remain neutral because among patients that they receive there are those who are sad; so if they feel very sad they won’t be able to help the patient who is sad to regain their happiness. Also, if there comes a patient who is very happy and that the healthcare provider becomes very excited, you know that anything that is too much is not good. Therefore, the healthcare provider should keep a balanced feeling thanks to the skills that they have, and therefore they can figure out how to behave accordingly.

**I:** Do you think that patients can be worried about telling their health issues the healthcare providers?

**R:** Yes.

**I:** What can be done, then?

**R:** There should be done something which is similar to a training. As I said, if I remember well, I said that healthcare providers need advanced skills in conversing with patients because if they talk to patients in an unsuitable way or if they disclose the patients’ private information, patients will be uncomfortable to talk to them in future. In my opinion, healthcare providers should be trustworthy people and who are discreet to the extent that when a patient tells their story to the healthcare provider they would feel like they are hiding it in a secret place; healthcare providers should keep patients’ stories with high confidentiality.

**I:** What experience do you get from the conversation that you have with patients whose level of education is very low, especially the illiterate patients?

**R:** What I experience happens when I think it would be better to refer the patient to a more advanced health facility but the patient refuses. In that case I try hard to explain to them but they refuse to go. Meanwhile you need something that can protect you, you need that the patient signs somewhere as a proof, but the patient tells you that they don’t know how to write. They can sign, but still their signature may not be given value because they did not write what they signed for. All those are the challenges. In addition, it is very difficult to have a conversation with an illiterate person, who is also under informed, compared to the one who is knowledgeable. It is very difficult to explain things to the illiterate because their ability to understand things is very low and it is even harder to direct them when you are conversing. It is also difficult to make them understand something.

**I:** How do you apply your communication skills to converse with patients in this category?

**R:** The way I apply the skills when I start to converse with them, it’s just what I told you before: conversing with a patient beforehand clears the way for you to know well the patient and vice versa. When we are conversing, I ask them their demographic information and other pieces of information. So, by the time we make final decisions, I don’t need to ask them if they studied or not because I have known it early before. So, to apply my skills, I would say that I try to masquerade as the patient so that we are able to understand each other. There are words, or terms that I cannot use, for example I make sure I don’t mistakenly say something in French. I try and speak a language that they understand and at the same time use easy to understand words so that we understand each other.

**I:** Do you think that the Rwandan culture plays a role in the conversation that a patient has with the healthcare provider?

**R:** Yes.

**I:** How?

**R:** I would say that Rwandans have a common language. Even long ago, Rwandans used to converse even if those who had studied were very few. They memorized all kinds of information like poems and so forth. They had a strong ability to memorize things. Therefore the Rwandan culture plays a big role in the conversation that a healthcare provider has with the patient because even if the illiterate patients have low level of knowledge, they still know what someone should not say and what someone should say because there exist taboos in the Rwandan culture.

**I:** Now try to relate those things to the conversation that you have with patients. The fact that you can say something and not say some other things, relate this to the conversation you have in consultation room?

**R:** Well, let me relate it. Sometimes an old patient comes with a certain illness and then they can be afraid of telling me directly what their illness is. For instance if the illness has affected their sexual organ, the patient can just say “*I have a problem in the southern part.”* Therefore, if I haven’t a clue about metaphoric language used by old people, I can just assume that the patient is talking about southern province whereas they are referring to their sexual organ. Also, if a patient has diarrhea, they can come and tell you that they are “*passing by the under part*” You can think that this expression means to throw things down whereas it simply means that they have diarrhea. So, all those things show that Rwandans have their particular way of expressing sort of secret things and say them in other words. In case you don’t know the metaphoric language of adult people or metaphoric expressions of the language, you can treat the patients in a wrong way. If they say *“to pass by under part”* you can think that it is something else. All these things can prevent you from treating the patient in a proper way and as a result you can treat them for an illness which they really don’t have.

**I:** So, if I understood what you said, did you want to mean that people who are old, they beat about the bush when they are explaining things?

**R:** Yes.

**I:** Instead of telling you directly…[interrupted]

**R:** They tell you using a metaphoric language. They do not say things in a direct way. There may be other parts of the body which they refer to using other words and therefore you may get stuck and fail to figure out what they want to say.

**I:** Yeah, in your opinion, what are the factors that usually affect negatively the conversation that you have with patients? But this time, I am focusing on the side of the patient. The factors that are coming from the patient.

**R:** I would say that it is because of acquaintance. It happens that a patient comes and finds that they know you or you know each other or you are neighbors. So, when they come with a certain illness while you are neighbors or acquainted to each other, they may think that you will tell their illness to other people in the neighborhood or that you will remember about their illness every time that you see them and as a result they decide to not tell you about their illness.

**I:** Are there other reasons that you think can spoil the conversation between the healthcare provider and the patient? Still, factors arising from the patient’s side.

**R:** Yes. There are other factors like the fact that a patient can take you like somebody who wronged them. A patient can come in the consultation room and upon seeing me, they take me for somebody who killed their relatives during the genocide. For example a patient can come and say, “That woman, isn’t she the one who was at the roadblock! She looks like the one!” So, when a patient has taken me for the murderer of their family member, this can be a challenge and they cannot trust me while in reality I am not the murderer; maybe we resemble but the patient may think that I am the one who did it. A patient can come and take me for their mother whereas their mother is no longer alive and then the patient can come and say many things, and it can also be a challenge. It can happen in a positive way when the patient says for example ‘That one looks like my mother” and therefore they need that I give them the maternal affection that their mother would be giving them and as a result, they can go off the subject and tell me what they should not tell me; I think this can also happen.

**I:** Does it happen sometimes?

**R:** Yes, it does happen.

**I:** In your opinion, what are the factors that usually hamper the conversation on the side of the healthcare provider?

**R:** On the side of the healthcare provider, the factors that usually affect negatively the conversation are lack of skills in conversing with patients and also awkward behavior. For example a patient can come and speak angrily to the healthcare provider and that the healthcare provider also becomes angry immediately fail to talk friendly to the patient. I can refer to all those factors as lack of skills. In addition, the healthcare provider may be someone whose mood never changes, someone who never laugh, who is always angry and who doesn’t know how to care for patients. I think that’s it.

**I:** Are there other factors that usually spoil the conversation between the healthcare provider and the patient? And the factors being generated by the work conditions at the healthcentre?

**R:** Yes.

**I:** What are they?

**R:** Well, concerning the work conditions at the health centre, there is an involvement of the head of the health centre. Sometimes the titulaire can always harass an employee to the extent that the employee is always melancholic, always sad and seems to have gone traumatized and therefore they discharge to anyone who comes the anger that is inflicted on them by their employer. The employee becomes like a person who has trauma and therefore they discharge their trauma to patients. Also, there is another factor of lack of equipment. An employee can be in need of pieces of equipment that can help them to treat patients but they do not have the equipment; and all those are the factors that can make the healthcare provider to not give a good care to people, that is, when they need equipment but they don’t have them.

**I:** Okay. Give us examples of things which are difficult to tell patients about?

**R:** I think you have already talked about difficult things. You touched upon the fact that the Rwandan culture is also among difficult things because it is very difficult to discover the meaning of the metaphoric expressions used especially by old people. In addition, it is difficult to talk with illiterate people because they have low level of knowledge. Those people have a negative perception of things and it is therefore uneasy to guide them during the conversation.

**I:** So, if I understood you well, you wanted to show that the nature of patients or the communication styles of patients can be a challenge when you are conversing with them. But now I would like to put an emphasis on particular things which are hard to tell patients about.

**R:** Well, I would relate those things to counselling. It is hard to tell a patient about their illness when it is a serious one or if it is chronic. Let’s take another example: you know that in the consultation we take blood samples and we do tests for HIV/AIDS and we do that test even when patients did not plan to have it, I mean PIT. They just come to seek healthcare for a certain illness, like chronic ones or sexually transmitted diseases. Yourself you make a decision and say “If I take a patient’s blood sample and do a test for them and take time for counseling them when I have very short time and that there are so many patients, it will be very hard for me to explain to them their results because I have very short time.

**I:** What do you do in those cases?

**R:** You do it but sometimes you encounter with a number of challenges. Suppose that I have one hundred patients to see including one with whom I will spend one hour explaining to them their results. In order to explain thoroughly their result while other patients are knocking on the door, I will be forced to make the patient understand their result so quickly that I will be able to receive other patients. So, it will be very difficult to deal with that situation and when I tell them their result they will not understand. On top of that, there is no colleague of mine who was trained about counseling so that I would refer the patient to them for immediate care. Therefore I will refer the patient to another health facility where there are nurses who were trained in counselling, but if there was a colleague of mine who was also trained, we would immediately switch jobs. I think you have understood my challenges.

**I:** Okay. When you were doing your job, did you ever receive a patient whom it was difficult to talk to because of a given problem that they had?

**R:** Yes.

**I:** What was their problem?

**R:** Well, I think the problem was just the one that I was explaining to you. It was a young girl who reported that not even once did she have sexual intercourse. Yet, she came to seek healthcare for sexually transmitted diseases at multiple times and one day I decided to take her blood sample and did a test without letting her know because she had denied having had any sexual intercourse. So, when the result was available, I found that she was HIV positive. Because she had denied having had sexual intercourse, I hadn’t told her that I had done a test for HIV; so, I struggled to find ways to explain to her that she was HIV positive. It was very difficult for me to tell her about her result. It was so difficult for me that I didn’t know where to start. I was afraid of telling it to her lest that she would go in coma. Before telling her the result, I first of all went outside and checked with my colleague whom I believed had more skills that I had. I asked them how I would proceed. It took me a long while and I seemed to have become ill or to have had trauma. I think I received that case.

**I:** What happened in the end?

**R:** I ended up explaining it to her and then she sort of had depression. I conversed with her for so long that it turned up necessary that I called a colleague to come and stand in for me and then I went somewhere else with the girl in order to tell her about her results in details. It took me the whole day. I didn’t go for lunch, I spent the day together with the patient because she laid down and fell asleep because I tried to explain to her but she didn’t understand. I tried to give her medications that we usually use, *diaspa*, and she later woke up and we resumed our conversation and she accepted her result but it took me the whole day. I spent the day with her, I didn’t have my lunch, it wasn’t easy.

**I:** Did you receive a patient whom it was difficult to talk to because they had a mental problem?

**R:** Yes.

**I:** How did you handle the situation?

**R:** I received a case of a patient who had a mental problem, I was still a young girl at the time. It was a battle for me to get out of the consultation room. In the end, what was healthcare changed into love. I struggled to get out of the room; in short he sort of saw that I would become his fiancé whereas I was treating him. He asked me all details about me, I gave him my personal information, my phone number, my name and everything. I was doing my internship and I was still studying. By stroke of luck, someone else came and replaced me and I went out and they continued to treat the patient.

**I:** During your work, did you ever receive a patient whom it was difficult to talk to because of their disability like deafness, dumbness or blindness?

**R:** Yes.

**I:** How did you cope with this situation?

**R:** Well, as we said, I think they should give us training on communication skills so we would be able to talk to the deaf and the blind. I once received a deaf-and-dumb patient and to communicate, the patient used signs. To be honest, after the patient had left, I started to ask myself whether or not I had treated the illness which he had told me. I gave him medications but after he had left, I asked myself if he had understood the instructions I had given to him. Because I used signs that I didn’t really know, and I didn’t also know what he meant by the signs he used. Sometimes you hope that even if you use signs the other person understand, but in reality I gave him the mediations but I remained worried. And as I expected, he returned after a few days. And when he returned, he said the same thing, but this time I sought help from my colleague; I usually seek help from my colleagues. I told my colleague, “Did you ever treat so-and-so?” because that patient was used to coming to seek out healthcare. “Maybe you can come and help me and then see if what he said is what you know. Do you understand sign language?” I asked my colleague. That is something that was so difficult for me. As he returned only after one week, I thought that the medications I had given him were not for the illness which he had told me, and I told myself that I hadn’t understood what he told me.

**I:** During your work, did you receive a patient whom it was difficult to talk to because they had a difficult personality?

**R:** Yes.

**I:** What did you do? How was that patient?

**R:** It was a quarrelsome patient. It was soon after I had started my job. I had given him an IV (drip) and suddenly the patient complained saying that I had injected them in a bone. It was not my first time to give someone else an IV, even if I had started the job shortly. I was a new employee somewhere at a health centre. I didn’t know that it was the patient’s culture to falsely accuse new nurses of some mistakes. He used to be hard on new employees by accusing them of falsehoods and that they did not know how to administer medications to patients. I first thought that I was the only one who was accused of mistakes but I later of discovered that there were many other nurses who were also falsely accused of mistakes. But I was very terrified at the time and I had difficulty explaining to him that I hadn’t injected the IV in the bone because the IV was dripping. I sweated because of fear and I went far to convince myself that I had probably injected the patient in the bone but I asked myself how come that the IV was flowing in the tube.

**I:** Is it necessary to tell a patient about an illness or a problem which you think they have?

**R:** Yes.

**I:** Why is it necessary?

**R:** Well, it is necessary to tell them about it because if you don’t tell them, no-one else would tell them. In that case they would return home without knowing what they are suffering from, and they wouldn’t even know how to behave. They would not know whether or not they have an illness which has such and such complications or if there are things that they have to give up because of the illness or if there are things that they must stop doing or stop eating. In that case if you do not explain to them their illness, they cannot know how to change their behavior in order to be able to live with their illness.

**I:** What can you tell a patient when you are not able to identify their problem?

**R:** Well, when I haven’t been able to identify the patient’s problem, I admit it and tell them that I did not know their problem and then I tell them that they are free to go anywhere else they have more skills than I do. ‘I wasn’t able to know your illness. But I can give you a piece of paper that you can take somewhere else there is more knowledge and where they can help you better.” That is when we give transfers to patients.

**I:** Concerning medications that a healthcare provider prescribes, is it necessary that the healthcare provider explains to the patient the medications that they have prescribed for them, how the medication works, how the patient must use tit and any possible side effects that it may have?

**R:** Yes.

**I:** Why is it important?

**R:** It is important because when you tell them what type of medication it is, how to use it, I think it is important because you can give them a medication when they once had it before and experienced unwanted effects; if that is the case they can tell you “I usually experience side effects when I take this type of medication. It had a,b,c problems.” Then, you can change it instead of giving it to them and make them experience the side effects. In addition, when you explain the medication to the patient and any possible side effects, the patient tries to hang in there when they experience the side effects.

**I:** Some of patients in Rwanda think that they are not given enough information about the medications. Is that true, based on your experience?

**R:** Yes.

**I:** Why does that happen, in your opinion?

**R:** It is because of shortage of healthcare providers whereas patients are so many. In addition, I would also say that healthcare providers have little skills because they didn’t study up to a higher level. They are still treating people while they finished studying long time ago.

**I:** Do some of your work conditions or situations at work here at the health centre negatively affect the conversation that you have with patients?

**R:** Pardon?

**I:** Do some work conditions or do some situations at work hamper the best conversation that happens between the patients and you?

**R:** Yes.

**I:** How is it?

**R:** While I am examining a patient in the consultation room a colleague can suddenly come and ask me something and while I am answering to the colleague’s question I can forget where the patient and I stopped our conversation; so I may need to ask the patient to remind me: “What did you tell me concerning the signs of your illness?” Actually, the patient can also forget where we stopped the conversation, and therefore both the patient I don’t know where we stopped. Therefore the patient can have a bad image of me, because of my colleague who suddenly entered in the consultation room. My colleague did not respect the place where I am working because they feel that as healthcare provider they have right to enter anywhere at anytime. That is something that I can say that it is a problem.

**I:** What do you do if a patient requests for a transfer to go to the hospital when you think it is not really necessary?

**R:** There I think that the patient’s right must be taken into consideration as long as it does not cause problems for me. When a patient needs a transfer to go to the hospital when it is not necessary, I give it to them but I mention on it that it is they suggestion; so they go wherever they want to seek healthcare.

**I:** Do you have any problems when you are conversing with patients about health problems in Kinyarwanda language?

**R:** Yes.

**I:** What problems?

**R:** Well, I cannot say that Kinyarwanda language is poor because it is our own language, and that I am a Rwandese. However, there are some terms that we learn but you don’t know how to explain them in Kinyarwanda, they don’t have equivalents in Kinyarwanda. When you have to explain them to the patient, you try to paraphrase, and you may even tell the patient wrong explanations because you are just trying to help them understand, you try to explain them but there are no equivalents in Kinyarwanda.

**I:** The fact that you were taught in French or in English as you have just said, is it a challenge that hinders your conversation with patients in a proper way in Kinyarwanda?

**R:** Well, it is not a very big problem, but usually when we are learning, we use those foreign languages, we do not study in Kinyarwanda. We do not have time to discuss what we learn in Kinyarwanda, we do not take time to try and practice what we learn in Kinyarwanda, we do not do something like “debate” in Kinyarwanda. We do not do things like “sketch” in Kinyarwanda. In fact we study using a language which is not Kinyarwanda but everything that we do everything for our patients and talk to them in Kinyarwanda and we have difficulty saying what we learnt in Kinyarwanda. For example if we are educating patients about sexually transmitted diseases, they can ask you a question and you don’t know how to answer it in Kinyarwanda. Also, if you recommended a medical test for a given illness, a patient can ask you what type of tests you have requested for them and you don’t know how to say it in Kinyarwanda. Suppose that you found “white blood cells” and it is a patient who is under informed; will you tell them that the test is “whitish blood”? I don’t know, you find that it is a challenge when it comes to explaining to patients their illnesses.

**I:** So, what do you do when you have to explain medical terms which are in English or in French but which don’t have equivalents in Kinyarwanda? How do you go about it?

**R:** That is a challenge for me and sometimes you find yourself having spoken in language that patients do not understand. I may run short of equivalents in Kinyarwanda so I can use them to explain something and then I tell patients that I am going to say the term as it is and add that it doesn’t have equivalent in Kinyarwanda. In that case some understand and others don’t understand.

**I:** Now coming back to the consultation room by the time you are conversing with patients; what do you do at that time?

**R:** You now, I have told you that I try to paraphrase and help the patient to understand it in Kinyarwanda; but I still think that I should be saying it in another way and I am not sure that I have paraphrased it correctly; I feel afraid that I may have said something wrong. Sometimes you sit down, for example when I am going to explain to them their test results. The patient asks me ‘What is my test result?” I have given you the example of “white blood cells” and then I ask myself “Shall I tell him/her that it means “small white balls” or that it means “yeast” then I challenge myself “will I say that it is yeast” when it is not for making bread? It is just a serious issue. You lack ways to explain it and it disturbs you in your work. Sometimes you close the door and go to ask your colleague. As you go out you tell the patient ‘Hold on, I am coming back in a minute.” And you go and ask your colleague “How do you say this in Kinyarwanda?” So you understand that you are forced to leave the patient alone in the consultation room while you shouldn’t leave them, but you do so because you have no choice.

**I:** Are there other problems that we haven’t discussed that you encounter with when you are conversing with patients?

**R:** Well, I think we have discussed most of them.

**I:** What can be done to improve your skills in conversing with patients?

**R:** So, in the beginning I talked about what can be done. It would be helpful if there was a school where they teach people how to engage patients in conversation. They would come and give us trainings or those of us who have means and enough time they can go and study about it so that they are able to receive and talk to patients in an appropriate way.

**I:** So, patients are different and of course they express themselves differently. How do you apply your communication skills to adapt yourself to different communication styles of patients?

**R:** Pardon?

**I:** I said that patients are different and that they understandably talk in different ways. How do you apply your communication skills to cope with different communication styles of the patients?

**R:** Thanks to my skills, I know that a patient comes alone, patients do not resemble, and each patient is unique. So, if I know that each patient is unique, I will be able to use my communication skills to talk to them. Because if I am done with one patient and that they go out, the other one who will come next will appear new to me. Thanks to my skills, I am able to receive each one in a particular way and treat them as new and consider that what they are telling me is new to me. It helps me to receive them because each one is different from the other and they are not the same.

**I:** In which ways can a healthcare provider help patients to talk to them more comfortably during consultation?

**R:** During consultation, I think the healthcare provider should give enough time to patients and he should feel comfortable to talk to the patients and vice versa. In short, patients should see him as a healthcare provider and not like a businessperson, or foreign exchange agent; the healthcare provider should manage to make patients see them as someone who is sympathetic for them, I mean a honest person.

**I:** What do you do when a patient cries?

**R:** When a patient cries I let them cry. What I can help them is like to give them something similar to the one you have brought, I mean tissue paper so that they sweep themselves. When a patient cries, I firmly close the door lest that no-one comes in suddenly. After they have cried, I see if they can be able to resume the conversation with me, but I first of all let them cry. I let them cry because I think crying is also another way to get relief or a way of showing what makes someone sad.

**I:** Is it important for you to help patients control their emotions which result from an illness that they have?

**R:** Yes.

**I:** What is the importance of that?

**R:** It is important for me to know the feelings of patients as we converse because it is thanks to their feelings that I get to know who they are. If the patient is angry, I will be able to know that they are angry and therefore I will know that I have to remain kind towards them and avoid upsetting them. If the patient is just saying little, I know how to behave accordingly. Sometimes you ask someone something but they do not answer you, in that case you let them but you still continue to talk to them. One day it was during the commemoration week when I received a patient who didn’t talk whereas they were able to talk. Therefore I gave them a piece of paper to write down their problem. The patient wrote the problems that they had and I also wrote down the answer and then I returned them the paper. In the end, the patient talked. So, I told you that I studied about emotions, I have a lot of things that I can tell you.

**I:** Thank you for that. What you are telling me is it the way you manage the patient’s emotions. Now, is it important to help the patient manage their emotions on their own?

**R:** Yes, it is.

**I:** How important is it?

**R:** Well, with regard to helping patient to manage their emotions, I would say that we really have very short time, but if we use it properly we can have good outcomes. If patient’s feelings are making them keep silent, or be disrespectful, or be quarrelsome or speak rudely to people, or have a weird look at people, there is a way that I can talk to them and bring them back to their normal mood . I can also tell them about it in a non-violent way. I can tell them for example, “When one comes into the consultation room, it is always good to tell healthcare providers about one’s pains otherwise they cannot know what problem one has. It would be better if you would answer when someone else asks you something.” There are kind words that one can use without upsetting someone else and which can make the patient change their behavior the next time they come to the health centre.

**I:** How does that help the patient?

**R:** You know, you help someone else to help themselves. If they had a certain behavior, they change it.

**I:** Is there something that you usually do to make sure patients have understood what you are saying?

**R:** Yes.

**I:** What do you do?

**R:** I ask them some questions in between the conversation.

**I:** Why do you do that?

**R:** I do it so that I know if we are together. It may happen that a patient tells me about their illness but they forget other things in a short while or when we are conversing, the patient may forget what we said in the beginning. So, I ask them question that kind of awaken them in order to see if they are in the world where I am.

**I:** Should a healthcare provider let patients play a role in the healthcare that they would like to receive?

**R:** Pardon?

**I:** Should a healthcare provider help patients to participate in the healthcare that they are given? How can he do that?

**R:** The healthcare provider can treat a patient and ask them ‘Are you satisfied with the intervention I have done for you? Do you think there is something else I should have done for you? Are we together? Am I bothering you or I am making you happy? If you think I am doing for you something that you do not want, you are free to tell me? Are you satisfied with what I am doing for you?” I think it is in that way that I do it.

**I:** Why should a healthcare provider do that?

**R:** I told you that a patient is a human being like us, they have their mind, they have rights and ideas like we do. So, the healthcare provider should do that in order to see if the patient – the patient is not a domestic animal or a plot of land or items of clothes; a patient is rather a person who has mind. Therefore the healthcare provider should do that to make sure they are both together and if patients are pleased with what healthcare providers are doing for them.

**I:** We have been talking for a while. Is there anything else that you would like to add on what we have discussed?

**R:** The other thing that I can add is that thanks to the conversation you have been able to understand what is going well and what is not going well. I think that you should make a positive or negative critical analysis of what we have discussed. Another thing I can say is that we would like to be informed about the findings that you will get from this research that you are doing, you should share with us what you have found. If you share with us the outcomes, we will be able to get the skills that we were talking about, the skills concerning how to better receive and care for our patients; it will be very helpful for us.

**I:** Thank you so much!

**R:** Thank you too!

**I:** And then, do you think there are other questions that we should have asked in this research? Any question that would contribute to the improvement of the conversation between a healthcare provider and a patient?

**R:** Aha, yes.

**I:** Which ones for example?

**R:** Well, of all the questions that you asked me, I don’t think you have asked me “How should a good conversation with a patient last for?”

**I:** Okay, any other question?

**R:** Yes, there is another one. “What a good conversation between a healthcare provider and a patient is like?” um, you have asked me this one! There is no other question.

**I:** Yeah, that was the last question that we had.

**R:** Thank you!

**I:** Thank you so much! [*Both interviewer and respondent have a big laugh as they finish the interview*]
