# Supplementary material for: Providers’ perceptions of communication with patients in primary healthcare in Rwanda
Source: PLoS One. 2018 Apr 4;13(4):e0195269. doi: 10.1371/journal.pone.0195269 (PMC5884556; doi:10.1371/journal.pone.0195269)
Supplement: S1 Dataset — (ZIP) [file pone.0195269.s001.zip › S1 Dataset/PPC-Provider 9.docx]

**PPC-Provider 9**

I: Interviewer; R: Respondent

**I:** We are about to start our conversation. May I start by asking, what you can tell us about the conversation the patient has with nurse in the consultation room at the health center?

**R:** What I can tell about the conversation the nurse have with the patient at the health center depend on the stage of the patient. The stage I mean young, old, and also the kind of the sickness the patient has. The example I can give here is that when an old man or women comes is very open, he/she tells everything even the ones you are not in need of, but when a young girl comes she may not feel comfortable of talking to you depending on the sickness she has may hide something, but as you keep talking she becomes comfortable and start being open and tells you everything.

**I:** Uuh, thanks. What is the importance of the conversation between patient and nurse has on the work you do in the consultation room at the health center?

**R:** It is important because every patient has unique conversation. I don’t have the same conversation with every patient. It comes important to some patient to the point that he/she may come back and tells that you helped and gave good advices. We see that the conversation we have help us and also help the patients I don’t know how I can say it. There is a way the patient comes to you and try to have unity and make her/him feel that you are together and start telling you everything. When he/she tells you everything, it helps on the side of nurse because it allows you to know the patient history, and allows you to have the conversation related to the patient sickness.

**I:** Uuh. Can you tell us in detail how is the good conversation between patient and nurse?

**R:** The good conversation is that here at the health center are many patients, and you don’t get enough time to speak to the patient, But when the patient introduce himself to you and you also introduce yourself to him/her there is a time you have a conversation knowing each other and the patient tells you everything without hiding anything from you. But there is a time he/she enters and starts being rude to him/her due to our different personalities, he/she will also not tell you anything. But when he/she enter and receive him/her well and sees that there is no problem, you received him/her with care, in this case you have a good conversation without hiding anything from you.

**I:** Uuh. Is it important for the nurses to have enough knowledge related to having conversation with patients?

**R:** Uuh. It is very important because there are patients with complicated cases. For example patients with mental health illness. When patient has psychological problem it requires to go deep and also use other strategies. So it is important for the nurses to have enough knowledge.

**I:** Uuh, why do you think need to have a good conversation with the patients who comes to you?

**R:** The nurse needs to have a good conversation with the patients who come to him/her so that he/she can get all information he/she needs. When you don’t have a good conversation he/she doesn’t tell you all information, you cannot also give him/her all needed information. I think it is important to have a good conversation with the patient.

**I:** Do you think the good conversation you have with patient can help in improving the healthcare or improving the way you look after the patients?

**R:** Yes. **I:** Uuh, how? It can help because when you had a good conversation with the patient even when you don’t give him/her the medication can go back feeling well, and when you have a good conversation it helps to give good treatment, even I you can give the medication the best treatment is the way the patient think. I may give you good medication but because you think that the medication will not help, and take it but don’t help. But if we start by having a conversation, and goes without medication, you may go saying that the nurse told me this and this I know that I will get better, and you find out that he/she gets well depending on the sickness he/she had.

**I:** Uuh. Do you think having enough knowledge about having the conversation with the patients, can help you more in helping the patients who come to you?

**R:** Yes**. I:** Uuh, how? On my side, having enough knowledge can help because there are some cases you may receive and find out that you need support, isn’t it? Let me say that I receive the patient with mental health problem, and find out that it is challenging to have a conversation with him/her, I immediately think that there is someone who is trained and call him/her for support. It means that having enough knowledge helps much!

**I:** Have you had lessons or training which helps you improving the way you have conversation with patients who come to you?

**R:** I didn’t have unique training, but where we start I had a course called health education that’s the one which helps and also use it in having conversation with the patient.

U: Uuh. Do you think is important?

**R:** It is very important. **I:** Why do you think is important? Having a conversation with the patient? **I:** No, being trained. Generally it means how to talk to the patient or steps to be followed, isn’t it? **I:** Uuh. Trainings are important because there is a time you receive different type of patients and training is needed on special cases. If there is a new disease, people should be trained on it and be able to have a conversation with the patients.

**I:** Uuh. On the measure of 1 out of 10, how many marks can you give yourself on the knowledge you have about having conversation with the patients in the right way?

**R:** I can give myself seven. **I:** Uuh it means that you can’t give yourself 10/10 there is three missing, is there a gap? Uuh. **I:** Where do you have a gap related to the knowledge you have related to not having a good conversation with the patients who come to you? It doesn’t mean that I am not able to talk to them, rather we have the conversation in a way “ tut”, you find out that even yourself you are not satisfied and start thinking that if you were trained about it it should be done like this. **I:** Uuh. I can give an example of mental health patients **I:** Uuh. That’s where is more complicated to me. I do try my best on other cases but when it comes on mental health patients, having conversation with that patient is challenging.

**I:** Uuh. What do you think need to be improved?

**R:** Improved? **I:** Uuh. What do I need to be improved? **I:** What do you need to be improved in order to have a good conversation with the patients? Having enough knowledge related to all cases, nothing else.

**I:** Ok, How does the relationship you have with other nurses makes the conversation you have with patients go well?

**R:** Working with your together with your colleagues is good. **I:** Uuh. By giving an example, you may receive the patient you know each other. You may be going to receive the patient you know each other and don’t feel comfortable of sharing case with anyone, in that case you negotiate with your colleague and one of them have to help rather than you who know the patient. Because he/she may have complex thinking you know each other, and you also know all his/her problems. Another thing is when you have certain weakness or counselling someone, the case we mostly meet is unwanted pregnancy where the child comes pregnant and doesn’t want that pregnancy may refuse to accept that she pregnant and convince you how she didn’t have sexual intercourse. In that case when you have a conversation with her and find out that she doesn’t become convinced or accept the situation, you have to use your colleague and talk to her and see if you can achieve your goals.

**I:** Do you think it is important to ask patients if there is something specific they are expecting which make them come seeking for treatment?

**R:** Expecting? **I:** Especially because they come seeking for treatment? What they are expecting how? I am asking if it is necessary to ask patients if there is something specific they are expecting by coming to seek for treatment? Yes **I:** Why? It is important because it helps to know their thoughts on it, and also knowing how they can behave in case they had consultation and receive a particular result. When he/she tells you that, it enables you to have a conversation and brings him/her in the right path where he/she is not thinking well.

**I:** Uuh, Is there something you do in order to know if the patient who come to you wants to know more information about his/her health status?

**R:** What I do? **I:** order to know if the patient who come to you wants to know more information about his/her health status? Yes. **I:** What do you do? It means that there is a time you ask the patient to do medical test, and before you tell him/her the results start asking nurse what am I suffering from? **I:** Uuh. You may give it to him/her and after ask is there has another question? If he/she wants you to know more he/she tells you or give him/her answer or send him/her to take other exams even when is not necessary to do so depending on how you had the conversation **I:** Why do you do that? It helps me a lot because it enables to help the patient well.

**I:** Uuh, ok. Do you think it is important to explain to the patient everything he/she needs to know?

**R:** It is important **I:** Why do you think is important? It is important because the patient need all information related to him/her by following the conversation you have together, and finding the right way to do it because there is a time it may be difficult to receive certain information but based on the conversation you had you can make her/him understand how to accept it by giving explanation. Where you find it challenging or requires more knowledge you need to refer that’s an example. There is a time the patient comes telling you that he/she is sick in this way, I have a liver that’s an example. Myself I don’t know about it because here we don’t test it let me give you a transfer to the hospital and do test the doctor will know if you have it or not, in that case you need to help each other so that the patient can know the information needed to know about his/her health status.

**I:** Uuh. Is it important to make patients who come to you, participate in decision making related to the way they wish to treated?

**R:** It is important. **I:** Why? It is important because even though you had educated him/her and told him/her the problem and what can be helpful, he/she also tells you. For example there is someone who may refuse to tell you something and another one tells you that even though the child is vomiting like this I can’t be hospitalised because I have other 3 children at home and they have no one to take care of them isn’t it? **I:** Uuh. You try to educate him/her by telling her if he/she returns home without taking medications. For example in case he/she has malaria, he/she needs first to get arthesunate. You need to educate him/her in order to understand and stay become hospitalise by knowing the reason and also feel that it is necessary. What helps is the conversation and the patient become convinced about a particular illness, and what should be done for him/her. But he/she can’t come obliging you to treat a certain illness, rather you are the one who consult and see what is needed by explaining to him/her.

**I:** Uuh. When the patient has to make choices about the treatment, should the nurse give value to that?

**R:** I value it because there is someone who may have a chronic disease, and someone with chronic disease there is a particular way he/she is helped. For example there is someone who may come and say that he/she has epilepsy, and I take this medications. And I ask if the medication is helping and say yes, is there any side effects? And say no, go and continue the medications. There is someone who come with choices and have a good logic. There is also someone who comes with choices but without good logic **I:** Uuh. You have to see if his/her choices are ok, **I:** Uuh. If there ok you can accept it but they are not ok you need to have a conversation in order to have the same understanding.

**I:** When the nurse is showing his/her emotions, how does the emotions contribute to the conversation he/she has with patient?

**R:** By showing emotions? **I:** Uuh. I don’t know how I can explain **I:** In order to be clear, the emotions means happiness, sadness and other. When the nurse is showing his/her emotions, how does the emotions contribute to the conversation he/she has with patient? First of all is that the patient sees that you are listening to him/her, when someone comes suffering you have to show that you are listening to him/her, when he/she sees that you are together you have a good conversation but he/she can’t come crying and also cry! **I:** Uuh. Or by cerebrating and you also cerebrate. You need to find a way to manage it.

**I:** Is it acceptable for the nurses to show happiness or sadness while they are with patient?

**I:** According to me it is not good **I:** Uuh, why is it not good? As for me the way I understand it, and see is that the person may come suffering too much but you don’t need to show it much. If you become sad how you will be feeling in the evening since you receive many patients! **I:** Uuh. You may also get tired, you have to be calm no matter how the patient may be suffering he/she can’t come crying and also cry! Yes you need to react by showing that it is challenging or helping fast but you don’t have to feel sad and show him/her that! You can’t even manage that.

**I:** Do you think patients can have challenges about talking to nurses about problems related to their health status?

**R:** Yes **I:** What can be done? First of all what makes the patient have challenges is the way he/she sees the nurse. It depends on the way the nurse receive the patient, that’s the first thing which stress the patients and prevent them from being open in the conversation you have. What should be done is that the nurse should have empathy, by putting himself/herself in the patient position. If you wake up not feeling well, you don’t have to react toward the patient rather come feeling that you are going to receive people who are suffering and need help. First of all nurses need to remember deontological ethics.

**I:** How is it having conversation with the patient with low educational background? For example like patients who doesn’t know how to read and write?

**R:** There is a time you receive them with small understanding, and requires much energy to educate them. There is also another one with inferiority complex and immediately tells you that, and make him/her feel that there is no problem, that’s the situation what I mostly meet. Someone may come telling you that he/she doesn’t know how to read and write, you also have to make him/her feel that it exist and inspite of that life continue. There is also another one who lives like there is no problem, and also without knowing that. **I:** Uuh. It sometimes helps you also, there is a time you have a conversation with someone and find it difficult to be on his/her level but you see it and try your best.

**I:** How do you relate the knowledge you have in having conversation with people or patients in that stage?

**R:** They are not challenging to me. They are not challenging because some of them are grown up people. Grown up people want to tell you many things, and make you enter in the conversation fast because they are not challenging. An old man and old woman comes wanting to tell you everything. To me it is not difficult having conversation with them.

**I:** Does Rwandan culture influence the conversation the patient have with nurse?

**R:** Yes **I:** How? Mostly I can talk about women **I:** Uuh. Let’s talk here at the health center, the nurse work in all services. When you are going to facilitate labour with long nails, putted on make up everywhere the patient may see you as you are not on the same level. **I:** Uuh. And tells you small information. You have to try to be humble by wearing comfortable clothes so that when the patient sees you may not see you behaviour as unusual. **I:** Uuh. The culture contribute much. **I:** Uuh. The patient can tell you that a certain nurse is like this and this, when he/she sees that, start feeling that he/she can’t tell you anything and stops the conversation. Rwandan culture helps a lot.

**I:** Uuh. Based on how you see, what that make the conversation not go well between the patient and the nurse due to the patient reasons?

**R:** It depends on the way he/she lives at home, where he/she lives may make him/her hide the sickness. The condition he/she lives in is mostly an obstacle to the conversation the patient has with his/her nurse. Giving an example there is a time you receive a child who is pregnant and lives with her aunt, telling you the time she received her last menstrual period is a problem, and tells you that she received it yesterday while she has advanced pregnancy. It means that the way she is living her life makes the conversation not go well. This is the issue that can come from the patient.

**I:** Uuh, are there other things which make the conversation not go well between the patient and the nurse because of the patient?

**R:** Another stage is when he/she can come without being able to talk but in this case you use his/her colleague, or someone else who is taking care of him/her by giving you all information needed or by telling them the necessary information.

**I:** Uuh. By going to the nurse’s side, what are the reasons that mostly cause the conversation not go well between him/her and the patient?

**R:** The nurses cannot say that they don’t have something to talk to the patient, or you were not able to talk to him/her. Except you who feel that there should be something important missing which to be added on. Another reason is where the patient is not comfortable of talking to you, that’s an example and it doesn’t mostly happen. What I said that the patient may come without telling you something, is where the conversation may not go well. Another thing which may make the conversation not go well is the time, the time may become small and make you say things in small. For example if you are in VCT service, he/she may come and do medical examination and you know that he/she should have pre and post- counselling or if he/she brings the results and find out that you have many patients at 5 pm that’s an example, and the results are negative that where you take small minutes because you have many patients, and the nurses are few. The way I understand, time can make the conversation not go well the way it should be.

**I:** Uuh. By coming back to the consultation process, are there other reasons mostly seen related to the nurse that may cause the conversation between the people received not go well?

**R:** For me I don’t see another reason apart from small time, or there may be something small you didn’t do because you was not trained on it. I don’t see another reason.

**I:** Based on the health center functioning, what are the reasons that mostly make the conversation not go well between the patient and the nurse?

**R:** Like a health center? **I:** Uuh. We mostly have many patients and find out that one nurse has received around sixty patients and get tired. You reach in the evening feeling tired, and there are many patients that’s what make the conversation not go well.

**I:** Can you give an example of things which are difficult to talk to the patients who come to you?

**R:** Difficult: **I:** Uuh. What is difficult is explaining the sickness to the patient he/she was not expecting for. That is what is difficult because it requires much energy, and strategies. Explaining the sickness he/she was not expecting for and difficult to accept, this is challenging to explain. **I:** Uuh, how do you behave when is like that? When is like that you try to reserve minutes by giving him/her enough time by taking enough time with him/her. When you have enough time with him/her, no problem is complicated.

**I:** By the time you are working, have you ever met a patient who was difficult to talk to because of a particular problem he/she had?

**R:** It happened. **I:** Uuh, what was the problem? The problem which happened I received a woman, and the man they were living together. She had a 2 months baby, and they had 3 children at home whose birth spacing were short. By doing consultation it was just normal problem while it was pregnancy. Ask her to do medical examination by arguing telling me that she is not pregnant and told her to try and do it and found out that the test was positif. I started by counselling her before I announce the result and told me that I don’t have to tell her that she is positif but kept teaching her and after getting the result cried and told me that if there is medication to do abortion I can give it to her. Teaching her that time took me much energy, and told her to call the husband so that I can teach her while they are together. That’s how it ends. That’s one of the case which required much energy.

**I:** Uuh. By the time you was working, have you ever met a patient who was difficult to talk to because of mental health problems?

**R:** Yes **I:** Uuh. It once happened **I:** How did you behave? That time there was a nurse who was trained, and a strong man. There was someone else who was trained and helped, and managed to reach at mental health service in the hospital.

**I:** Uuh. By the time you was at work, have you ever met a patient who was difficult to talk to because problems related to his/her character or disabilities such listening, talking or others?

**R:** As for me it is not a big challenge **I:** Uuh. It is not challenging much because I once lived with the children with healing, seeing disability and those who live with mental health problem. Talking to them is not much challenging to me, however it may be difficult because you don’t understand each other much on the language used, but is not much challenging like someone who didn’t live with them. **I:** Uuh. What does it not become challenging to you? And, how do you handle it? By the time I was working there, they taught us how to use signs language it is just that as you spend time without going there you forget them, but they mostly come from the institution I lived in. **I:** Uuh. At the stadium, I know most of them. There is also someone who comes knowing how to write and write them, and get to know how to help him/her. Apart from using signs, there is someone who doesn’t know how to write but as you speak to him/her eyeball to eyeball he/she understand what you are saying. Yes it requires much energy, it is not like talking to someone who hear or talk but I try my best. It should be better if a person can receive training related having conversation with those people with hearing and talking problem.

**I:** Uuh. While you was working, have you ever met a patient who was difficult to talk to because of the difficult personality of the patient? Here I am talking about personality

**R:** It sometimes happened but it is challenging when you are starting. There is someone who doesn’t want to talk and when you ask like: What are you suffering from? The response is that: I am suffering from a headache only **I:** Uuh. It is challenging when you are starting but you try to enter into the conversation by trying to see if you can have unity or feel comfortable to each other and see what you wanted to achieve. Those kind of patients sometimes exist.

**I:** Uuh. Is it necessary to talk to the patient about the sickness you think he/she has?

**R:** Yes **I:** Why do you think is necessary? It is important because he/she is not aware of what you think he/she has. It is important because when you talk to him/her get to understand the sickness, the consequences caused by illness, and this helps taking the decision of seeking treatment. I think it is important of talking to him about it.

**I:** What can you tell the patient, in case you are not able to know the problem he/she has?

**R:** In case you are not able to know it well? **I:** Uuh. Like what? **I:** Like the time you try to do your best, by trying to do consultation to the patient but at the end you find that you are not able to know what the patient is suffering from, what can you tell him/her? You can tell him/her that as you tried you didn’t see the sickness, and if is necessary you give him/her a transfer by sending him/her to other with more knowledge or other materials you don’t have in order to try their best also.

**I:** Uuh. Based on medication the nurse prescribe, is it important to explain to the patient the type of medication prescribed, how it function, how it is taken, side effects?

**R:** It is important **I:** Which importance? It is important because there is a time you inform the medication prescribed, and as you have a conversation he/she also tells you the medications he/she doesn’t take. You may prescribe and when you give to him/her tells you that he/she doesn’t take that type of medication. What happen when you take it? And tells you to change it. There is also the time you explain function of the medication, for example hydroxide can cause stomach problem. Sometimes when you prescribe it to the patient you explain its role but you also say that it can cause stomach problem. It is better to first of all take tea before you take it, and when you find it challenging do this. It means that this helps to know how to take the medication, and how to behave in case there is a problem. It is important to explain the medication and its side effects. **I:** Do you that all the time? There is a time you explain and the time he/she comes while you are tired, and there is also a time you don’t explain much because you don’t have enough time and make you not do it the way it should be done.

**I:** Uuh. Some of the patient in Rwanda think that they don’t receive enough information about medications as you said. **R:** Uuh. Based on your experience or what you see, is it like that?

**R:** Based on what i see, they have a reason to say that, **I:** Uuh. For example we mostly tell the patient to take medications 3 times per day, by taking it in the morning, at twelve and night. What is it morning? What time is it twelve? And what time is it night? It means that in that case the patient doesn’t understand the timing of taking medications. We just say take one in the morning, another one at twelve, and other one in the night, he/she really doesn’t know how to take it! It means that when he/she forgets and remember at nine he/she will take it at that time, and when he/she remember at twelve he/she will take it that time, and when he/she remember at ten night that’s when he/she will take it. Honestly that medication is not taken the way it should be, they have a reason to say that they don’t receive enough information. **I:** Based on how you see, what cause that? It depends on the person.

**I:** Uuh. Does your functioning system or the situation you work in prevent having a good conversation between you and the patient?

**R:** The way we live? **I:** Eeh, it means the situation you work in or functioning system here, is there a time it prevents having a good conversation between you and the patient? The situation we work in? **I:** Uuh. You may feel uncomfortable as it happens few minutes ago, as we were at the other side we were uncomfortable because it was noisy! We may be sitting here while others are sitting behind us and find out that the windows are open, in that case find out he/she think that if I tell him/her everything they will listen to me or if I speak loudly. It is possible.

**I:** How do you handle the situation of the patient who wants to be transferred at the hospital while you think it is not important?

**R:** When he/she asks? **I:** Uuh. I first do what I think should be done, and if it doesn’t work I have to transfer or explain to him/her the type of the patient who should be referred. For example if he/she comes telling you that he/she suffering from the stomach I want to meet the doctor, and ask if he/she ever seek treatment and say no this is my first time. And also ask if he/she wants to take a medical test and say no, while you see he/she has stomachache and is the first time seeking treatment. In this case you first need to have a conversation, and convince him/her that it is not urgent to be referred to the hospital, he/she may also take medication given and get well till he/she listens and take it. But when he/she refuses, you refer him/her.

**I:** Uuh. Are there problems you see related to having conversation about health in Kinyarwanda?

**R:** It is challenging because you search Kinyarwanda term to use and don’t find it.

**I:** Uuh. The fact you did your studies in French or English, is it a challenge having a conversation with the patient in Kinyarwanda?

**R:** It is all the same. I don’t know if I can say that it is a problem, because having a conversation in Kinyarwanda is not much challenging. You may miss one words in the midst of words you were looking for, it is mostly due to those other languages but it is not an obstacle.

**I:** Uuh. : What do you do when you have to explain medical terms which are in French or English which does not have Kinyarwanda terms?

**R:** I do ask thoughts to my colleague, by asking how do we call this term in Kinyarwanda? And get helped. “Laugh” **I:** Uuh, can you give an example? What did I meet today! Today I received a boy who had tumefaction. I tried to ask my colleague and didn’t get the term I don’t know how we can call it in Kinyarwanda. As you can see it happens, if you know about it please explain to me. **I:** There is no specific term of it in Kinyarwanda.

**I:** Are there other challenges you experience by having a conversation with patients who come to you, we didn’t talk about in our conversation?

**R:** None, we spoke about it. Except someone who comes very ill and the one who is faking his/her sickness that’s what I can say.

**I:** What can be done to improve the knowledge you have in having conversation with patients?

**R:** It is trainings.

**I:** Patients are different, which means you also have different ways of having conversation. How do you relate the knowledge you have and the way patients have conversation?

**R:** I use the knowledge by receiving the patient without relating him/her to another patient. Each patient who enter you need to feel that is a new patient, and create an environment of having a conversation with the new patient. You don’t need to have the same conversation with everyone who comes. As for me what helps is that whoever comes I feel that is a new patient.

**I:** What can be done so that the nurse can help the patient have a better conversation in the consultation room?

**R:** Unless he/she receives few patients, and have enough time. **I:** Either they are many or few, what can the nurse do to have a better conversation with that patient? It’s receiving him/her with care. When you receive him/her with care, he/she feels comfortable of talking to you and have a good conversation. **I:** Uuh. Since you received him/her well, even the conversation goes well.

**I:** How do you handle the situation where the patient cry?

**R:** When he/she cries, I try to counsel him/her, by telling him/her that he/she is not the only one going through that situation, and make him/her feel that it happens and goes back home feeling that he/she is not the only one in that situation and also show ways to be followed so that the problems can be solved or live with them strong and healthy.

**I:** Uuh. Is it necessary to support the patients who come to you, by dealing with emotions caused by the sickness they have?

**R:** Helping dealing? **I:** Eeh, with emotions caused by the sickness they have? Yes **I:** How important is it? It is important because they help them have acceptance, and when he/she has a problem may start crying by telling you about it. **I:** Uuh. When you make her understand it enables him/her to have acceptance and life continue. If a person is living with HIV/AIDS and once he/she think about it become stressed, you need to help him/her have acceptance and live with it for a long time.

**I:** Is there something you do to make sure that the patient understands well what you are saying?

.**R:** Yes **I:** What do you do? When you are together, I mostly ask about it when I finish. I ask if he/she understand the reason I prescribed a certain medication? And say yes. It also depends on available time. If you have enough time, have a conversation about it, and also ask if he/she understands, it helps. **I:** Uuh. You also need to know if he/she understood it and repeat it or give him/her enough time you can use all strategies because one strategy cannot be applied to all.

**I:** Why do you do when it is done?

**R:** I do it because it helps. I am able to know that is living knowing this, that what make me do it. There is someone you give medication and when reach at the door comes back asking this: Nurse you said that I should also take this at twelve? So when you ask if he/she received the medication, and tells you that I will be taking this like this it helps you to know if he/she really heard what you said.

**I:** What should the nurse do to help the patients participate in the treatment they receive?

**R:** Participation how? **I:** Uuh. How? **I:** Participating in the treatment they receive? Yes **I:** Uuh, how? They should participate, as you know malaria is the big number of the patients received here, if you tell someone that has malaria and you had a conversation about it, may go home knowing what cause the malaria and also participate in the community in order to be prevented from getting it soon. I think it is important to explain and also make him/her participate. **I:** Uuh. You may also receive a mother with a malnourished child, if you explain she may go and start cultivating vegetables or do other things which help the child to grow well.

**I:** Is there something else you would to add on the conversation we had?

**R:** Add? **I:** Uuh. I don’t know what I can add on that because we said much, what I can add is that it is helpful to have conversation with the patient, and every nurse should make it a habit of having a conversation with the patient and receiving him/her well, since as we have a good conversation it also helps getting the information needed and give him/her all information you want to communicate. That’s what I can add, I don’t know if you also have another question for me which may not be among what was on the paper? As for me I don’t have something else.

**I:** The last question I am asking is that: Do you think are other questions we should have asked which are helpful in having a conversation between the patient and nurse or about the questions we asked, are there others you can add on which may make the conversation go well between the patient and the nurse?

**R:** You spoke about everything. “Laugh”

**I:** Uuh. [*Name*], thank you very much!

**R:** Thanks
