# Supplementary material for: Providers’ perceptions of communication with patients in primary healthcare in Rwanda
Source: PLoS One. 2018 Apr 4;13(4):e0195269. doi: 10.1371/journal.pone.0195269 (PMC5884556; doi:10.1371/journal.pone.0195269)
Supplement: S1 Dataset — (ZIP) [file pone.0195269.s001.zip › S1 Dataset/PPC-Provider 5.docx]

**PPC-Provider 5,**

I: Interviewer, R: Respondent

**I:** So, I thank you once again for sparing your time with us so that we have a conversation. I’d like to start by asking you, “Can you tell us a little bit about the conversation that a patient has with the health care provider in the consultation room at the health center?”

**R:** This is the first thing you do: as soon as the patient comes in, you welcome them in and they sit down and you start the conversation*. [****I****: Huh, can you speak up please?] [Both interviewer and respondent laugh at the same time].* Once a patient enters in the consultation room, you welcome them in and then you start talking; while talking you ask them why they have come and then they tell you how they are suffering; usually people who come in the consultation room are patients. So, you ask them about their illness, how they are feeling, when their illness started. After they have told you all that, you ask them about their everyday life. If the patient is married, you ask them how the relationship is between them and their wife or husband, how their children are doing – if they have produced some – and you ask them about their everyday life in their community. You ask them all those questions while treating them at the same time. If they need medical tests, you write a recommendation of the tests and they go to the laboratory. They wait for their tests results and when results are found, you help them according to what you have found and after that, you discharge them and then you orient them where they have to go for other services.

**I:** Yeah, no problem. What is the role of the conversation that patients have with health care providers in your work that you do in the consultation room at the health center?

**R:** The importance it has for myself?

**I:** No, what role does the conversation between the health care provider and the patient play in the work that you do in the consultation room at the health center?

**R:** It’s that we are able to know problems that community members have. We get to know if they are experiencing a pandemic or not, and then we take measures according to what they have told us.

**I:** Is there another importance of the conversation that you have with the patient in the consultation room at the health center?

**R:** I don’t think I can add something.

**I:** Okay. Tell us in full details what the best conversation with patients looks like.

**R:** In my understanding, when you have been able to engage a patient in conversation, you get to know their problems and their life. Therefore, as the health care provider you know what you have to do so that community members have good health. I don’t know if I am not going off the subject?

I: No problem. So, in your mind, how do you describe the best conversation between a health care provider and a patient?

**R:** It depends on how you have welcomed them. If you have welcomed them well, they tell you everything to the extent that they feel relieved and sometimes you become the answer of the problem that they have.

**I:** Isi it necessary that health care providers have enough knowledge about conversing with patients?

**R:** It is necessary.

**I:** Why is it necessary?

**R:** If you have been able to know problems that someone else has, you will be able to help them better; because if you haven’t been able to know what problems that someone else has, you won’t also be able to help them.

**I:** Why do you need to talk to patients in a kind way?

**R:** It enables me to become the answer for their problem; this also helps us to know how our community stands.

**I:** Do you think that the best conversation that you have with patients can help you to improve the health care or the way you care for the patients?

**R:** Yes. [***I:*** *How*?] When you have been able to converse with a patient, they tell you what goes well and what does not go well in your services and therefore you know whether or not there are things that you have to amend.

**I:** You know whether or not there are things that you need to amend. Can you elaborate on that point?

**R:** For instance, we may not be giving good care to our patients, which might be causing many sick people to not come to seek out health care. As a result, many sick people may be staying and even dying at home, and probably causing many illnesses and deaths. In that case, when you converse with a patient and that they openly report that issue, we blame ourselves and say “This is the reason why people are not seeking out health care, so we must correct such and such things in our services”.

**I:** Huh, well, maybe I want to repeat the question. Is there another way that you think the conversation that you have with patients can help you to improve the health care?

**R:** I am afraid I think I will give you the same answer.

**I*:*** No problem, let us move to the next question. What are the benefits of the best conversation that you have with patients?

**R:** The benefits of the conversation that I have with patients? [***I:*** *Huh*], it’s that they feel comfortable and then they tell you their problems.

**I:** Do you think that having enough knowledge about conversing with patients can help you to give better health care to patients who come to see you?

***R:*** *It can help patients who come to see you?*

I: Can it help you to give better health care to patients who come to see you?

**R:** Knowledge…repeat that question?

**I:** Let me repeat it. Having enough knowledge about how to converse with patients, do you think this can help you to give the best care to patients who come to see you?

**R:** Yes.

**I:** How?

**R:** When you talk with a patient about things that you know, they benefit from that. When you have the knowledge that you are sharing with the patient, they come to know that there are things that they have to avoid doing and things they have to start doing.

**I:** Here I am focusing on the nurse’s knowledge of engaging patients in conversation. The nurse’s knowledge of conversing with patients, can it help you to give better health care to patients who come to see you?

**R:** To help the nurse or the patient?

**I:** To help the nurse. Let me put it in a different way. The nurse’s enough knowledge of how to engage patients in conversation, can it help the nurse to give better care to patients who come to see them?

**R:** Your question is wrapped up in so much verbiage that it is hardly understandable. Yes.

**I:** Yes. How?

**R:** I said that when you have enough knowledge, what you share with other people is also enough.

**I:** Here, I would like that you focus on the knowledge of how to converse with patients.

**R:** Well, what is that knowledge that you are talking about? [***I:*** *That is communication skills*] Aha, when you have the knowledge of what you say and when you are able to explain it to someone else, they benefit from it so much, they easily understand what you tell them.

**I:** Did you ever receive a training about how to improve the communication with patients?

**R:** What type of training?

***I:*** *Training about communication skills*

*R: No.*

**I:** Why?

**R:** How can I know?

**I:** Wasn’t it planned for? Is it important?

**R:** What?

***I:*** *Having that kind of training.*

*R: Yes, it is important.*

**I:** Why?

**R:** Because when you have the knowledge about something, you are able to share it with other people. You say what you know.

**I:** I am talking about the skills, I am always talking about the skills in conversing with patients at the health facility.

**R:** It enables you to talk to patients in a kind way and therefore they feel pleased.

**I:** On a scale of 1 to 10 points, how much points would you give yourself in relation to your skills in conversing with patients in a proper way?

**R:** I would give myself 8 points.

**I:** And how about the remaining 2 points? Is there a gap in your skills that prevents you from conversing with patients in a proper way?

**R:** The reason why I cannot give myself 10 points is that it is impossible to talk to all patients who come to see you in the proper way.

**I:** Where is the problem?

**R:** You cannot have fifty or more than fifty patients and be able to converse with every single patient for as enough time as required. In such situations, you skip talking about some things in order that you are able to see each one.

I: But that is an issue that can be caused by the big number of patients. But here I am talking about your communication skills, your ability to engage patients in conversation; do you have some problems in that domain?

**R:** If there wasn’t the problem of a big number of patients you would talk to all patients in a required way and you would score ten points.

**I:** What do you think you need to improve? What do you need to improve so that you score ten points out of ten?

**R:** What needs to be improved is that we would basically make all patients satisfied if there were many nurses to care for a small number of patients. That means, the number of staff should increase so that there are a few patients for each staff. Once the number of the staff increases, the number of patients that each staff member must care for would reduce too.

**I:** How does your collaboration with other health care providers make the conversation with patients go well?

R: Repeat the question?

I: How does your collaboration with other health care providers make your conversation with patients better?

**R:** The conversation goes well when other colleagues orient patients well. In that case, patients do not go astray nor do they lament; so they are happy when they come to see me and the conversation goes smoothly.

**I:** I am talking about the collaboration between other health care providers and you, does it play any role in making the conversation between patients and you go smoothly?

**R:** Yes, it does play a role.

**I:** Yes, explain more?

**R:** Because if a patients goes to see my colleague before coming to see me and that my colleague says, “That one is very unhelpful”, the patient would come to see me already knowing that I am very unhelpful and that I won’t help them at all. As a result, the patient may hide some things and don’t tell me about them.

**I:** Do you think it is useful to ask patients if they came to seek health care expecting to get something in particular?

**R:** Yes, it is necessary.

**I:** Why?

**R:** Because if they come to seek health care with the belief that they will recover, for sure they will. But if they come with the belief that no-one can help them, they cannot get well no matter what medication you can give them.

**I:** So, is it useful to ask them that question?

**R:** It is useful.

***I:*** *Why*?

R: So that you can see if they are really satisfied.

**I:** Is there anything that you do to know if patients need to know more information concerning their health?

**R:** Yes

***I:*** *What do you do*?

R: You try and ask them about things that they did not tell you.

**I:** But when you are asking them questions, it’s you who are trying to know more information about their problems. Now, here I am talking on the side of the patients. They can also need to know more information about their health or their illness in general. So, is there anything that you do to know whether or not the patients who have come to see you need more information?

**R:** Yes, you can treat them after they have told you the signs they see and how they are feeling. Sometimes they go home knowing that you have treated them but without knowing what you have treated them for. But when you explain to them what you have treated them for, they get more information.

**I:** Why do you do that?

**R:** You do that in order to help them know what they are suffering from.

**I:** Do you think that it is necessary to explain to the patient everything that they need to know?

**R:** It’s necessary.

**I:** Why is it necessary?

**R:** It is their right to know everything that is done for them and whatever will be done for them.

**I:** Is it important to let patients be involved in making decisions regarding the health care that they need? Can I repeat the question?

**R:** No, I am still thinking about it. I think it is not necessary.

**I:** Why is it not necessary?

**R:** Because you might disclose a professional secret by trying to make the patient satisfied.

**I:** Can you give us an example?

**R:** An example of what I have answered you, let me give you an example. I can give pills or an injection to a patient while I should give them – I mean it can happen that I give to the patient a type of medication which I shouldn’t give them. Then, a colleague of mine can come and tell the patient, ‘Hey, did you know that this is not what should be given to you?” In that case my colleague creates conflicts between the patient and I; my colleague creates things which are not good. So, if you think that the medication cannot have any adverse effects, I don’t think there is necessity of letting the patient know about it.

**I:** Here we were talking about the patient’s involvement in the health care that they would like to be given.

**R:** No, it is not necessary. A patient may want to be given an injection when I see that a pill would be better for them.

**I:** Why do you think it is not necessary?

**R:** They can get better if I give them pills, and they can also get better if I give them an injection. However, I may find out that if I gave them pills, that would be the best treatment – injections are also good medications but we avoid to give them to patients in order to minimize the risks.

**I:** When a patient has choices regarding health care, should a health care provider take their choice into consideration?

**R:** Yes, they should take that into consideration.

**I:** Why should they take it into consideration?

**R:** It’s the patient’s right and it must be respected. If you think that there will be no unwanted effects, you can respect the patient’s right and let them make a choice.

**I:** When a health care provider shows their emotions, how can the emotions have an impact on the conversation that they are having with the patient?

**R:** Repeat the question for me?

**I:** When a health care provider exhibits their emotions like happiness, sadness, anger and so forth; how can this fact of showing these emotions have an influence on the conversation that they have with patients?

**R:** Well, is there a question for which I should answer “yes” or “no”?

***I:*** *No*

R: The health care provider should always remain childish before the patient.

**I:** Why?

**R:** So that the patient feels free and comfortable.

**I:** Can you explain that more?

**R:** The health care provider should show that they are feeling the patient’s pains.

**I:** Is it acceptable that health care providers show their happiness or sadness when they are with patients?

**R:** Yes.

**I:** Why?

**R:** Always you have to be sympathetic for the patient.

**I:** Do you think that patients can feel worried about telling their health problems to the health care providers?

**R:** Yes. Because if you do not make them welcome and if they are not feeling at home, they cannot tell you their problems.

**I:** Then, what can be done?

**R:** Health care providers should remain humble and kind towards patients so that they can feel comfortable enough to tell them about their problems.

**I:** What is your experience with patients whom you think have low level of education, specifically the illiterate ones?

**R:** Yes, most of the time they don’t know what you ask them. For instance you can ask them “When were you borne?” and they tell you “I was born when sorghum was being harvested” or they can say “I don’t know”.

**I:** What do you do in that case?

**R:** When they have a national identity card, you ask them for it and you look at it.

**I:** How do you use your communication skills to engage patients of that category in conversation?

**R:** You talk to someone who studied in a particular way. For an illiterate person, you try to ask them questions that they are able to answer. There are questions they cannot be able to answer but you try and ask them questions which are related to their level of knowledge.

**I:** Do you want to mean that you make yourself illiterate also? You make yourself someone of their level?

**R:** Let me give you an example. For instance if it is that one who replies “I don’t know” when you ask them when they were borne, you manage to understand each other so that you give them the service they need even if they don’t know when they were borne.

**I:** How does the Rwandan culture influence the conversation that you have with patients?

**R:** For example when you are talking to those people who are illiterate, the ones who never went to school, you have to speak in a language that they understand. You try and do things that they can understand, you make sure you do not bring in things that they can’t understand. For instance if you use a French word, they say “The health care provider is looking down on us because he/she studied. That one is not in our category.”

**I:** In your opinion, what reasons do usually hamper the conversation on the side of the patient?

**R:** When you have not done what?

**I:** From your perspective?

R: I got the question.

**I:** You have understood it? Okay.

R: If you don’t act in a kind way…[*interrupted*]

**I:** You are talking about the health care provider, aren’t you?

***R:*** *Yes, I am.*

*I:* For me I am talking about the patient. What are the reasons that usually hinder the conversation on the side of the patient?

**R:** Yes. When you do not show them that you are kind and that they are welcome, they feel that you won’t also treat them. They can’t tell you what they should tell you.

**I:** Well, that is what I was talking about. That is for the side of the health care provider.

**R:** Huh, if you are not kind towards the patient, they feel that you don’t care for them and that whatever you tell them is useless; therefore they keep silent.

**I:** That means, a patient may remain silent and as a result the conversation becomes bad.

**R:** A patient can keep silent due to their problems or because they are not comfortable to talk with you.

**I:** Are there other factors arising from the patient’s side that you think can negatively affect the conversation between the health care provider and the patient?

**R:** If patients have problems but don’t want to talk about them; they can keep silent.

**I:** Are there other factors?

**R:** I don’t see other factors.

**I:** Now, on the side of the health care provider, what are the factors that usually affect negatively the conversation with the patients? Now it’s on the side of the health care provider.

**R:** If you do not make the patient welcome in the beginning, the conversations do not go well as a result. If you are busy with other things, they see that you are not caring for them.

**I:** Are there other factors arising from the health care provider’s side that can negatively affect the conversation with the patients?

**R:** I don’t see any other factors.

**I:** And then, on the side of the health center’s work conditions; from your perspective, what are the reasons that usually affect negatively the conversation between health care providers and patients? Now lay an emphasis on work conditions at the health center?

**R:** Which make the conversation go in a bad way?

I: Yes, due to work condition at the health center?

R: When the health center does things that patients dislike.

**I:** Can you explain more?

**R:** No.

**I:** Can you give examples of things that are difficult to tell patients who come to see you?

**R:** Yes. For instance, it is difficult to talk to a mentally ill patient.

**I:** There, you see that it is the problem of the patient’s disability. Are there some things which you think are so difficult to tell patients about?

**R:** You mean that the conversation is difficult?

**I:** Certain things, the ones that make you think “I may have difficulty telling these things to the patient”?

**R:** Such things aren’t there.

**I:** During your work, did you ever receive a patient whom it was difficult to talk to as a result of a certain problem?

**R:** Huh.

**I:** What was the problem?

**R:** As our health center is built near schools, they recently brought a female student who was so calm that she couldn’t talk even if you would pinch her. The problem usually happens for example when female students are received by female health care providers; even if you pinch her, she cannot react. So, for the previous case when a male health care provider appeared, the student opened her eyes and then she started to talk to the male health care provider and she even told him her problem whereas she had refused to talk to me; she had even showed me that she was not alive and that probably she was not breathing.

**I:** What do you do in such cases? Or what did you do at that time?

**R:** I asked the people who had brought her and they reported that she was affected like that. They didn’t explain to me her illness. So, what I did was to call my colleague to come and help me. When I went out to bring a serum, the student heard the male’s voice and she started to talk. When I came, they were already talking to each other. So, I told my colleague to continue to care for her and I went to do other work.

**I:** In your work, did you ever receive a patient with whom it was difficult to have a conversation due to their mental illness?

**R:** Yes, I received mentally ill people.

**I:** What did you do?

**R:** I wrote a transfer and I called an ambulance and it took them to [name] hospital.

**I:** Did you ever receive a patient with whom it was difficult to have a conversation due to their disability like deafness, dumbness, blindness and so forth?

**R:** Yes, I did receive some.

**I:** What did you do at that time?

**R:** The one I received was not able to speak, but he were able to understand. They used signs and I knew some of the things they are saying. Additionally, the patient had come with a small child, so I asked the child everything that I wasn’t able to understand.

**I:** Did you receive a patient with whom it was difficult to have a conversation due to their nature or personality?

**R:** Who were not mentally ill?

**I:** Yes, who were not mentally ill but who had a difficult personality.

R: I received one. He only ordered what had to be done for him. He would only said “I don’t want this, this is what I want.”

**I:** How did you handle that case? What did you do?

**R:** At that time I did what I had to do. I analyzed what he was telling me and I only did what I found good and I ignored what I found bad.

**I:** Is it necessary to tell patients about the illness or problems that you thing they have?

**R:** It is necessary.

**I:** Why is it necessary?

**R:** They get to know whether or not their illness is curable.

**I:** Does it have any other importance apart from that?

**R:** It has another importance. It helps the patients to know how they have to behave in relation to their illness. Additionally, their family knows how to care for them. And you also know that the patient you are treating will get cured or not.

**I:** What could you tell the patient if you weren’t able to identify their problem?

**R:** I would tell them that I am going to refer them to someone who can give them the best care.

**I:** Concerning medications that the health care provider prescribes, is it useful to explain to the patient the type of medication you prescribe for them, how the medication works, how it is used and any side effects that it may have?

**R:** It’s necessary.

**I:** Can you explain it more?

**R:** It is necessary because you have to explain to them about the medication that you are giving them; that means you tell them how they will use it, the unwanted effects that they may experience or not. Once they know this, they don’t feel worried when they experience the side effects. Additionally, it is the patient’s right to know all that information.

**I:** Some patients in Rwanda report that they do not receive enough information about medications. Based on your experience, is that true?

**R:** Yes, it is true.

**I:** From your perspective, why does that happen?

**R:** There are health care providers who treat patients but do not tell them what they are treating them for, nor do they tell them why they have given them those medications. They just give them the medications saying “Go and use them”. That is the truth.

**I:** What is the reason behind that, in your opinion?

**R:** Not explaining to the patient about the medication?

**I:** Yes, not giving to the patient enough information related to the medications.

**R:** It depends on one’s personality.

**I:** Whose personality are you talking about? The health care provider’s personality?

R: Huh

I: How so?

**R:** Because the health care provider feels that the patient should not know which medication is for which illness. But this is not good. A patient has the right to be given all the information about everything that is going to be done for them.

**I:** Do some of your work conditions negatively affect the conversation that you have with patients? ***R:*** Repeat the question?

*I:* Some of your work conditions or situations at work, do they negatively impact the conversation between patients and you?

**R:** Yes.

**I:** Can you explain that in more details?

**R:** The patient and you may be sitting somewhere talking when there are other people behind you who are hearing what you are talking. In that situation, the patient decides to not say some things lest that those people may overhear what the patient is saying.

**I:** What do you do if a patient requests to be referred to the hospital when you think it is not necessary?

**R:** In that case you first of all explain to them why it is not necessary. If they understand they stay there; if they do not understand, you refer them because it’s their right.

**I:** Are there any challenges that you have regarding the conversation about health in Kinyarwanda language?

R: Problems of what?

I: Are there any challenges that are associated with the conversation about health in Kinyarwanda?

**R:** There aren’t.

**I:** The fact that you were taught in French or in English, is it a challenge when it comes to properly conversing with patients in Kinyarwanda?

**R:** No, it isn’t a challenge.

**I:** What do you do when you have to explain French or English terms that are used in health care domain? The terms which don’t have equivalents in Kinyarwanda?

**R:** Terms which don’t have equivalents in Kinyarwanda?

**I:** Huh, which don’t have equivalents in Kinyarwanda.

**R:** You say those terms as they are. Sometimes patients do not understand them. Of course they do not understand them but you just say them as they are.

**I:** Can you give me an example?

R: Of those terms?

I: Maybe one example of such terms.

**R:** I’ afraid, I don’t find a quick example for that! Well, for instance ‘creatinine”. I don’t know its equivalent in Kinyarwanda.

**I:** When it turns up necessary that you explain it to a patient, what do you do?

**R:** When I have to explain what “Creatinine” is?

I: Yes, that medication.

R: I tell the patient that it is intended to see if their kidneys have problems or not.

I: That is enough. But it means that when the patient doesn’t ask you to explain it to them, you tell them the term like it is?

**R:** You mean that I tell them just creatinine?

**I:** Huh, you told me that you say the terms as they are and that patients don’t sometimes understand some of those terms.

**R:** It would be better if you know their equivalents in Kinyarwanda; that would help them to understand. They would understand everything that was done for them.

**I:** And when there is no equivalent?

**R:** In that case, they just keep the terms as they are even if they don’t understand what they mean.

**I:** Are there other challenges that you encounter with while you are conversing with patients?

**R:** Yes, a patient can tell you, “Although I have come to seek health care, I haven’t eaten for three days. I have no food at home” and you cannot find things to give to all patients. They can even tell you, “I am homeless.” Can you build a house for them in that case?

**I:** In what ways is that a challenge concerning the conversation that you have with the patients?

**R:** If you treat a homeless person, they will keep feeling worried, they will never feel relaxed.

**I:** And what consequence does that have?

**R:** Consequences are unavoidable; the patient will always be sickly as a result of problems.

**I:** To the extent that this can negatively affect the conversation that you have with them?

**R:** It can impact it negatively. The patient can tell you “Where shall I go after receiving the health care? My problems are endless!”

**I:** What can be done to improve your skills in conversing with patients?

**R:** Training.

**I:** Can you explain more?

**R:** I need training about conversing with patients. I also need enough time.

**I:** Patients are different. How do you use your communication skills to handle different communication styles of patients?

**R:** I gave you an example before. The way you talk to an illiterate person is different from the way you talk to someone who studied. There are things that you can say and the one who studied fails to understand but the one who studied does understand.

**I:** You mean that the one who studied has difficulty understanding what you say or it’s rather the illiterate one who fails to understand?

**R:** The illiterate is the one who has difficulty understanding. They have their own perception. But even if someone who studied may have their own perception, they can still understand what you say. They can disagree with you but having understood; they can be stubborn in other words.

**I:** What can be done to help patients feel more comfortable and talk to the health care provider during consultation?

**R:** The one thing that is very important is that the health care provider has enough time and possesses skills in making other people talk; because there are people who have knowledge but who don’t know how to talk to other people; such people just say “yes” to everything.

**I:** Is there a way that the health care provider can help patients to talk with him in a better way?

**R:** To talk to the patient?

**I:** Yes, it means that the health care provider can help patients converse with them feeling more comfortable during consultation.

**R:** Yes.

**I:** Explain in more details?

**R:** The patient may be hiding some problems; so the health care provider must guide the conversation in such a way that the patient says those problems. But this depends on how the patient appears to the health care provider.

I: If I got you correctly, the health care provider must engage the patient in conversation?

**R:** Yes, he must engage the patient. I can’t agree more.

**I:** What do you do when a patient cries?

**R:** You let them cool down first of all. If they want to cry, you cannot stop them, you first of all let them cry and after crying you approach them and ask them what has made them cry.

**I:** Is it useful to help patients control their emotions resulting from their illnesses?

**R:** Huh.

**I:** How is it useful?

**R:** Because, for example that one who is crying, if you do not let them cry first, they cannot tell you their problem. You first of all let them cry and after crying, you call them and ask them why they cried. That’s the answer I have.

**I:** Is there something that you do to make sure the patient understands what you are saying?

R: You say what?

I: Do you do something in order to ensure that the patient who has come to see you understands well what you say?

**R:** Yes.

**I:** What do you do?

**R:** If you ask them something and that they give you an answer which is related to what you have asked them, it means that they have understood. But if you ask them “Do you understand well?” and they don’t answer you, you may wrongly tell yourself that they understood even if they are not answering you while it may be because they are not really hearing you.

**I:** What happens in such a case? What do you do?

**R:** When you find that they are not hearing?

***I:*** *Huh.*

*R: Me I don’t know how to talk to a deaf person.*

**I:** I am not talking about the hearing impairment; I am talking about the ability to understand what you are saying.

**R:** You ask them to repeat what you say and they repeat it.

**I:** Why do you do that?

**R:** In order to know if they have understood or not.

**I:** Should a health care provider make patients be involved in the health care that they receive?

**R:** A patient to play a role in the health care that they receive?

**I:** Yes, thanks to the help from the health care provider.

**R:** That question is too difficult to be understood.

**I:** A health care provider, should he/she help patients to participate in the health care that they receive?

**R:** Yes.

**I:** How can they do that?

**R:** They can explain to people that one needs to have a valid mutuelle, one has to pay for mutuelle in order to seek health care.

**I:** Why is it necessary?

**R:** In order that people are able to pay for the health services that they are given.

**I:** The example you have given is concerned with mutuelle and health care and so forth. But patients can be involved in the health care that they receive in many ways.

**R:** Huh, the health care provider can teach the patient and inform them about why they have been given specific health care and what they have to do after receiving the health care.

**I:** Why is that necessary?

**R:** It is necessary because if you do not teach the patient before giving them the health care, they can go and just relax and the treatment you gave them becomes useless.

**I:** Do you have anything else to add on what we have discussed?

**R:** No.

**I:** Do you think there are other questions that we should ask about how to improve the conversation between patients and the health care provider?

**R:** No other questions.

**I:** [*Name*] thank you so much.

R: Okay, thank you!
