# Supplementary material for: Providers’ perceptions of communication with patients in primary healthcare in Rwanda
Source: PLoS One. 2018 Apr 4;13(4):e0195269. doi: 10.1371/journal.pone.0195269 (PMC5884556; doi:10.1371/journal.pone.0195269)
Supplement: S1 Dataset — (ZIP) [file pone.0195269.s001.zip › S1 Dataset/PPC-Provider 2.doc]

**PPC-Provider 2**

I: Interviewer, R: Respondent

**I:** Let me start asking you ‘what can you tell us about the conversation a patient has with a healthcare provider in the consulation room at a health center?’

**R:** The conversation between a patient and a clinician/ healthcare provider at a health center should be good… A challenge we encounter is the shortage of time due to many clients waiting for our care. Therefore, that important conversation which is needed is not really done.

**I:** How important is the conversation between a patient and a healthcare provider for your work in the consultation room at a health center?

**R:** Importance? [**I:** *Huum]* If you conversed with a patient, here in the consultation room, it allows knowing the whole information about the patient so that you are able to help her/him accordingly.

**I:** Huum. Ok. Tell us in details how you think about a good conversation between a patient and a healthcare provider looks like.

**R:** A good conversation - suppose myself I go to seek care and have a conversation with my healthcare provider - we should feel comfortable each other, familiar one another so that the patient feels talking everything. Therefore, such a conversation will be of a great value.

**I:** If I understand well, do you mean the conversation between a patient and a clinician being free and comfortable?

**R:** I think a conversation between a clinician and a patient should be good because it is very important for the client (patient); and the clinician will be able to know the health problem of the patient.

**I:** Is it necessary that clinicians have enough knowledge/skills about conducting a conversation with patients?

**R:** Yes, it is. [**I:** *Huum. Why?]* It helps receiving well the client (patient) and provide care accordingly.

**I:** Huum. Do you think a good conversation you have with a patient can help in improving the clinical/ medical care?

**R:** Yes, I do. [**I:** *Why? Or how?*] If I find an opportunity of welcoming a patient and having a conversation with her/him, it is said that it creates a hope for the patient. That hope is like a half for the patient to recover from her/his illiness. With such a good conversation, the patient provides enough information with no reservation and the clinician may even diagnose another illiness the patient had not thought to talk about. Then , you examine and help her/him fully with a good impact for the clinician because of the expected recovery for the patient and the hope s/he goes back home with.

**I:** What is the advantage of a good conversation between a clinician and a patient?

**R:** Well, the advantage of a good conversation with a patient is to discover what the patient may have hidden when talking. In fact, the client may come for healthcare and hide some health problems in case you do not have a conversation while you should have have known it within such a conversation.

**I:** Do you think enough knowledge/skills about conducting conversation with patients may allow you more help the patients who come to you?

**R:** That’s true. [**I:** *Huum. How? Could you explain to me?*] Because, for enough knowledge…for example…the way I should talk to a patient I may prevent her/him from talking some problems. For example, I may hurt her/him within feelings/emotions while if I knew how to conduct a conversation with a patient, I would guide my patient and get enough information from her/him so that I provide a good service/care.

**I:** Have you ever been trained about improving the way you talk to patients?

**R:** No, I’ve never been trained about it. [**I:** *Why?*] But when I was a student…, [**I:** *at school*.] Huum. [**I:** *Huum. Ok. Do you think it is important*?] It’s important to know how to do it. [**I:** *Why*?] If I know how to do it, I will be able to have a quality conversation with a patient, and s/he will have a hope (of recovery), and I will have enough information from her/him to enable me or to guide me to the ways of helping her/him for the illiness s/he is seeking care for.

**I:** Huum. On a scale of 1 to 10, how are you prepared to have a convient conversation with the patients coming to you?

**R:** That’s what I told you at the beginning that due to many patients we receive and the shortage of nurses at a health center, we do not have such an opportunity/time. However, if I find time, I wish I could rate up 10 out of 10. [**I:** *Huum. Are you currently at which rate*?] Now, the rate is still low because of the issue of many patients we receive with few staff. [**I:** *what do you mean with low rate: 3, 4 out of 10*...?]. I do not even reach 4!

**I:** Then, apart from that issue of time, maybe now we are talking…Let me come back especially on your skills or your knowledge about conducating a conversation with patients: do you think there is another gap which can prevent you from having a conversation with patients conveniently?

**R:** There is no other problem except the one of many patients who come for seeking care while there is a shortage of nurses. For me, that’ s the only issue.

**I:** How does your collaboration with other clinicians, with your colleagues, influence the good conversations you may have with patients?

**R:** For the relationships with my colleagues, if I find an opportunity/ time to talk to a patient and find something unclear for me, I may call my colleague to come and help me. S/he may provide another idea and have some consensus first with our client (patient). [**I:** *Is that important for the patients?*] Yes, it’s very important for the patient.

**I:** Why do you think it’s important to ask patients if there is anything special expected for the healthcare they will receive?

**R:** Yeah, we ask them if there is any expectation because s/he should have gone somewhere else but s/he came here to seek care to me. That’s why during our conversation, I ask her/him: what is helpful for you, what is going well, what do you think it is not helpful for you? This is a guide to have a mitual understanding so that you provide a good care for her/him.

**I:** Huum. Is there anything you do to know if the patient with you wants more information about his/her health issues?

**R:** No! [**I:** *Huum! Why don’t you do anything?]* I do not think about that! [**I:** Do you think it’s necessary? ] Asking her/him? [**I:** *Asking her/ him if s/he needs more information about her/his health?*] I think it would be good and necessary. [**I:** *Could you please more explain why you think it’s necessary?*] It would be necessary if you provide the patient with information about his/her health/life, it will help her/him for prevention and take some strategies his/her bright future.

**I:** Do you think it’s necessary to explain to a patient everything s/he wants to know?

**R:** It’s necessary. [**I:** *Why do you think so?*] It’s necessary because if it’s about her/his health it would help her/him for prevention and recovery. It would help her/him recover from the illiness, and help for the prevention for other illinesses either for her/himself or for his/her family members or his/her environment.

**I:** Huum. Is it important to let patients who come to you participate in the decision-making concerning the clinical/medical care they are provided for as they want/wish?

**R:** It’s important. [**I:** *Why?*] It’s important because a patient wishes a freedom for his/her health issues. It sometimes also helps a clinician to make some decisions according to his/her feelings and the decision may be convenient. That’s when the clinician will have value when examining/treating the patient.

**I:** Huum. If the patient was to choose about the clinical/medical care s/he will be provided for, should the clinician put importance importance on that?

**R:** S/he should put importance on that. Then, s/he will evaluate, balance and see what is really helpful. Finally, the patient will need explanations from the clinician as everything will be solved by the clinician’s explanations about the challenges a patient may encounter because of the decision taken.

**I:** A clinician may show his/her feelings/emotions: In which ways does it influence the converstion between a clinician and a patient?

**R:** If the clinician shows feelings/ emotions during the conversation, it makes the patient feel comfortable with her/him. For example if the patient comes with sadness, the clinician should also be somehow sad so that it shows like you are together and the patient will be more comfortable with you and have a hope about the care s/he will receive from you.

**I:** Is necessary that the clinicians show joy/happiness or sadness while they are together with the patient?

**R:** No! [**I:** *Why?*] (*laughing*) You may be happy while the patient is sad and that may be a problem. On the other hand, if you show sadness, the patient may think you are worried!

**I:** Do you think patients may have worries/doubt of talking about their own health problems to clinicians?

**R:** That happens. [**I:** *What can be done*?] What can be done? I’ve no answer on this (*laughing*).

**I:** Huum. Ok. What do you experience while you are conversing with patients you think they are of low level of education? For example the illiterate ones?

**R:** What do I experience? [**I:** *What do you experience while you are having conversation with patients you think they are of low level of education? For example those who have never learnt how to read or write?*] It’s not… Anyway, if s/he comes…if s/he has no level of education you treat her/him at his/her level; you explain to her/him while speaking in the language s/he can understand, in a clear Kinyarwanda. There would be not any problem. Of course you will provide to him/her enough explanations: if during the conversations you notice s/he does not understand the pieces of advice, you try to explain.

**I:** Do you experience such hard situations?

**R:** Yeah, it happens. [**I:** *Huum*. *Tell me in details*] If you tell her/him…during your conversations, and you notice that s/he has maybe a problem of low level of education, you try to convince her/him. In such a case, s/he may respond to you that’s for the educated people who have money, who are employed or know how to read and wr… . You may for example tell her/him about listening or reading about…listening the news related what you are talking about, and s/he replies s/he does not have ability as s/he does not know how to read or write. Then, you ask her/him not being worry, that s/he can hear it from meetings or s/he can approach a community health worker to help about it, and the issue can be solved.

**I:** Huum. How do you match/ relate your knowledge to the conversation you have with people and patients of such a level?

**R:** How do I relate/match it? (*silence somehow long*).

**I:** Huum. How you match it: You some communication skills, then there are also those patients in the category of low level of education or who have not studied. How do you match those skills you have and the patients of that category?

**R:** I match it with the fact that I have some skills and what to provide to them and what I think it’s very helpful.

**I:** How du you match it? You understand you have some skills/knowledge, and for them, their skills are on the low level. How do you marry those things?

**R:** For matching these things, I do not understand how to do it. However, when they come, I arleady know that I have something to provide to them through the way of conversations or helping them in the other life. Therefore, if they come to seek care from me, they undoubtedly come from somewhere and reach to right place. [**I:** *Huum. undoubtedly?]* They come from somewhere and reach another place.. [**I:** *They reach to a right place*.]

**I:** How does the Rwandan culture, our culture as Rwandans, impact the conversations between a patient and a clinician?

**R:** The Rwandan culture includes having the same language. As different categories of people come, either those who have some level of education or not, either those who speak foreign languages or not; but mostly our Rwandan culture, like sharing the same language helps to understand one aother in a whatever way.

**I:** Huum. According to your perspective, what are the reasons/factors which impede a good conversation on the side of a patient?

**R:** The factors which may prevent good conversations on the side of a patient, the first one is the fact of not feeling comfortable with the clinician. The second one is being worried, like a patient may come having in mind to meet someone else; a female may come with some problems and if she notices that a clinician is male and she prefers not telling you her problems! In such a case, you have to make her not worry so that the conversation becomes good. Vice-versa, if she notices that I am a female clinician while she was looking for talking to another male, the conversation will not be good as he will not be comfortable with you.

**I:** Huum. What about on the side of the clinician?

**R:** On the side of the clinician, the challenges may be the clinician does not feel an initiative of receiving any patient whop comes. In such a case, it will no be good. Another issue as we talked about it earlier is the one of shortage of time.

**I:** Huum. Don’t you think other factors which can impede the good conversation on the side of a clinician, between him/her and a patient?

**R:** Normally, maybe there is the health of the clinician himself/herself; another factor is the knowledge s/he has, and the trainings you were talking about. I think that all and so on.

**I:** Do you mean few trainings?

**R:** Yeah.

**I:** Ok. What are the factors which may impede good conversations due to the functioning of a health center?

**R:** (silence) On the side of a health center, I think the conversations may not go well because of first all the shortage of staff. If there are not enough staff, they do not have enough time to have a conversation with patients. The time is not enough while it is the main key. This is the main challenge which exists where there are few staff while there are many patients. In such a case, they have hard work. It would be helpful if this issue was solved to improve the functioning of a health center even during those conversations.

**I:** Huum. Can you give some examples of things/items which are difficult/hard to talk about during the conversation with patients?

**R:** Examples … [**I:** *Things which you think are hard, which may be difficult for the clinician while s/he is having a conversation with patients?*] Some challenges are that a patient may come with a mind that the conversation will not do anything for her/him because of the health condition in which she is. The 2^nd^ challenge may be that one of time that clinicians are not available to talk to patients.

**I:** Huum. Is there any particular thing maybe you can say that it’s difficult to talk about it with a patients who comes for care? The one which is difficult to tackle?

**R:** Before it was not… If you talk somothing and teach it a patient, it cannot be difficult! [**I:** *Huum*.] No, everything can be taught to a patient!

**I:** Huum. Ok, ok! During your work, have you ever met a patient whom it was difficult to talk to because of a problem s/he was having?

**R:** Huum. [**I:** *What was the problem?*] … S/he is the patient who may come with some other family problems; it’s hard to make her/him well understand. Her/his illiness may have a root cause from her/his family due to for example lacking somothing from the family…. In such a case, it’s hard to convince and make her/him happy during the conversation.

**I:** Huum. How did you behave during that time? Or What do you do?

**R:** When I encounter this situation, I call upon my colleage for a support and we collaborate for the counselling. If it does not work, we give her/him a rendez-vous. Eee, it requires long time, it’s not something which can be done for one day! It requires a long-term/ periodic follow-up and conversation.

**I:** Then, when you are at work, haven’t you met a patient whom it was difficult to talk to due to a mental illiness?

**R:** Huum. I met such a patient. [**I:** *Have you met her/him*!] Huum.

**I:** Huum. What about the other health status like hearing impairment/deafness, dumbness, blindeness or others?

**R:** Sorry?

**I:** Have you ever received a patient whom it was difficult to talk to because of the above mentioned problems?

**R:** I received the one with a hearing impairment.

**I:** Then, have you ever also received/met a patient whom it was difficult to talk to because of her/his complicated temper/ personality?

**R:** Yes.

**I:** Huum. Then, among all those cases, maybe the one with a mental illiness, a complicated temper/ personality, or deafness and dumbness, how did you proceed?

**R:** Among those I met, for the one who was deaf, s/he was being accompanied and I asked information her/his caregiver and this told me about her/him. I referred her/him to hospital so that they check about this problem of deafness as it was not congenital. This problem was coinciding the other illiness. At the hospital, they treated her/him and fortunately we made a follow-up and noticed that s/he recovered from all these problems.

**I:** But here, I am talking about the conversation you have with a patient with a hearing impairment. Did you meet such a patient whom it was hard to talk to? For example having hearing impairment… How did you behave so that you communicate with her/him?

**R:** Someone was accompanying her/him and helped me with the communication.

**I:** What about the one you told me about complicated temper / personality? What did you do?

**R:** For the one who came with a complicated personality, I also called upon my colleagues and the side of her/his family for help.

**I:** Huum. What was about theone with a mental illiness?

**R:** Huum, We ask a help from his/her family; those who are accompanying her/him.

**I:** Ok. Is it necessary to clarify to a patient the problem of illiness you think s/he is suffering from?

**R:** It’s good and it’s necessary. [**I:** *Huum. Why?* ] If you finish talking about it, telling her/him about the illiness you think s/he might be suffering from, it helps her/him receive it in mind without thinking it’s an accident even if s/he was somehow worried. S/he becomes prepared for that illiness. In fact, I found it important to tell to the patient what the clinician is suspecting; I found it helpful for them.

**I:** Huum…. What can you tell to the patient if you are not able to diagnose any problem?

**R:** You explain to her/him that it’s confusing, and you give a rendez-vous for follow-up.

**I:** Huum. Concerning the medications to be prescribed, is it important to explain to a patient the type of a prescribed medication, its functioning, how to take it and some expected side effects

**R:** It’s very important to explain it to her/him.

**I:** Some patients in Rwanda think think that they are not provided with enough information about medications …Huum… Let me come back a bit on this question: you were saying that it’s important to explain about the functioning of a medication, how to take it, and some expected side effects. You told it’s important. [**R:** *It’s important*.] Why is it important?

**R:** I think it’s important because the patient goes back home … If you tell it to her/him s/he believes that the medications are related to the diagnosed illiness and s/he goes back home having a hope of recovering with those medications. If s/he told you I am suffering from here, it’s swelling; and if you tell her/him that the medication is for that swelling, s/he goes back how with hope to be recovered from that part of the body because of the realted medication. [**I:** *Huum. Do you mean… that it gives hope of recovering?*] Yeah. I find that she goes back home with full hope.

**I:** Some of the patients in Rwanda think that they are not provided with enough information about the medidcations. Is this true according to your experience?

**R:** I think we gave them explanations; for me I disagree with them. [**I:** *Huum. Do you think you explain to them?*] Yes, we do.

**I:** Ok... Why is it maybe possible for some to think that they are not provided with anough information? What do you think about it?

**R:** Those who say that they do not have enough information, maybe it depends on the places from which they took medications. I think they may be those who have nedications from other clinics…private pharmacies… However, maybe, it does not mean that they do not receive explanations, I think the reason is… Briefly it’s hard for me to find the reason.

**I:** Huum. Is there any way of working conditions which disturbs the existence of the best conversation between the patient and you?

**R:** Working conditions for nurses? [**I:** *Huum*.] If they do not find enough time, the conversation cannot go well.

**I:** Huum. Is there anything about the functioning of a health center that may prevent from having such enough time as you say? Do you think any cause?

**R:** No other cause, the problem is only not finding such a time. In fact, we should do it but because of having a short time while we have many patients, we do not give them such an opportunity of conversation. Therefore, it’s not our fault, it’s because of a short time.

**I:** Huum. What do you do if a patient requests to be referred to hospital while you think it’s not necessary?

**R:** A patient requesting? [**I:** *Huum*.] You explain to her/him the reason which should motivate the referral, and why you are not allowing to be referred. You take a time and try to talk it to her/him in details. You also immediately gives her/him a rendez-vous for a follow-up; you tell her/him ‘it’s not possible because the symptoms are like these, but if it becomes like this, with such symptoms, come back earlier’

**I:** Huum. Are there any challenges/issues you think they are related health conversation in Kinyarwanda language?

**R:** No problem there. [**I:** *Huum*.] …because it’s their language they understand.

**I:** Huum, the fact that you were taught in French or in English, is it a problem when it comes to having a convenient conversartion with patients in Kinyarwanda?

**R:** (Laughing) No. Oya. It’s not difficult…when we are talking ordinary things, except there is someone who does not understand the language, but we talk in Kinyarwanda, there is no problem.

**I:** Huum. What do you do if you have to explain some clinical/medical terms which are in French or in English but which do not have their equivalent in Kinyarwanda?

**R:** You talk them like they are (without translating)! (*Both laughing*)

**I:** Does the patient understand them?

**R:** (*somehow tired*) S/he understands.

**I:** Huum. Do you mean that you clearly check if the patient fully understood that term you told her/him in Frenc or in English?

**R:** You can let the term in English if you think s/he will understand it! You try to make her/him understand, searching some tentative equivalents, and you interprete for her/him

**I:** Huum. Then, how do you proceed? Tell me in details.

**R:** (*Loudly*) You cannot miss any equivalent! Is there any term which cannot have any equivalent in Kinyarwanda so that s/he can understand? [**I:** *Huum*.] No one.

*(some other male voice talking aside.)*

**I:** Huum. Can you give us an example?

**R:** In fact, for me, I cannot miss any term during the conversation so that I give explanations to a patient. If I prepared well such a conversation, I could not miss any explanation/interpretatio to give to her/him..

**I:** Huum. Are there any other challenges/problems we did not talk about that you encounter during the conversations with patients?

**R:** Other patients?

**I:** Are there any other cahllenges we have not talked about within this interview, that you encounter when talking to patients who come for seeking care to you?

**R:** Maybe, no other challeng.

**I:** Huum. What could be done to improve to improve the skills/knowledge you have concerning talking to patients?

**R:** (*with a low voice*) We should be provided with didactic tools to help us. Our supervisors should also update us about health issues. They should also increase the number of staff at health centers. I think those are the suggestions which can help us in that journey we have ahead of us.

**I:** Patients are different; thus, it obvious that they talk differently. How do you match your communication skills with with the different ways in which patients talk with clinicians?

**R:** Talking to a patient… it depends on the health problem s/he has. After listening to a problem she is seeking care for, you find ways to talk to him/her.

**I:** What could be done for a clinician to help patients to converse with her/him more conveniently during the consultation/examination?

**R:** What could be done for…? [**I:** *For the clinician to help patients to converse with her/him more conveniently during the examination?*] As you are in a private room, you make the patient comfortable and hopeful, and you show her/him that you are attentive and careful. During that time, the conversation becomes good.

**I:** What do you do if a patient cries?

**R:** If a patient cries, you obviously notice what makes her/him crying; (Laughing) you let her/him cry and after crying you comfort her/him saying that’s normal. You tell her/him that the most important is to stay hard in her/his situation and have hope as the problem may be solved soon.

**I:** Is it important to help patients to manage such emotions/feelings resulting from their illiness?

**R:** Huum, it’s important. [**I:** *In which ways?*] If you tell the patient about his/her illiness beforehand… Do you mean emotions/feelings? [**I:** *Huum*] S/he should…you should… Repeat the question so that I understand it!

**I:** Is it important to help patients to manage such emotions or feelings resulting from their illiness?

**R:** You explain to her/him that for example if it’s a chronic disease, s/he should stay calm with such hard situation; that s/he should not have a ‘soft heart’ like this one of crying. S/he should also know that even these emotions may result in another illiness. You try to explain to her/him in this way.

**I:** Is there anything you can do to know if the patient understands what you saying during the conversation?

**R:** Huum. [**I:** *What do you do?*] You ask her/him to repeat what you have been saying so that you ar sure if s/he memorized it. For example you can ask ‘how did I tell you about taking these medications?’ If s/he repeats it in the same way, then you are sure that s/he memorized it.

**I:** Why do you do that?

**R:** I do it to know if s/he has been really listening to what I was saying; to know if we were really together or not during our conversation.

**I:** Should a clinician help help patients to participate in healthcare they are being provided?

**R:** For the clinician to help the patient? [**I:** I mean the patient to participate in clinical/medical care s/he is being provided?] Huum. [**I:** *How?*] You tell and explain to her/ him that even if s/he came for care, s/he will recover from the illiness not only because of the prescribed medications, but there is also his/her participation like the proper diet, drinkings, hygiene…things like that. There are things you tell her/ him to do for her/his health problem. It’s like that.

**I:** Do you think you can add any other thing/item from all of these we have been talking about?

**R:** What you told me… **I:** *Or do you think you can add anything?*] From what you asked me, what I can add is only a suggestion: You told us you are doing a research study; but I think the study has a Principal Investigator and you have objectives. Make an advocacy for us who work from health centers or clinical/medical settings in general: If possible, it should be helpful to increase the number of staff, and be enough in our working sites. Mostly, this would help this point of conversations we were talking about. Another suggestion is that we should be always updated about health information; this would help us on our sklls and have what to tell those who come to us to seek care. Therefore we would be helpful for them

**I:** We asked you many questions, and you gave answers. Howver, do you think there are other questions we can ask to help seek ways of improving the conversations between a clinician/healthcare provider and a patient?

**R:** Other questions? [**I:** Huum.] There are many questions, there is no way you can miss questions! I think there are others.

**I:** Huum. Could you give an example of another question to ask? Like questions maybe we can add to these ones I have been asking about how to improve the conversations between a patient and a clinician/healthcare provider?

R: I do not have any question right now in my mind!

**I:** Ok. Thank you so much!

**R:** Hum. You are welcome.
